# The Tautomeric State of *N*^4^-Hydroxycytidine within Base-Paired RNA

**DOI:** 10.1021/acscentsci.4c00146

**Published:** 2024-04-25

**Authors:** Irene Bessi, Carina Stiller, Till Schroeder, Benedikt Schäd, Matthias Grüne, Julia Dietzsch, Claudia Höbartner

**Affiliations:** † Institute of Organic Chemistry, Julius-Maximilians-University Würzburg, Am Hubland, 97074 Würzburg, Bavaria, Germany; ‡ Center for Nanosystems Chemistry, Julius-Maximilians-University Würzburg, 97074 Würzburg, Bavaria, Germany

## Abstract

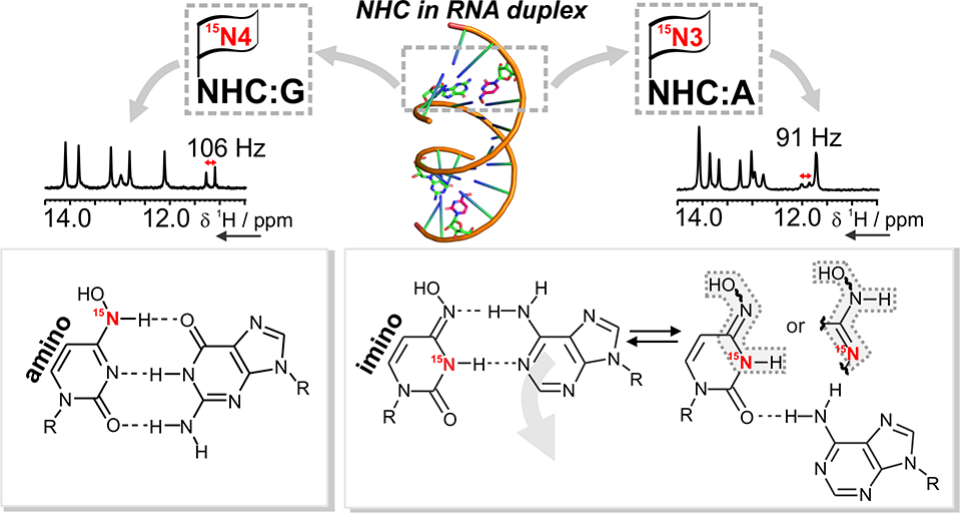

Antiviral nucleoside analogues (e.g., Molnupiravir, Remdesivir) played key roles in the treatment of COVID-19 by targeting SARS-CoV-2 RNA-dependent RNA polymerase (RdRp). The nucleoside of Molnupiravir, *N*^4^-hydroxycytidine (NHC), exists in two tautomeric forms that pair either with G or A within the RdRp active site, causing an accumulation of viral RNA mutations during replication. Detailed insights into the tautomeric states within base pairs and the structural influence of NHC in RNA are still missing. In this study, we investigate the properties of NHC:G and NHC:A base pairs in a self-complementary RNA duplex by UV thermal melting and NMR spectroscopy using atom-specifically ^15^N-labeled versions of NHC that were incorporated into oligonucleotides by solid-phase synthesis. NMR analysis revealed that NHC forms a Watson–Crick base pair with G via its amino form, whereas two equally populated conformations were detected for the NHC:A base pair: a weakly hydrogen-bonded Watson–Crick base pair with NHC in the imino form and another conformation with A shifted toward the minor groove. Moreover, we found a variable influence of NHC:G and NHC:A base pairs on the neighboring duplex environment. This study provides conclusive experimental evidence for the existence of two tautomeric forms of NHC within RNA base pairs.

## Abstract 2

NMR of RNA duplexes containing atom-specifically ^15^N-labeled *N*^4^-hydroxycytidine (NHC) enables us to determine the tautomeric form and the base-pairing pattern of NHC:G and NHC:A.

## Introduction

**Figure 1:**
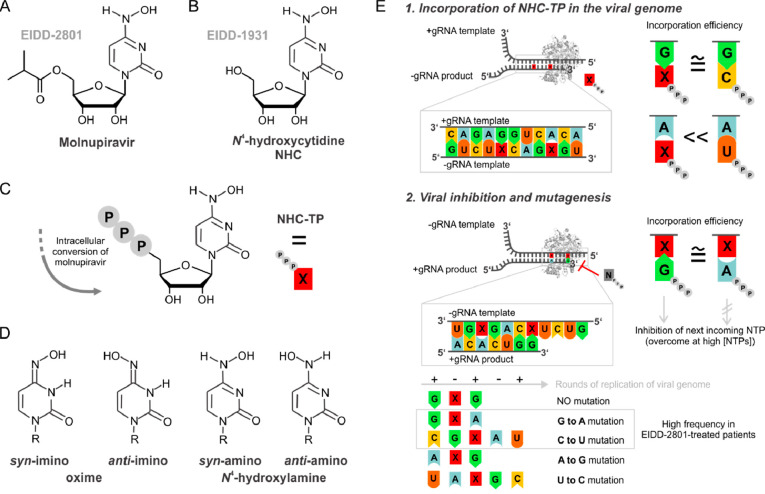
NHC tautomerism plays a key role in Molnupiravir-induced lethal mutagenesis. Structure of Molnupiravir (A) and NHC (B). (C) Intracellular conversion of Molnupiravir to NHC-TP. (D) Imino-amino tautomers of β-d-*N*^4^-hydroxycytidine (NHC) with respective OH/N3 *syn* and *anti* stereoisomers (R = β-d-ribose). (E) Two-step mechanism for NHC-induced mutagenesis, according to Kabinger et al.^10^ and Gordon et al.^11^

SARS-CoV-2 is a highly infectious virus, with multiple variants arising. A significant endeavor has been dedicated to the development of vaccines and treatment strategies to block different stages of the viral life cycle. One main target for antiviral agents is the RNA-dependent RNA polymerase (RdRp), which is responsible for the replication and transcription of the viral RNA.^1^ Molnupiravir (EIDD-2801, Figure 1A), a broad-spectrum antiviral nucleoside analogue,^2^ is a promising candidate for targeting RdRp, being resistant to the viral exoribonuclease proofreading activity.^3^ After its oral administration^2^ as an isopropyl ester prodrug of β-d-*N*^4^-hydroxycytidine (EIDD-1931, NHC, Figure 1B), it is intracellularly activated to the *N*^4^-hydroxycytidine-triphosphate (NHC-TP, Figure 1C),^4^ which can then be used by the RdRp as a substrate for viral RNA replication. Although phase 3 MOVe-OUT clinical trials provided evidence for justifying the use of Molnupiravir for the early treatment of nonhospitalized patients with mild to moderate COVID-19,^5^ a subsequent PANORAMIC trial showed that Molnupiravir treatment did not reduce the frequency of COVID-19-associated hospitalizations or death in the community of high-risk vaccinated adults.^6^ Still, it is associated with faster recovery time, reduced viral load, and reduced contact with general practitioner services.^6^ It was one of the first orally bioavailable antivirals marketed in many countries, but due to the results of the most recent clinical trials, the European Medicines Agency recommended refusing its marketing authorization (EMA/116128/2023). Concerns have been raised about potential off-target effects, such as mutagenicity in host cells, and about Molnupiravir potentially accelerating SARS-CoV-2 evolution.^7−9^

The antiviral activity of Molnupiravir results from the lethal accumulation of viral RNA mutations, which is due to NHC forming different tautomers (Figure 1D). The mutagenic mechanism relies on NHC ambiguous base pairing and proceeds via two steps (Figure 1E).^10,11^ NHC-TP can be incorporated by the RdRp instead of C or U opposite G or A of the RNA template. When the NHC-containing strand is then used as a template, G or A can be incorporated in the positive strands with comparable efficiency. Incorporation of G opposite to NHC slows down the incorporation of the next nucleotide, but this partial inhibitory effect can be overcome by increasing the nucleoside triphosphates (NTPs) concentration.^11^ RNA synthesis rate is less affected when A is incorporated opposite to NHC in the template, leading to mutations. In fact, G to A (G:NHC:A) and C to U (C:G:NHC:A:U) transition mutations are observed in *in vivo* studies.^3,12^ The formation of stable NHC:G and NHC:A base pairs within the active site of RdRp was also confirmed by cryo-EM of RdRp-RNA complexes.^10^ However, the base-pair geometries modeled into the cryo-EM densities deviated slightly from idealized Watson–Crick (WC) geometry, and biochemical studies suggested that NHC is not perfectly mimicking C or U.^10,11^

The tautomeric states of *N*^4^-substituted cytosine derivatives have been investigated for more than 50 years,^13^ followed by studies based on NMR, IR, and crystallography. For the free nucleoside, the equilibrium of the two tautomeric forms, namely, amino and imino forms, is dependent on the solvent.^14^ In both aqueous and nonaqueous media, the imino form is predominant.^13,14^ Self-association of 1,5-dimethyl-*N*^4^-hydroxycytosine reported by X-ray and IR-spectroscopy revealed that an intramolecular hydrogen bond (N^4^–OH···N3) stabilizes the imino form with the OH *syn* to N3.^15,16^ The imino-*syn* form has been also observed by NMR in organic solvents for 1-methyl-*N*^4^-methoxycytosine base paired with adenine via trans Watson–Crick/Watson–Crick hydrogen bonding.^17^ Early quantum-chemical calculations performed in the gas phase on *N*^4^-hydroxycytosine also concluded that the *syn*-imino form is the energetically most favorable and, due to the high energy barrier, cannot flip spontaneously into the *anti*-imino form. The authors suggest that the presence of *N*^4^-hydroxycytosine in the amino form is questionable and the reported ambivalent base pairing capability should be explained by the exclusive presence of the imino form.^18^ Recent theoretical studies performed in a water environment suggest that the energy difference between *anti*-imino and *anti*-amino forms of NHC-monophosphate is small enough to allow the existence of both tautomers after its administration *in vivo*, whereas the energy gap between the two tautomers after building into the RNA greatly increases in favor of the imino form.^19^ In an oligomer context, mainly reports on the structure and stability of DNA duplexes containing *N*^4^-methoxycytidine (NMC) were published. In the crystalline state of a Z-type duplex, the G:NMC was present as a wobble base pair, with NMC in the imino form and the methoxy group *syn* to N3.^20^ According to NMR studies, NMC is able to base pair with G using both its tautomers, resulting in a slow exchange between Watson–Crick and wobble base pairing, with both *syn* and *anti* geometrical isomers being populated in the wobble arrangement.^21,22^ Furthermore, when adenine is the counterpart, NMC adopts predominantly its *anti*-imino form in a Watson–Crick base pair.^23^ Thus, in solution, NMC is present within DNA base pairs in both tautomeric forms, and the tautomeric equilibrium is influenced by the base-pairing partner. Although at the monomer level the *syn*-imino form is the favored one, it was suggested that upon enzymatic incorporation of NMC into an oligonucleotide, the methoxy group is constrained to the *anti*-form by the RNA polymerase, to ensure base pairing with A.^24^

Despite the plethora of studies available for NMC in DNA, a detailed structural characterization of the tautomeric equilibrium of NHC within base pairs, in particular, in RNA, is still missing, although this is essential for a detailed understanding of the principles of Molnupiravir action and for assessing its safety. The cryo-EM structure from our earlier study suggests for NHC:G and NHC:A the formation of Watson–Crick base pairs, with NHC in amino and imino form, respectively.^10^ However, the cryo-EM density does not fully exclude the possibility of the imino tautomer of NHC forming a wobble pair with G. We sought to gain a deeper understanding of the specific interactions between NHC and its corresponding partners as well as to determine whether NHC indeed forms both tautomers within base pairs.

We chose a 12-bp RNA duplex with the self-complementary sequence (known as the Dickerson-Drew dodecamer (DD) sequence) as a model system to investigate the influence of NHC on the structure and stability of the duplex as well as NHC base pairing ability with A and G. NHC phosphoramidite was synthesized and incorporated into RNA oligonucleotides via solid-phase synthesis.^10,25^ UV thermal denaturation experiments and NMR studies with atom-specifically ^15^N-labeled NHC-modified RNA were used to elucidate thermodynamic properties and hydrogen bonding patterns formed by NHC in base pairs with A and G.

## Results and Discussion

**Figure 2:**
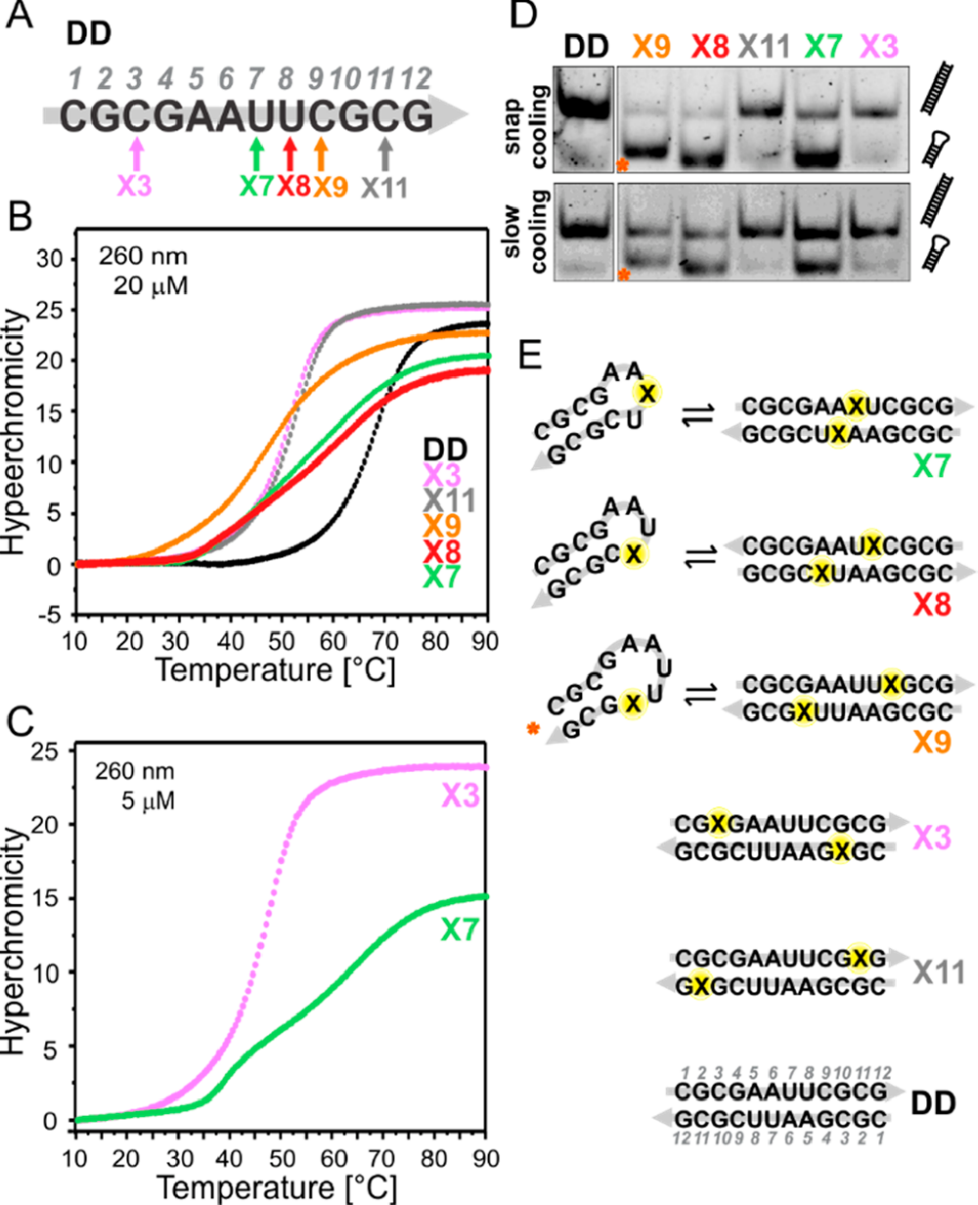
Position and the distance of the tandem modifications influence the duplex-hairpin equilibrium. (A) DD sequence with NHC (X) at indicated positions. (B) UV melting curves of the DD variants (20 μM duplex). (C) Comparison of X3 and X7 UV melting profile at 5 μM duplex concentration. (D) Effect of annealing protocol on the duplex-hairpin equilibrium analyzed by native gel electrophoresis (20% PAA). (top) Snap cooling. (bottom) Slow cooling to room T. (E) Scheme of the proposed conformations for the analyzed sequences in comparison with the reference DD (numbering indicated in gray).

The self-complementary DD RNA sequence 5′-CGCGAAUUCGCG-3′ can form duplex or hairpin structures in varying ratios, as previously investigated with methylated nucleosides.^26^ Here, we analyzed the influence of NHC at various positions in the DD RNA on the duplex-hairpin equilibrium. We systematically replaced NHC (abbreviated as X, for space reasons) with either cytidine or uridine to obtain the respective X:G and X:A base pair (Table 1, Table S1, and Figure 2A). Due to the palindromic nature of the DD sequence, replacement of one nucleotide with X results in tandem modified base pairs in the duplex. Hairpin and duplex structures were assigned by UV melting studies, native gel mobility assay, and imino 1D ^1^H NMR signatures (Figures 2 and S3).

**Table 1:** Dickerson-Drew-Derived RNA Oligonucleotides with NHC (X) at Different Positions^a^

Name	modified bp	5′-sequence-3′	*T*_M_ [20 μM]	*T*_M_ [2 μM]	Δ*H*^0^ (kcal/mol)	Δ*S*^0^ (cal/(mol*K))	Δ*G*^298^ (kcal/mol)
DD		CGCGAAUUCGCG	67.2	60.7	–81.1 ± 0.4	–218.4 ± 1.1	–16.0 ± 0.5
U3	UG	CGUGAAUUCGCG	49.0	44.4	–101.9 ± 1.1	–296.1 ± 3.1	–13.6 ± 1.4
X3	XG	CGXGAAUUCGCG	51.1	46.6	–112.4 ± 2.1	–327.1 ± 6.0	–14.9 ± 2.7
U3A10	UA	CGUGAAUUCACG	59.1	54.1	–102.2 ± 1.8	–287.8 ± 5.1	–16.4 ± 2.4
X3A10	XA	CGXGAAUUCACG	43.8	38.7	–89.1 ± 0.8	–261.3 ± 2.2	–11.2 ± 1.0
C3A10	CA	CGCGAAUUCACG	45.7	40.5	–91.4 ± 1.0	–266.9 ± 3.1	–11.8 ± 1.4
X7	XA	CGCGAAXUCGCG	61.7	39.9/64.5			
X8	XA	CGCGAAUXCGCG	62.4	38.7/63.8			
X9	XG	CGCGAAUUXGCG	47.6	35.7/55.3			
X11	XG	CGCGAAUUCGXG	51.9	47.0	–101.1 ± 1.5	–291.3 ± 4.3	–14.3 ± 1.9

a Modifications compared to the original Dickerson-Drew dodecamer (DD) are underlined. Thermodynamic parameters Δ*H*^0^ and Δ*S*^0^ derived from Van’t Hoff analysis with five duplex concentrations (1, 2, 5, 10, and 20 μM). Δ*G*^298^ calculated at 298 K.

UV melting curves showed that the introduction of one modification consistently leads to destabilization compared with the unmodified reference DD (Figure 2 and Table 1). CG-to-XG replacement at more terminal positions (X3, X11; Δ*T*_M_ ≈ −15 °C) was less destabilizing than that at more internal positions (X9; Δ*T*_M_ ≈ −20 °C). The proximity of the tandem modifications correlates with increased destabilization. At 20 μM duplex concentration, all of the sequences showed a concentration-dependent monophasic melting profile, pointing at the formation of a duplex structure (Figures 2B and S1; Table S2). However, the melting profiles of X7, X8, and X9, with NHC at more central positions of the DD sequence, were distinctly shallower than X3 and X11. In fact, at lower concentrations the melting profiles of X7, X8, and X9 were biphasic (Figure S1). Figure 2C displays a comparison of the melting transitions at lower concentrations for the representative sequences X3 and X7. The biphasic melting behavior is attributed to a duplex-hairpin equilibrium (Figure 2E).^26^ The lower thermal stability of X9 in the hairpin form (Δ*T*_M_ ≈ −10 °C compared to X7 and X8) may be attributed to a destabilized stem of three GC base pairs, with G:NHC as a very weak stem-loop closing base pair. Interestingly, electrophoretic mobility shift assays under native conditions showed that the band assigned to the hairpin structure of X9 ran slower compared to all of the other sequences (Figure 2D, orange asterisk), in line with the hypothesis that the X9 hairpin has a different secondary structure (3 base pair stem and loose loop, Figure 2E). Furthermore, in the modified sequences containing NHC at more internal positions (X7, X8, and X9) the relative population of duplex and hairpin structures could be shifted by changing the annealing protocol, with snap cooling resulting in an increase of the hairpin population (Figure 2D). On the other hand, the DD modified at more terminal positions (X3 and X11) formed a duplex, regardless of the annealing procedure.

1D ^1^H NMR spectra were recorded on all modified DD RNAs over the temperature range 283–318 K (Figure S3) and, also under the NMR experimental conditions, conformational heterogeneity (additional imino signals observed in X7, X8, and X9 compared to DD) was observed in the imino region when NHC was incorporated at more internal positions. However, only the imino ^1^H NMR signature of the duplex was observed when the modification was at more terminal positions (detailed discussion in the following section).

**Figure 3:**
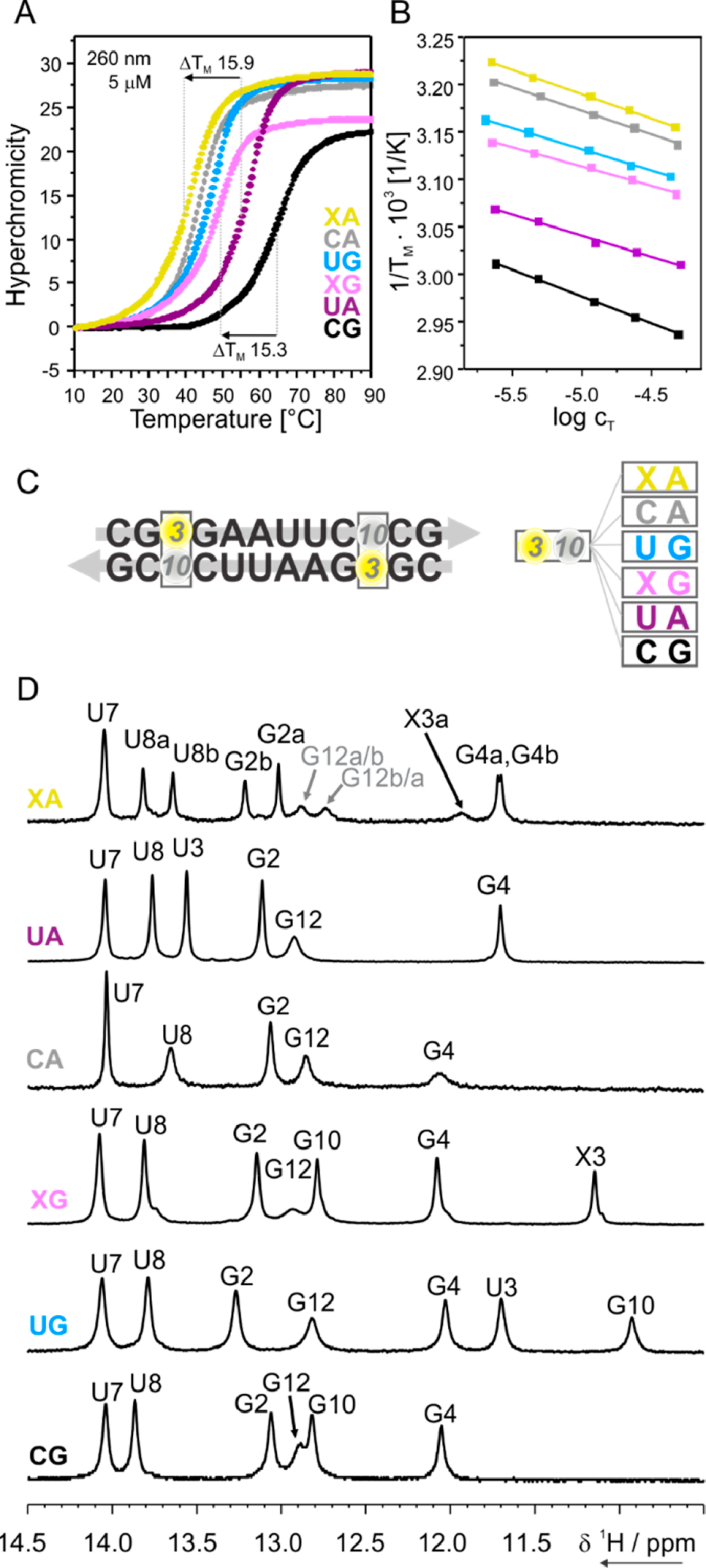
UV thermal melting analysis and NMR studies of the DD duplexes modified at position 3/10. UV melting curves (A) and van’t Hoff analysis (B) of DD duplexes containing the modifications shown in (C). (D) Assignment of the imino region of the 1D ^1^H NMR spectra of the modified DD duplexes recorded at 283 K and 600 MHz.

In order to nail down the geometry of NHC in a base pair, we performed comprehensive UV and NMR analyses on the DD duplexes containing the modification X at position 3, with either G or A as the base pair partner on the opposite strand at position 10 (XG and XA, respectively). The effect of the modification was analyzed in comparison to the duplexes containing canonical Watson–Crick CG or UA base pairs, UG wobble base pairs, or CA mismatches at the same position (Figure 3). All of the samples with modification at positions 3/10 (Figure 3C) exhibited monophasic, concentration-dependent melting profiles (Figures 3A and S2), reflecting the formation of duplex structures.

Introduction of the modification opposite to G (XG) or to A (XA) resulted in a similar destabilization (about 15 °C) of the respective reference duplexes containing CG or UA, as indicated in Figure 3A. Van’t Hoff analysis of the *T*_M_ concentration dependence (Figure 3B; Tables 1 and S2) allowed us to determine the thermodynamic stability of the duplexes. Introduction of a tandem XA base pair led to the largest thermal destabilization (*T*_M_ = 40.6 °C at 5 μM duplex) and was associated with the least favorable free energy ( = −11.2 ± 1.0 kcal/mol). The destabilization introduced by tandem XA is comparable to the one observed in the presence of two CA mismatches ( = −11.8 ± 1.4 kcal/mol).

Figure 3D shows the assignment of the imino region of all the RNA duplexes modified at position 3/10, resulting from the analysis of 2D ^1^H,^1^H NOESY (Figures S4 & S5) and 2D ^1^H,^13^C HSQC spectra at different temperatures. The imino region of the ^1^H NMR spectrum provides information about the number of the imino protons involved in base pairing as well as on the base pair pattern. Due to the symmetry of the sequence, six signals were expected in the Watson–Crick imino region (12–14 ppm) for the unmodified Dickerson-Drew dodecamer CG. Indeed, five sharp signals and one broad signal, arising from the terminal G12, were detected, which is consistent with duplex formation (Figure 3D, bottom). Interestingly, the XG imino signature shows at 11.15 ppm an additional signal compared to CG, which might stem from the modification X3, as indicated in Figure 3D. However, on the basis solely of NOESY data, it was not possible to unambiguously assign the signals to a specific tautomeric form (i.e., imino or amino proton). For example, the additional signal observed at 11.15 ppm in XG compared to CG presents a strong NOE cross peak to the opposite base G10 (Figure S4). This could fit with the formation of a wobble base pair with NHC in imino form (see chemical shift similarity of G10 in UG and X3 in XG, Figure 3D; UG and XG 2D ^1^H,^1^H NOESY spectra, Figure S4). On the other hand, strongly downfield-shifted amino proton signals have been already reported for protonated cytidine interacting with the Hoogsteen side of guanine in C^+^·G-C triplets^27^ or C^+^·m1G base pair^28^ as well as for *N*^4^-methoxycytidine (NMC) base paired with G in a heptamer duplex.^22^ Therefore, a Watson–Crick base pair with NHC in amino form could also result in the observed signal at 11.15 ppm with a strong NOE cross peak to G10.

Furthermore, the imino region of the ^1^H NMR spectrum of XA shows additional signals compared to those of the duplex containing a canonical UA Watson–Crick base pair at the same position (Figure 3D), suggesting the presence of conformational heterogeneity. Indeed, analysis of the 2D ^1^H,^1^H NOESY spectrum revealed the presence of two conformations, named “a” and “b” in the following, almost equally populated and in slow exchange on the NMR time scale (Figures 5A, S4, and S8, gray labels). On the other side, the imino region of the ^1^H NMR spectrum of CA shows only five signals, with no imino or amino signal detected for the CA mismatch and pronounced broadening of U8 and G4 imino, compared to XA (see also A10 C2–H2 signals in ^1^H,^13^C HSQC, detected in XA but broadened beyond detection in CA, Figure S6).

We decided to solve the assignment ambiguities of NHC base pairs and elucidate the molecular details of NHC:A conformational heterogeneity by atom-specific isotope labeling of NHC. The ^15^N isotope label was introduced in NHC either at position N4 or at position N3, and the resulting phosphoramidites were then used for solid-phase synthesis to incorporate in XG or in XA (Table S1). The synthesis of the phosphoramidites is described in the Supporting Information. Briefly, ^15^N at N4 was introduced by reacting 5′-*O*-DMT-2′-*O*-TOM-*O*4-chlorophenyluridine with ^15^NH_2_OH (Scheme S1), followed by benzoyl protection and phosphitylation. Similarly, the NHC building block with ^15^N label at position N3 was obtained from a ^15^N(3)-labeled uridine, which was prepared as described by Micura and co-workers (Scheme S2).^29^ At the nucleoside level and in organic solvents, NHC is present in the imino form, as shown by the assignment of the 2D ^1^H,^15^N HSQC and ^1^H,^15^N HMBC spectra (Figure S7).

**Figure 4:**
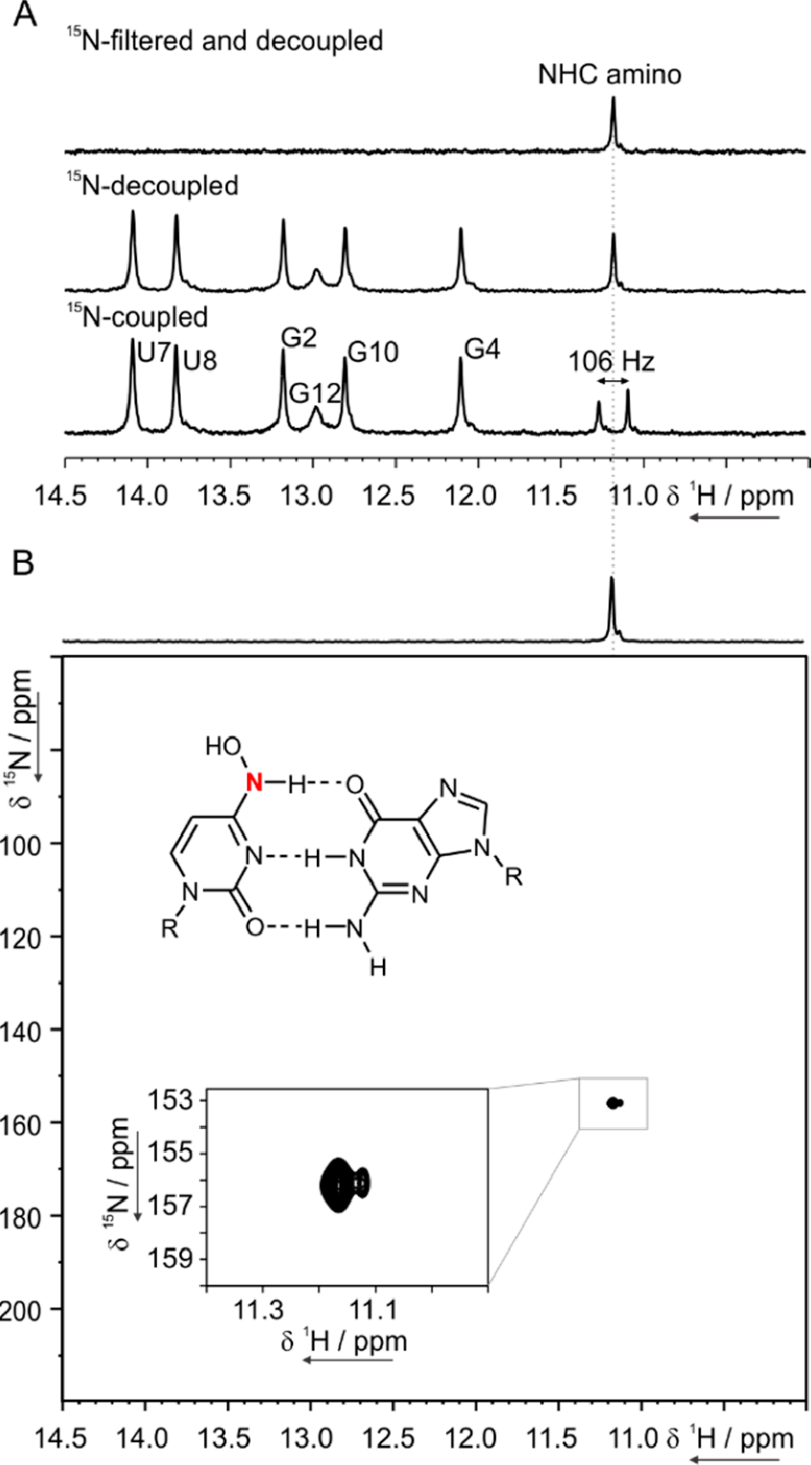
NMR studies on XG 3/10 DD RNA containing NHC with ^15^N-labeled N4 (red). (A) Imino region of the 1D ^1^H NMR spectrum of the XG without ^15^N decoupling (bottom), with ^15^N decoupling (mid), and with ^15^N filter and ^15^N decoupling (top). (B) 2D ^1^H,^15^N HSQC, with a zoomed-in view of the peak. Proposed base pair pattern for NHC:G, with NHC in the amino form. All spectra were recorded at 283 K and 600 MHz.

First, we focused our NMR analysis on the DD RNA containing the XG base pair with ^15^N at N4 (Figure 4). 1D ^1^H spectra acquired without ^15^N decoupling, with ^15^N decoupling, and with ^15^N filter and ^15^N decoupling are shown in Figure 4A. In absence of ^15^N decoupling (Figure 4A, bottom) a 106 Hz splitting of the signal at 11.15 ppm was observed, which is consistent with the one-bond scalar coupling constants ^1^*J*_NH_ reported for uridine and guanosine.^30^ Accordingly, the ^15^N-filtered and ^15^N-decoupled 1D ^1^H spectrum (Figure 4A, top) showed only one peak at 11.15 ppm. These results provide conclusive evidence for the formation in solution of a Watson–Crick NHC:G base pair with NHC in the amino form (Figure 4B). The site-specific isotope labeling allowed us to determine the ^15^N chemical shift of the NHC amino N4 involved in the Watson–Crick base pair with G. The ^1^H,^15^N HSQC experiment shows that the proton resonating at 11.15 ppm is correlated with a nitrogen resonating at 156 ppm (Figure 4B), which is markedly different from the average chemical shift reported in the BMRB for cytidine N4.^31^ In fact, the amino N4 of an unmodified cytidine involved in a canonical WC base pair typically resonates around 98 ppm and can be downfield -shifted up to 115 ppm in C^+^·G-C triples.^27,32−34^ However, earlier reports on free nucleosides in 100% *d*_6_-DMSO showed that modifications at N4 may lead to comparable large downfield shifts.^35^ It is also worth noting that in a duplex context, NMC paired to G was reported to form simultaneously the two tautomeric forms,^22,21^ whereas NHC is here shown to be present only in the amino form. As depicted in the zoomed-in view of Figure 4B, a second much less intense peak with a very similar chemical shift was detected in the ^1^H,^15^N HSQC, hinting at the presence of a second minor conformation. Thus, NHC:G forms only a Watson–Crick base pair and is present also in another low-populated (5%) state with very similar NMR parameters and in slow exchange with the major form on the NMR time scale.

**Figure 5:**
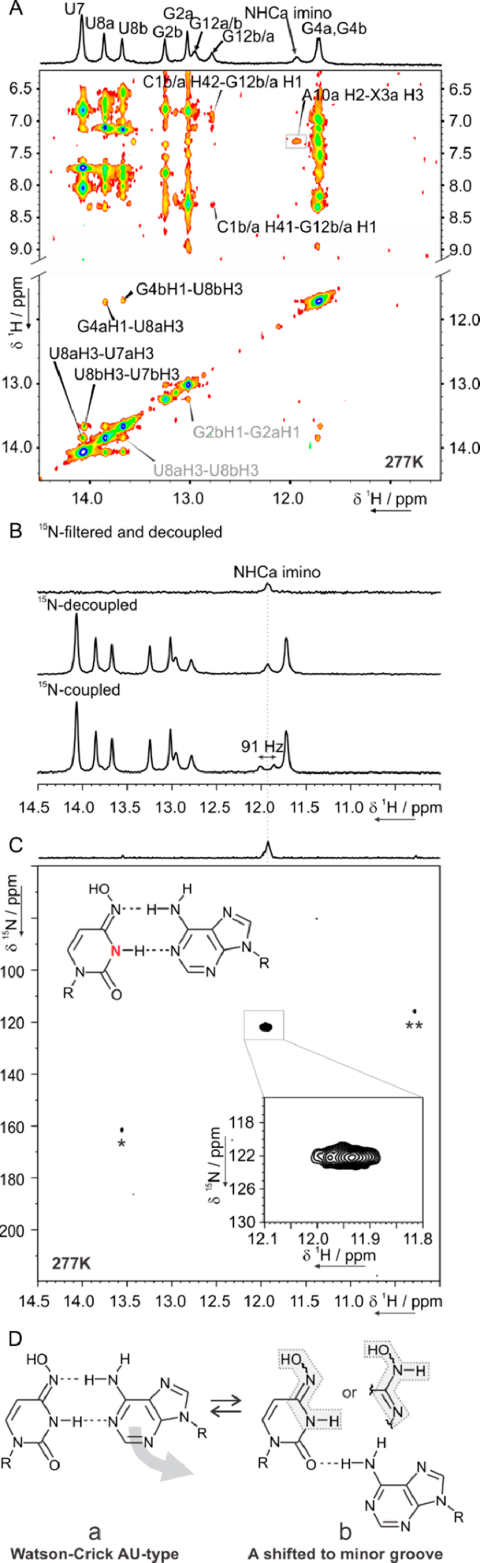
NMR studies on the XA Dickerson-Drew variant containing NHC with ^15^N-labeled N3 or N4. (A) Imino and imino-amino regions of the 2D ^1^H,^1^H NOESY spectrum (200 ms mixing time) of XA (^15^N(4)-labeled) with assignment. Exchange peaks are labeled in gray. (B) Imino region of the 1D ^1^H NMR spectrum of the (^15^N(3)-labeled) XA without ^15^N decoupling (bottom), with ^15^N decoupling (mid), and with ^15^N filter and ^15^N decoupling (top). (C) 2D ^1^H,^15^N HSQC spectrum of the (^15^N(3)-labeled) XA, with zoom on the major peak arising from conformation “a” with NHC:A in Watson–Crick arrangement and NHC in the imino form (see scheme). *Residual uridine byproduct of NHC deprotection and ** minor, lowly populated conformation. All of the spectra were recorded at 277 K and 600 MHz. (D) Proposed equilibrium in slow exchange between NHC:A Watson–Crick (a) and NHC:A with A shifted toward the minor groove (b).

Next, we focused our NMR investigations on the DD RNA variant containing XA base pair with NHC ^15^N-labeled either at position N4 (2D ^1^H,^1^H-NOESY spectra Figure 5A, ^15^N NMR data not shown) or at position N3 (Figure 5B,C). The three broad exchangeable protons observed for XA between 12 and 13 ppm in the imino region were detected only at low temperature and are assigned as indicated based on the NOE cross peaks observed in the imino/aromatic-amino region (^15^N(4)-labeled variant, Figure 5A, Figure S9 for details). The broad peak at 11.9 ppm is assigned to X3 H3 of conformation “a” due to the presence of a cross peak to A10a H2 (gray box in Figure 5A) as well as to the similarity of the chemical shift to the one reported for NMC Watson–Crick base paired with A in a DNA duplex heptamer.^36^ Furthermore, the chemical shift perturbation analysis (CSP) of each of the two conformations of XA, relative to the reference UA dodecamer (details in Figure S10), shows that conformation “a” is less perturbed than conformation “b”. Taken together, these observations suggest that conformation “a” features NHC in the imino form base-paired to A in a Watson–Crick style with the −OH *anti* to N3.

The NOESY-based assignment of conformation “a” was confirmed using ^15^N-filtered experiments recorded on the sample containing ^15^N(3)-labeled NHC (Figure 5B). 1D ^1^H spectra recorded with ^15^N decoupling and with ^15^N filter show that the broad signal at 11.9 ppm stems from the imino proton of NHC. The corresponding scalar coupling is 91 Hz and is again in agreement with the ^1^*J*_NH_ values reported for uridine and guanosine. The ^1^H,^15^N HSQC spectrum reveals that the signal at 11.9 ppm is correlated to a ^15^N atom resonating at 122 ppm (Figure 5C), which is very close to the 118 ppm observed for the free nucleoside in the imino form (Figure S7). Most importantly, the NHC imino signal of conformation “a” was detected only at low temperature and broadened beyond detection at room temperature, implying a very weak base pairing with A.

The structure of NHC:A in the alternative conformation “b”, which accounts for the remaining 50% population, can be hypothesized based on the following observations. The broadened ^1^H,^15^N HSQC peak at 122 ppm in the ^15^N dimension must stem exclusively from conformation “a” since its correlated proton forms in the NOESY a cross peak to A10H2a but not to A10H2b (as expected in a Watson–Crick base pair) or to A10H8b (as expected in an XA Hoogsteen-type). The minor peaks observed in the ^1^H,^15^N HSQC result from a small amount of NHC-to-U conversion during oligo deprotection (*) and from a minor conformation (**) which cannot account for the remaining ∼50% populated state “b”. So “b” cannot feature NHC with the imino proton involved in a stable H-bond. 1D ^1^H spectra without ^15^N decoupling, with ^15^N decoupling, and with ^15^N filter and ^15^N decoupling were recorded also on the XA variant containing ^15^N label at N4 of NHC (data not shown). No changes upon ^15^N decoupling and no signal with a ^15^N filter were observed in the 1D ^1^H imino region. Thus, conformation “b” cannot present NHC in amino form with N4 amino proton protected from exchange with water. Furthermore, the ^13^C chemical shift of C2 A10 in conformation b (154.8 ppm, Figure S6) shows that A10b is not protonated at N1, allowing us to exclude a conceivable NHC:A^+^ wobble base pair, with NHC in the amino form and A protonated at N1. Keeping in mind the large CSPs observed for “b” in the C9-A10-G11 tract (Figure S10), we suggest that the NHC:A in conformation “b” features A10 shifted toward the minor groove (Figure 5D).^23^ However, our data cannot precisely define the tautomeric form adopted by the NHC in this arrangement.

Taken together, the NMR data confirm that NHC is able to weakly pair with A via a Watson–Crick type hydrogen bonding pattern using its imino form with −OH *anti* to N3. However, in contrast with what was reported for NMC base paired with A,^36^ we observed conformational heterogeneity of NHC base paired with A, namely, the presence of two equally populated conformations in slow exchange on the NMR time scale. We propose that the second conformation presents A10 pushed toward the minor groove and is weakly base-paired to NHC O2 via N6–H61 (Figure 5D).

**Figure 6:**
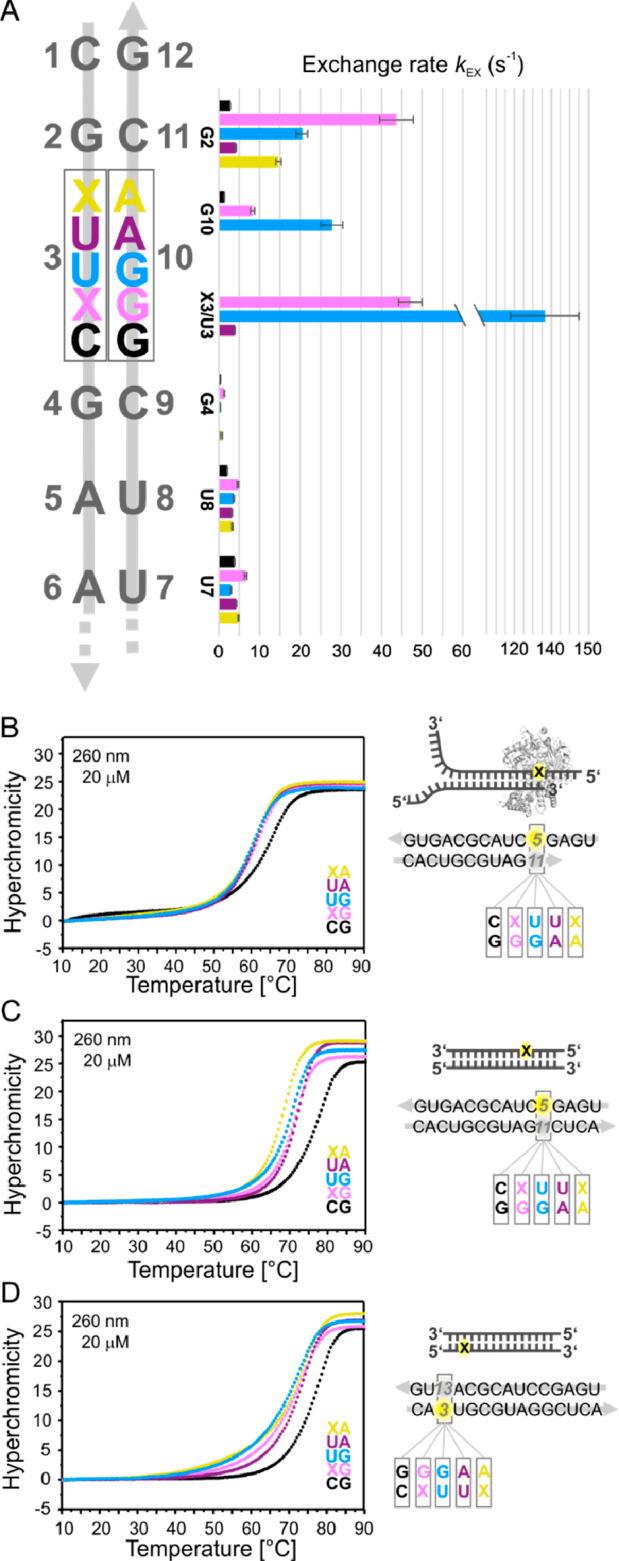
NHC effect on the RdRp mechanism. (A) Imino-water exchange rate (*k*_EX_) of the Dickerson-Drew duplex variants modified at position 3/10 and determined at 298 K. Imino exchange rate in duplex XA reported for conformation “a” and not determined for NHC imino due to fast exchange. NHC exchange rate in duplex XG refers to the amino proton. UV melting curves of the RdRp substrate directly after incorporation of G/A opposite to X template (B) and after full elongation of the substrate containing G/A opposite to X at more internal (C) or more terminal (D) positions.

We further investigated at the single base pair level the stability of the RNA duplexes modified at position 3/10 with the XG and XA base pair. Imino-exchange rates (*k*_EX_) with water were determined by NMR using CLEANEX-PM experiments (Figures 6A and S11). As already discussed, the imino proton signal of NHC in the NHC:A Watson–Crick base pair (duplex XA) was broadened beyond detection at room temperature; therefore, its *k*_EX_ was too fast to be determined. On the other hand, as indicated by the exceptional sharpness of its NMR proton signal, it was possible to determine the exchange rate of NHC amino proton engaged in Watson–Crick base pair with G (duplex XG). The overall trend of the *k*_EX_ observed for each duplex at the single nucleotide level reflects the thermodynamic stability determined by UV, with CG > UA > XG ∼ UG > XA. Interestingly, the introduction of an NHC:G or NHC:A base pair also has a significant effect on the adjacent base pairs, particularly on G2:C11. Indeed, we observed that NHC induces a destabilization of the downstream base pair (G2:C11 in the DD model; *k*_EX_^G2,CG^ = 2.79 ± 0.08, *k*_EX_^G2,XG^ = 43.70 ± 4.16, and *k*_EX_^G2,XA^ = 14.64 ± 0.65, Figures 6A and S11). The G2:C11 destabilization induced by NHC:G compared to the standard Watson–Crick base pair C:G was ∼4.5 times larger than the destabilization induced by NHC:A compared to U:A. Thus, the imino exchange rate measured by NMR suggests that NHC:G impairs the downstream base pair stability to a much larger extent compared to NHC:A, whereas both have very minor effects on the upstream base pair. These data may explain the biochemical observations reported by Gordon et al., that an NHC:G base pair with NHC embedded in the template strand slows down or partially inhibits the incorporation of the next incoming nucleotide, while the corresponding NHC:A base pair has less effect on the continuation of RdRp-mediated RNA synthesis.^11^

To further examine the effect of NHC:G and NHC:A base pairs on RNA structure and stability, we synthesized RNA oligonucleotides resembling different stages of viral replication and analyzed their thermal melting by UV (Figures 6B–D and S12–S14; Tables S3 & S4). NHC was incorporated at position 5 of the same 15mer sequence used as RdRp template substrate for the cryo-EM studies.^10^ The template annealed with a complementary 11mer resembles the stage of the virus replication, directly after incorporation of G or A opposite to templating NHC (Figure 6B), resulting in a base-paired region with 4 nucleotides overhang. At this stage, incorporation of G opposite to NHC template instead of C template resulted in a Δ*T*_M_ ≈ −3 °C, whereas no destabilization was observed upon replacing a UA with an XA base pair in the same structural context. Still, the global thermal stability of the duplex directly after incorporation of G or A opposite to X was comparable (Table S4). In the corresponding fully elongated duplex (Figure 6C), replacement of CG with XG base pair resulted in a duplex global thermal destabilization of 6 °C, whereas a U-to-X mutation opposite to A was associated with a duplex Δ*T*_M_ ≈ −4 °C. In contrast, if the modification was incorporated at more terminal positions (Figure 6D), the destabilization was less pronounced (Δ*T*_M CG-XG_ ≈ −4 °C and Δ*T*_M UA-XA_ ≈ 0 °C). As already observed for the DD variants X3 and X11, the modification at a more internal position produced a larger thermal destabilization of the duplex. The global thermal destabilization must result not only from the intrinsic stability difference between XA and XG base pair (XA ≪ XG from NMR data) but also from effects on nearest and non-nearest neighbors due to the propagation of modification-induced distortion through the helix.

## Conclusion

In summary, we have shown that when NHC is engaged in a base pair, it can adopt either the imino or amino tautomeric form, adapting to the base-pairing partner. Incorporation of atom-specifically ^15^N-labeled NHC phosphoramidite into the RNA model duplex allowed us to unambiguously determine by NMR the tautomeric form involved in the base pair with guanine or adenine. The NHC:G base pair resembles a canonical Watson–Crick C:G base pair and features NHC in the amino form, confirmed by the assignment of the unusual N4 amino proton at 11.1 ppm. The NHC:A base pair exists as a mixture of two states in a 1:1 population ratio with slow exchange on the NMR time scale. The first conformation features NHC in the imino form very weakly hydrogen-bonded to A in a Watson–Crick U:A-type arrangement. The second conformation is a wobble-type with A shifted to the minor groove and weakly hydrogen bonded to NHC O2 via N6–H6. The global destabilization observed by UV thermal melting analysis upon incorporation of the modification comes along with major effects on the stability of the adjacent base pairs, as shown by measuring the imino exchange rates. The observed trend of increasing destabilization of the base pair downstream to the modification site (XG > XA) seems consistent with earlier studies on extension kinetics.^11^ Our results shed light for the first time on the tautomeric forms engaged in NHC base pairing and contribute to a deeper understanding of the NHC mutagenic mechanism of action.

## Methods

The unmodified 5′-*O*-DMT-2′-*O*-TOM-protected phosphoramidites for solid-phase synthesis were obtained from ChemGenes. The unlabeled NHC phosphoramidite was prepared as previously reported.^10,25^ The synthesis of the N4 and N3 ^15^N-labeled NHC analogues is described in the Supporting Information. All RNA oligonucleotides were synthesized on CPG support (controlled pore glass) in 0.6 μM scale.^10^ Cleavage from the solid support and deprotection of the RNA oligonucleotides was achieved using MeNH_2_ in H_2_O/EtOH (1:1) for 6 h at 37 °C, followed by 1 M TBAF in THF for 12 h at 25 °C. RNA oligonucleotides were purified by denaturing polyacrylamide gel electrophoresis (PAGE, 20%) and analyzed by anion-exchange HPLC (Dionex DNAPac PA200) and HR-ESI-MS, as previously described.^10^

Thermal melting curves were recorded for five RNA duplex concentrations (1, 2, 5, 10, and 20 μM) in 10 mM sodium phosphate buffer (pH 7), containing 100 mM NaCl between 10 and 90 °C with a heating rate of 0.5 °C/min (two cooling and two heating ramps). Thermodynamic parameters were derived by van’t Hoff analysis (1/*T*_m_ vs ln *c*) as detailed in the Supporting Information.

NMR spectroscopy: After PAGE purification, the oligonucleotides used for NMR samples were precipitated with 5 volumes of LiClO_4_ in acetone (2% w/v) at −20 °C for 6 h. For 1D ^1^H NMR screening experiments of the Dickerson-Drew sequences 36 nmol of each sequence was dissolved in 180 μL of water/D_2_O (9:1) (final concentration of the duplex 100 μM). For 2D NMR and CLEANEX-PM experiments, the duplex concentration was 340–850 μM (details in Supporting Information). All the samples contained 3-(trimethylsilyl)-1-propanesulfonic acid (DSS) as internal NMR reference and NMR buffer (10 mM sodium phosphate at pH 7.0 containing 100 mM NaCl). Annealing was performed by heating the NMR samples for 2 min at 95 °C followed by slow cooling to room temperature.

All the NMR experiments were performed on a Bruker Avance III HD 600 NMR spectrometer equipped with a DCH ^13^C/^1^H cryoprobe or on a Bruker Avance III HD 600 NMR spectrometer equipped with a BBFO room-temperature probe. The spectra were acquired and processed with the software Topspin 3.2 (Bruker BioSpin, Germany) and analyzed using the software Sparky 3.114.^37^

The 1D ^1^H NMR screening experiments as well as the 2D ^1^H,^1^H NOESY spectra were recorded using either jump-return-echo (excitation maximum on the middle of the imino region)^38^ or excitation sculpting with gradients^39^ for water suppression. The NOESY mixing time was 200 ms. The sequences containing N4 or N3 atom-specifically ^15^N-labeled NHC were characterized using 1D ^1^H,^15^N filtered ^1^H experiments and 2D ^1^H,^15^N HSQC employing the soft WaterGATE^40^ water suppression scheme. Water hydrogen exchange rates of imino protons (*k*_EX_) were measured using a 1D version of the CLEANEX-PM pulse sequence available as Bruker standard experiment (zgcxesgp), according to the protocol described before^41,42^ and detailed in the Supporting Information.

## Supporting Information Available

Experimental Section with synthetic procedures and NMR spectra, mass spectrometry and thermal melting data (PDF)
