# Supplementary material for: The Tautomeric State of N4-Hydroxycytidine within Base-Paired RNA
Source: ACS Cent Sci. 2024 Apr 25;10(5):1084–93. doi: 10.1021/acscentsci.4c00146 (PMC11117714; doi:10.1021/acscentsci.4c00146)
Supplement: Supplementary file 1 — oc4c00146_si_001.pdf [file oc4c00146_si_001.pdf]

# Supporting Information

## for

# The tautomeric state of $N^4$-Hydroxycytidine within base-paired RNA

Irene Bessi[1], Carina Stiller[1], Till Schroeder[1], Benedikt Schäd[1], Julia Dietzsch[1], Matthias Grüne[1] and Claudia Höbartner[1,2]*

[1] Institute of Organic Chemistry, Julius-Maximilians-University Würzburg, Am Hubland, 97074 Würzburg, Germany

[2] Center for Nanosystems Chemistry, Julius-Maximilians-University Würzburg, 97074 Würzburg, Germany

**Table of Contents**

**Supporting Tables and Figures and Schemes**

**Table S1** ESI-MS data of the DD derived sequences ........ S1

**Table S2** $T_M$ of the DD derived sequences ........ S1

**Table S3** ESI-MS data of the different RdRp model substrates ........ S2

**Table S4** $T_M$ of the various RdRp model substrates ........ S2

**Figure S1** UV melting curves of DD, X3, X7, X8, X9, X11 at various RNA concentration ........ S3

**Figure S2** UV melting curves of CG, XG, UA, XA, UG, CA at various RNA concentration ........ S4

**Figure S3** Imino region of the 1D $^1$H NMR spectrum of the DD variants at various temperatures.. S5-6

**Figure S4** Assignment of the imino region of the 2D $^1$H,$^1$H-NOESY spectra ........ S7

**Figure S5** Assignment of the aromatic-anomeric region of the 2D $^1$H,$^1$H-NOESY spectra ........ S8

**Figure S6** Adenine C2-H2 region of the $^1$H,$^{13}$C-HSQC of XA and CA ........ S9

**Figure S7** 2D $^1$H,$^{15}$N-HSQC and $^1$H,$^{15}$N-HMBC spectra of compound 1 and 3 ........ S10

**Figure S8** Assignment of the aromatic region of the 2D $^1$H,$^1$H-NOESY spectrum of XA ........ S11

**Figure S9** Assignment of imino/aromatic-amino region of the 2D $^1$H,$^1$H-NOESY spectrum of XA ... S12

**Figure S10** CSP analysis of the conformations “a” and “b” of XA respect to UA ........ S13

**Figure S11** Imino-water exchange rates derived from 1D CLEANEX-PM experiments ........ S14

**Figure S12** UV melting curves of the RdRp substrate with 11 bp and 4 nt overhang ........ S15

**Figure S13** UV melting curves of the fully elongated RdRp substrate with 15 bp (mod. 5/11) ........ S16

**Figure S14** UV melting curves of the fully elongated RdRp substrate with 15 bp (mod. 13/3) ........ S17

**Scheme S1** Synthesis of $^{15}$N(4)-labeled NHC phosphoramdite ........ S18

**Scheme S2** Synthesis of $^{15}$N(3)-labeled Uridine and NHC-phosphoramidite ........ S19

**Experimental procedures**

General information ........ S20

Synthetic procedures and NMR spectra for compounds 1-9 ........ S21

Native gel electrophoresis ........ S53

UV melting curves fitting and Van’t Hoff analysis ........ S53

NMR samples details ........ S54

NMR CLEANEX-PM ........ S54

References ........ S56

**Table S1** ESI-MS results of the Dickerson-Drew derived sequences with NHC (X) at different positions. Modifications compared to the original Dickerson-Drew dodecamer (DD) are underlined in the sequence. NHC containing $^{15}$N label at N4 or N3 is red or blue, respectively.

| Name (Main) | Bp at mod. site | Name (SI) | 5'-sequence-3' | nt | Chemical formula | Calculated mass | Measured mass |
|---|---|---|---|---|---|---|---|
| **DD** | | R922 | CGCGAAUUCGCG | 12 | $C_{114}H_{143}N_{46}O_{82}P_{11}$ | 3808.55476 | 3808.55557 |
| **U3** | UG | R976 | CGUGAAUUCGCG | 12 | $C_{114}H_{142}N_{45}O_{83}P_{11}$ | 3809.53878 | 3809.52670 |
| **X3** | XG | R961 | CGXGAAUUCGCG | 12 | $C_{114}H_{143}N_{46}O_{83}P_{11}$ | 3824.54968 | 3824.53865 |
| **X7** | XA | R962 | CGCGAAXUCGCG | 12 | $C_{114}H_{144}N_{47}NO_{82}P_{11}$ | 3823.56566 | 3823.58965 |
| **X8** | XA | R911 | CGCGAAUXCGCG | 12 | $C_{114}H_{144}N_{47}NO_{82}P_{11}$ | 3823.56621 | 3823.56799 |
| **X9** | XG | R910 | CGCGAAUUXGCG | 12 | $C_{114}H_{143}N_{46}O_{83}P_{11}$ | 3824.55022 | 3824.54427 |
| **X11** | XG | R963 | CGUGAAUUCGXG | 12 | $C_{114}H_{143}N_{46}O_{83}P_{11}$ | 3824.54968 | 3824.54806 |
| **X3** | XG | R993 | CGXGAAUUCGCG | 12 | $C_{114}H_{143}N_{45}{}^{15}NO_{83}P_{11}$ | 3825.55695 | 3825.54671 |
| **X7** | XA | R994 | CGCGAAXUCGCG | 12 | $C_{114}H_{144}N_{46}{}^{15}NO_{82}P_{11}$ | 3824.54891 | 3824.56269 |
| **U3A10** | UA | R1006 | CGUGAAUUCACG | 12 | $C_{114}H_{142}N_{45}O_{82}P_{11}$ | 3793.54386 | 3793.54112 |
| **X3A10** | XA | R1007 | CGXGAAUUCACG | 12 | $C_{114}H_{143}N_{45}{}^{15}NO_{82}P_{11}$ | 3809.55180 | 3809.54283 |
| **X3A10** | XA | R1071 | CGXGAAUUCACG | 12 | $C_{114}H_{143}N_{45}{}^{15}NO_{82}P_{11}$ | 3809.56259 | 3809.54264 |
| **C3A10** | CA | R1130 | CGCGAAUUCACG | 12 | $C_{114}H_{143}N_{46}O_{81}P_{11}$ | 3792.56039 | 3792.54480 |

**Table S2** Melting temperature ($T_M$) in °C of the Dickerson-Drew derived sequences with NHC (X) at different positions. NHC containing $^{15}$N label at N4 is red. $T_M$ derived by sigmoidal fitting of UV melting curves at different RNA duplex concentration (indicated in brackets). *$T_M$ derived by derivation of the UV melting curve.

| Name | bp at mod. site | Name (SI) | 5'-sequence-3' | $T_M$ [20 µM] | $T_M$ [10 µM] | $T_M$ [5 µM] | $T_M$ [2 µM] | $T_M$ [1 µM] |
|---|---|---|---|---|---|---|---|---|
| **DD** | | R922 | CGCGAAUUCGCG | 67.2 | 65.3 | 63.4 | 60.7 | 59.0 |
| **U3** | UG | R976 | CGUGAAUUCGCG | 49.0 | 47.9 | 46.3 | 44.4 | 43.1 |
| **X3** | XG | R961 | CGXGAAUUCGCG | 51.1 | 49.4 | 48.1 | 46.6 | 45.5 |
| **U3A10** | UA | R1006 | CGUGAAUUCACG | 59.1 | 57.6 | 56.5 | 54.1 | 52.7 |
| **X3A10** | XA | R1007 | CGXGAAUUCACG | 43.8 | 42.0 | 40.6 | 38.7 | 37.0 |
| **C3A10** | CA | R1130 | CGCGAAUUCACG | 45.7 | 43.9 | 42.5 | 40.5 | 39.1 |
| **X7** | XA | R962 | CGCGAAXUCGCG | 61.7* | | | 39.9/64.5* | |
| **X8** | XA | R911 | CGCGAAUXCGCG | 62.4* | | | 38.7/63.8* | |
| **X9** | XG | R910 | CGCGAAUUXGCG | 47.6* | | | 35.7/55.3* | |
| **X11** | XG | R963 | CGCGAAUUCGXG | 51.9 | 50.3 | 48.7 | 47.0 | 45.7 |

**Table S3** ESI-MS results of the different RdRp model substrates with NHC (**X**) at different positions.

| Name | 5'-sequence-3' | nt | formula | Mass calc. | Mass found |
|---|---|---|---|---|---|
| **R840** | CACUGCGUAGGCUCA | 15 | $C_{142}H_{178}N_{56}O_{103}P_{14}$ | 4748.67387 | 4748.67166 |
| **R841** | UGAGCCUACGCAGUG | 15 | $C_{143}H_{178}N_{58}O_{103}P_{14}$ | 4788.68002 | 4788.66430 |
| **R880** | UGAGCCUACGCAAUG | 15 | $C_{143}H_{178}N_{58}O_{102}P_{14}$ | 4772.68510 | 4772.66377 |
| **R892** | CA**X**UGCGUAGGCUCA | 15 | $C_{142}H_{178}N_{56}O_{104}P_{14}$ | 4764.66878 | 4764.67966 |
| **R894** | UGAG**X**CUACGCAGUG | 15 | $C_{143}H_{178}N_{58}O_{104}P_{14}$ | 4804.67493 | 4804.66837 |
| **R917** | CACUGCGUAGG | 11 | $C_{105}H_{131}N_{43}O_{75}P_{10}$ | 3503.51347 | 3503.51600 |
| **R918** | CACUGCGUAGA | 11 | $C_{105}H_{131}N_{43}O_{74}P_{10}$ | 3487.51856 | 3487.51528 |
| **R921** | CACUGCGUAGACUCA | 15 | $C_{142}H_{178}N_{56}O_{102}P_{14}$ | 4732.67895 | 4732.67155 |
| **R945** | UGAGUCUACGCAGUG | 15 | $C_{143}H_{177}N_{57}O_{104}P_{14}$ | 4789.66403 | 4789.65330 |
| **R947** | CAUUGCGUAGGCUCA | 15 | $C_{142}H_{177}N_{55}O_{104}P_{14}$ | 4749.65843 | 4749.65855 |

**Table S4** Melting temperature ($T_M$) in °C of the different RdRp model substrates with NHC (X) at different positions. $T_M$ derived by sigmoidal fitting of UV melting curves at different RNA duplex concentration (indicated in brackets).

| **Sequence (bp at modification site)** | | $T_M$ [20 µM] | $T_M$ [10 µM] | $T_M$ [5 µM] | $T_M$ [2 µM] | $T_M$ [1 µM] |
|---|---|---|---|---|---|---|
| R841/R917 (CG) | | 64.7 | 63.3 | 62.1 | 60.3 | 59.1 |
| R894/R917 (XG) | 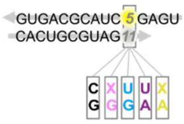  | 61.4 | 59.9 | 58.6 | 57.0 | 55.5 |
| R945/R918 (UA) | | 60.9 | 59.3 | 57.8 | 56.0 | 54.4 |
| R894/R918 (XA) | | 60.8 | 59.1 | 58.1 | 56.4 | 55.0 |
| R945/R917 (UG) | | 60.3 | 58.5 | 57.1 | 55.2 | 53.5 |
| R841/R840 (CG) | | 77.6 | 75.9 | 75.0 | 74.1 | 73.5 |
| R894/R840 (XG) | 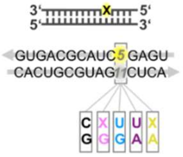  | 71.2 | 70.1 | 69.0 | 68.0 | 66.9 |
| R945/R921 (UA) | | 72.3 | 71.0 | - | 69.2 | 67.7 |
| R894/R921 (XA) | | 68.2 | 67.3 | 66.5 | 65.2 | 64.4 |
| R945/R840 (UG) | | 70.5 | 69.3 | 68.0 | 66.5 | 65.8 |
| R840/R841 (CG) | | 77.6 | 75.9 | 75.0 | 74.1 | 73.5 |
| R892/R841 (XG) | 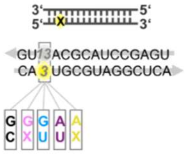  | 73.3 | 71.8 | 70.4 | 69.2 | 68.5 |
| R947/R880 (UA) | | 73.9 | 72.4 | 71.2 | 69.5 | 68.2 |
| R892/R880 (XA) | | 73.8 | 72.4 | 70.7 | 69.4 | 67.4 |
| R947/R841 (UG) | | 72.4 | 70.1 | 69.0 | 67.4 | 66.2 |

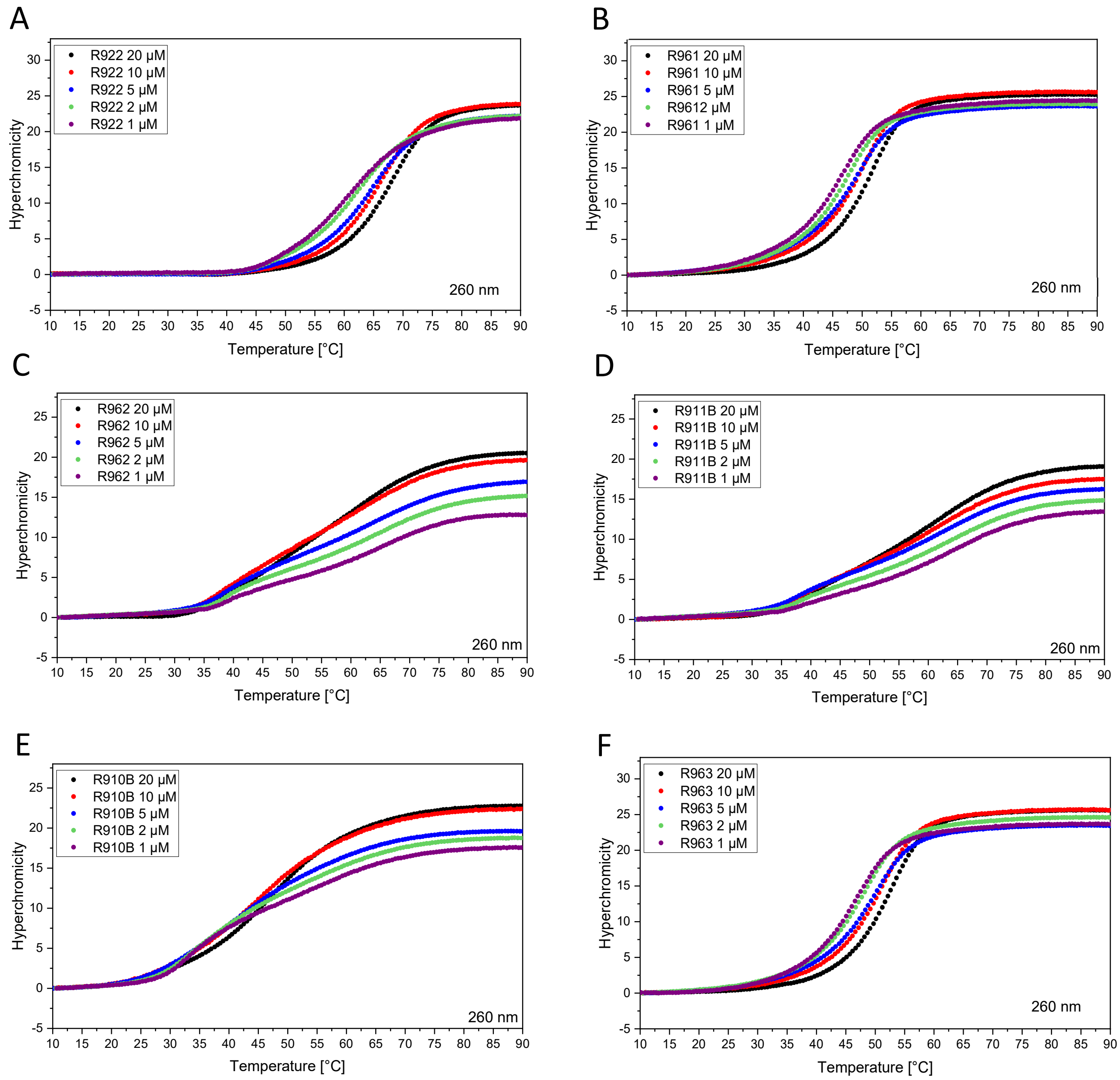


**Figure S1** UV melting curves of the Dickerson-Drew Sequences R922 (DD, A), R961 (X3, B), R962 (X7, C), R911 (X8, D), R910 (X9, E), R963 (X11, F) at 1, 2, 5, 10 and 20 µM duplex RNA. Hyperchromicity at 260 nm.

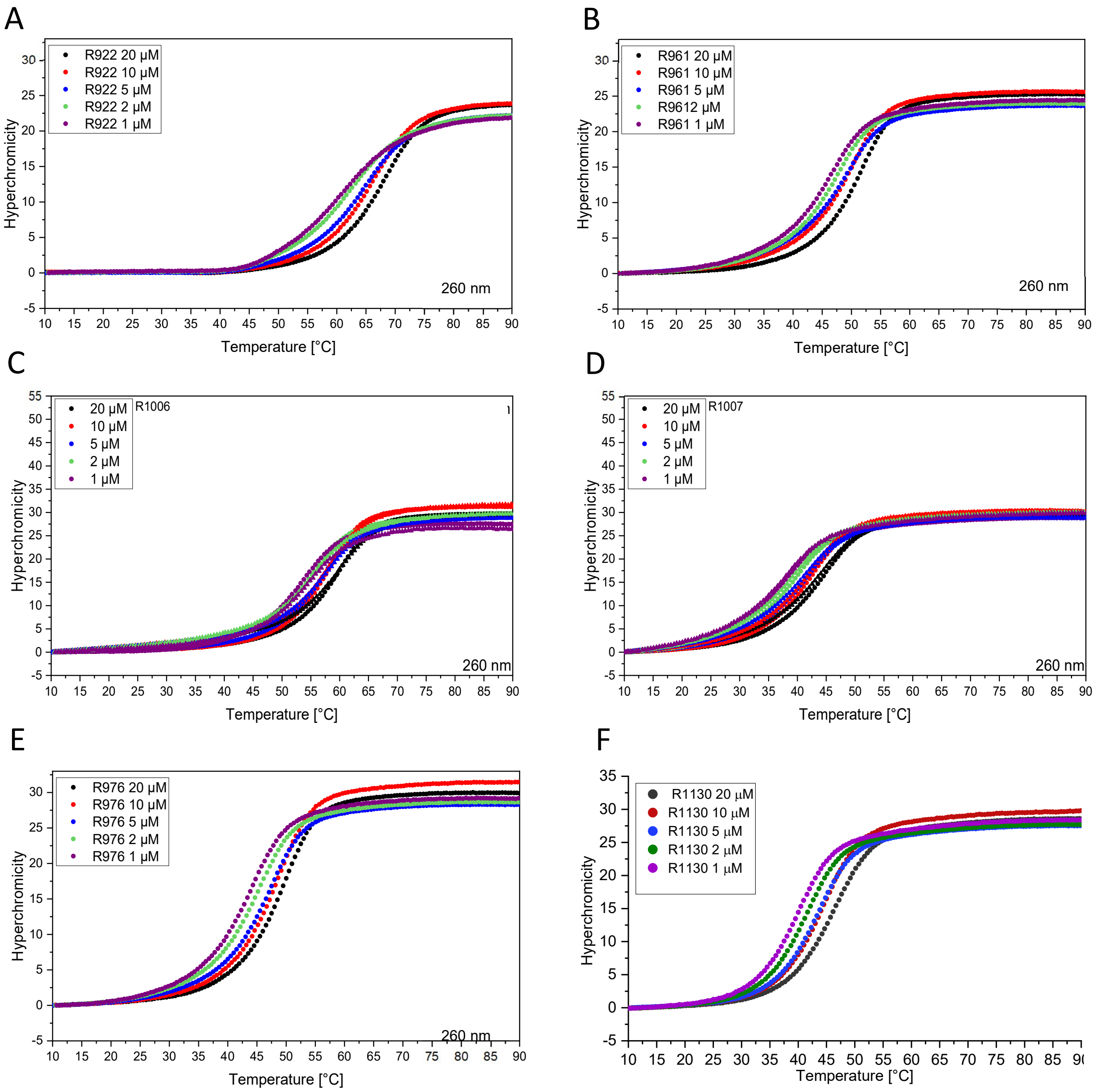


**Figure S2** UV melting curves of the Dickerson-Drew Sequences R922 (CG, A), R961 (XG, B), R1006 (UA, C), R1007 (XA, D), R976 (UG, E), R1130 (CA, F) at 1, 2, 5, 10 and 20 µM duplex RNA. Hyperchromicity at 260 nm.

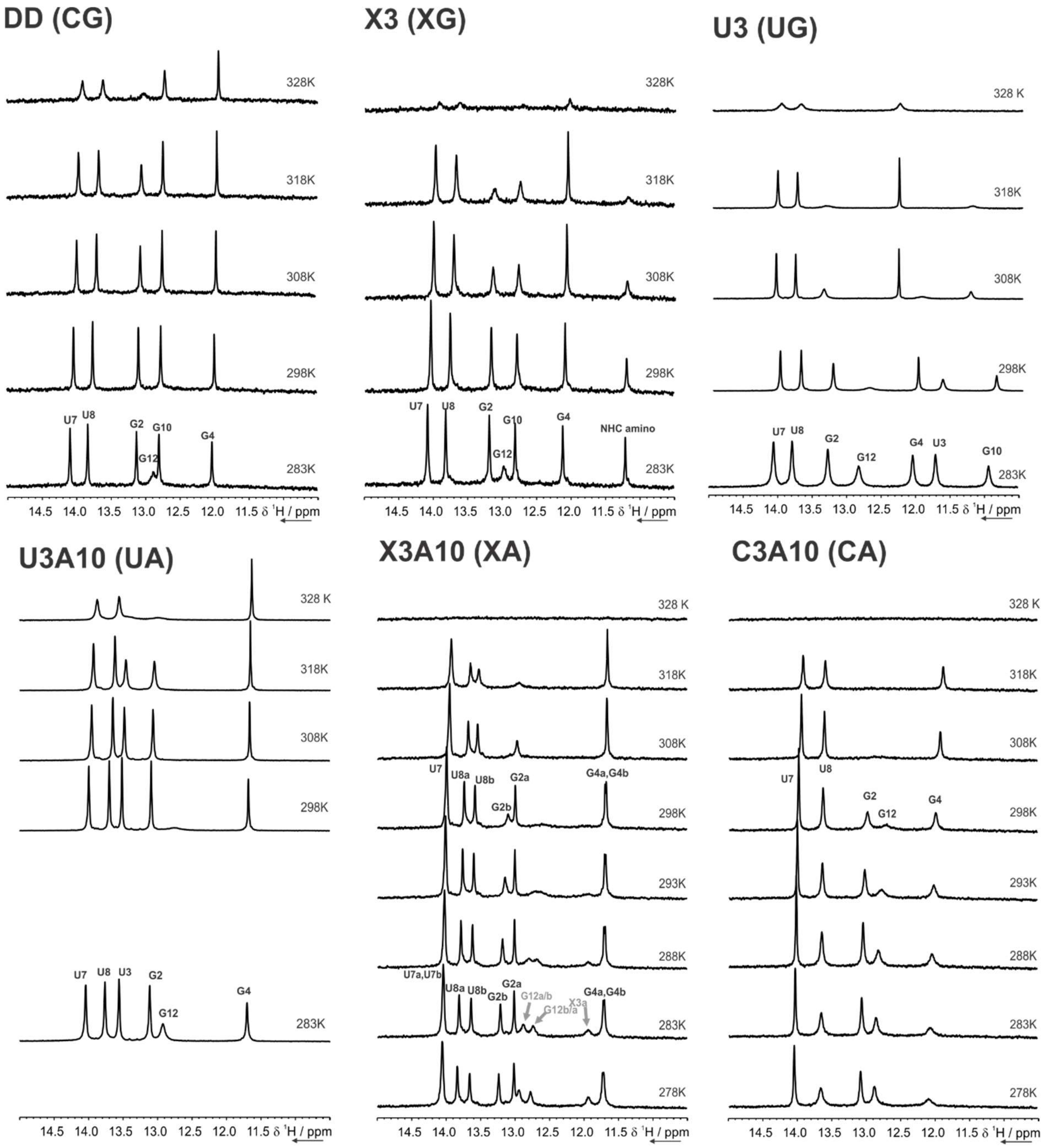

DD (CG)
X3 (XG)
U3 (UG)
U3A10 (UA)
X3A10 (XA)
C3A10 (CA)
328K
318K
308K
298K
293K
288K
283K
278K
U7
U8
U3
G2
G10
G12
G4
NHC amino
U8a
U8b
G2a
G2b
G4a,G4b
U7a,U7b
G12a/b
G12b/a
X3a
14.5 14.0 13.5 13.0 12.5 12.0 11.5 δ ¹H / ppm

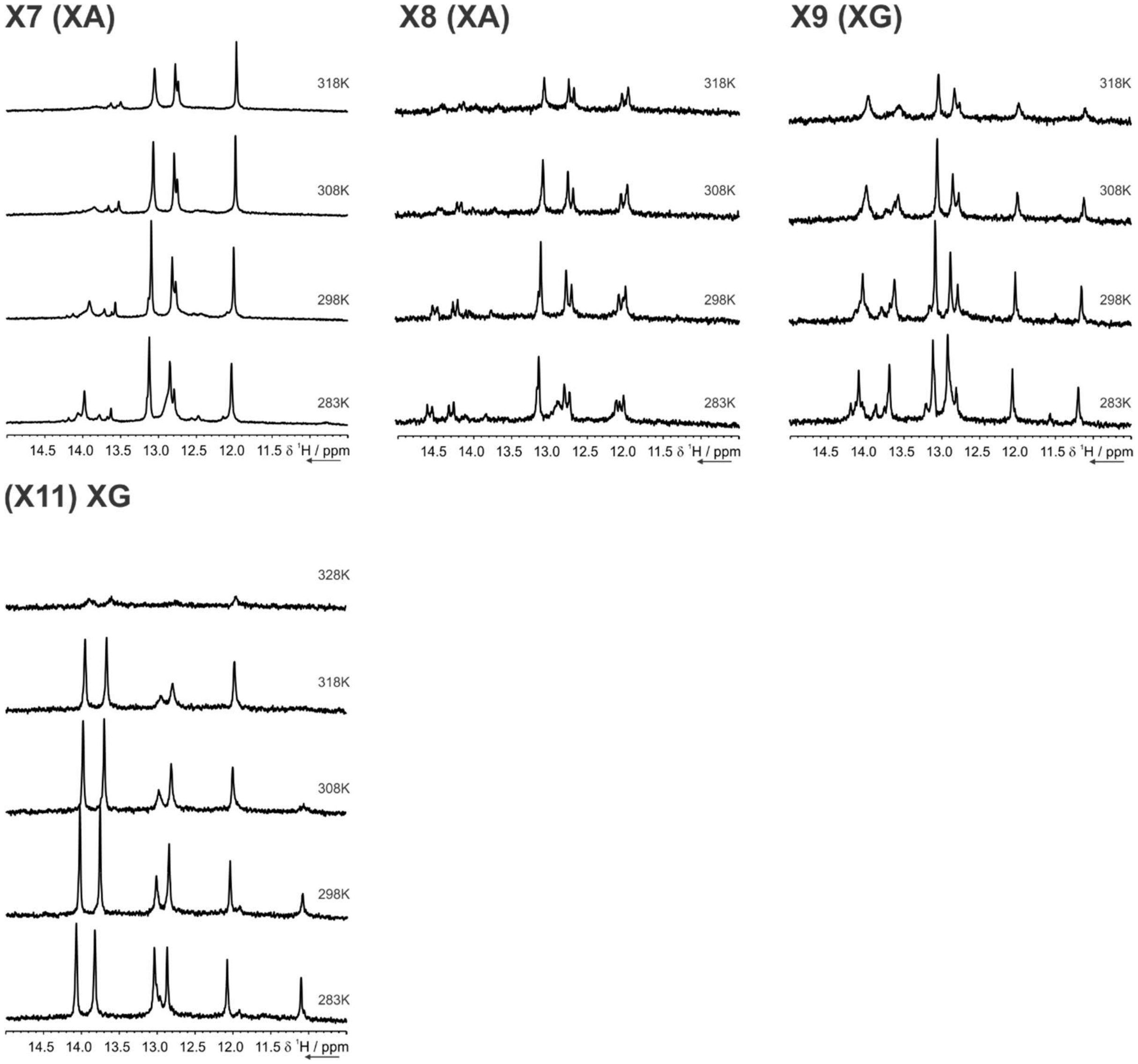


**Figure S3** Imino region of the 1D $^{1}$H NMR spectrum of the Dickerson-Drew variants from Table S2 (base pair at modification site in brackets) at various temperatures. Experimental conditions: 10% $D_2O$/90% $H_2O$, NMR buffer, 600 MHz.
XA: Recorded on R1007 containing $^{15}$N(4)-labeled NHC.
XG: Recorded on R961 containing NHC at natural abundance.

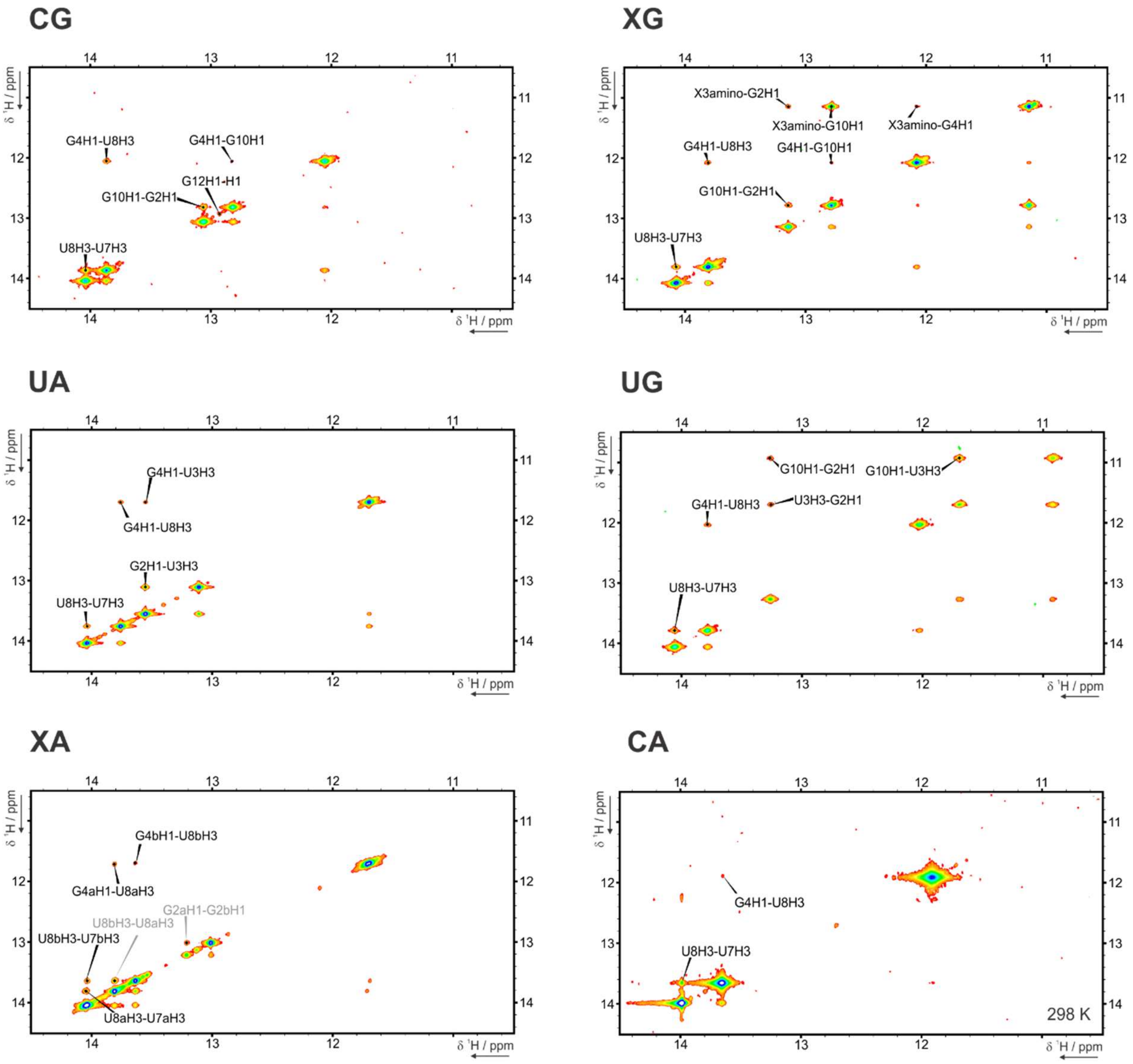


**Figure S4** Imino region of the 2D ¹H,¹H-NOESY spectrum of the Dickerson-Drew duplexes with assignment. Experimental conditions: 10% $D_2O$/90% $H_2O$, NMR buffer, 600 MHz. Spectra recorded at 283 K unless otherwise specified.

XA: Recorded on R1007 containing $^{15}N(4)$-labeled NHC. Exchange peaks between conformation "a" and conformation "b" are labeled in gray.

XG: Recorded on R961 containing NHC at natural abundance.

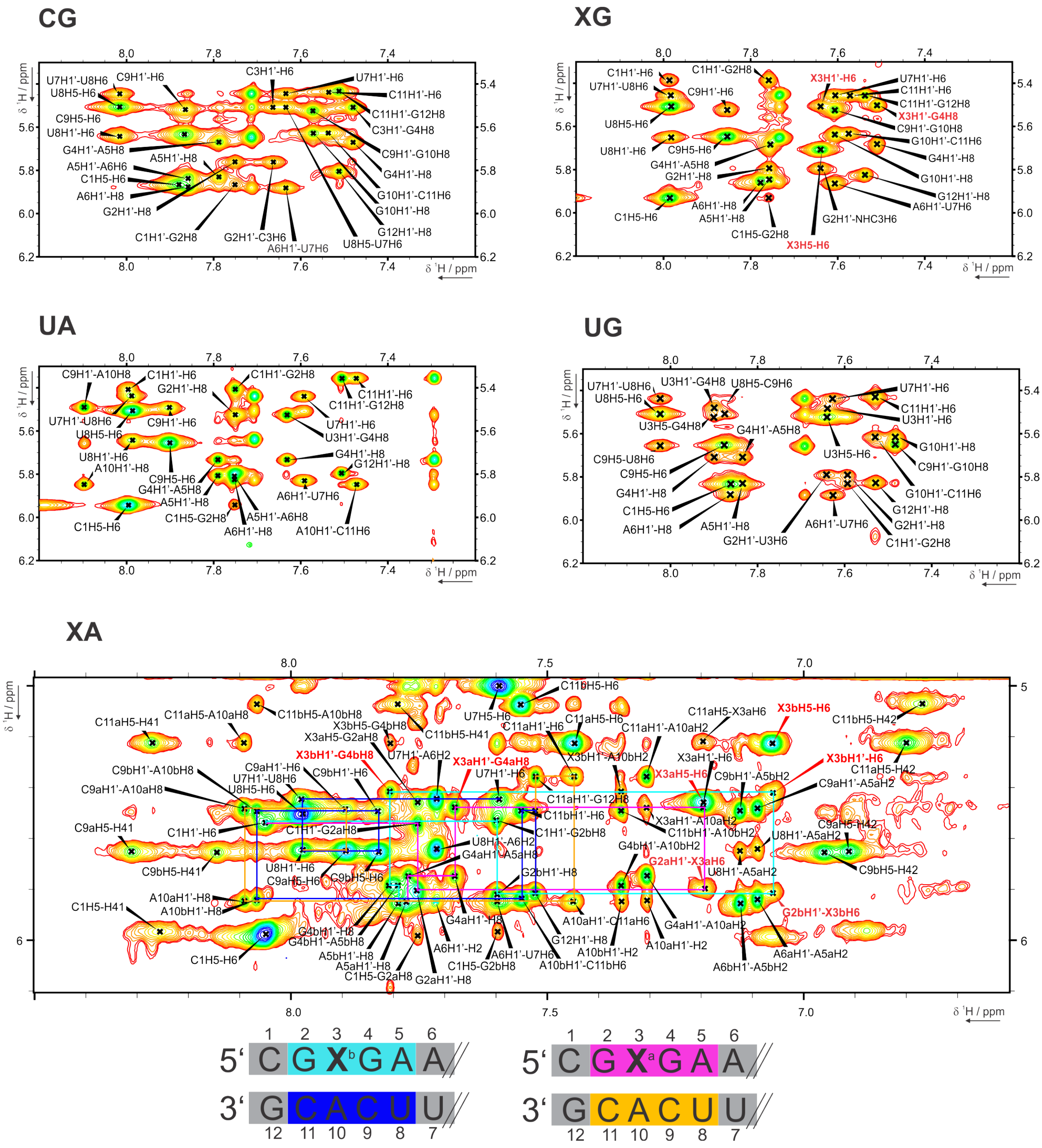


**Figure S5** Aromatic-anomeric region of the 2D $^1H,^1H$-NOESY spectrum of the Dickerson-Drew duplexes with assignment. Experimental conditions: 10% $D_2O$/90% $H_2O$, NMR buffer, 283 K, 600 MHz. XA: Recorded on R1007 containing $^{15}N(4)$-labeled NHC. The terminal base pair C1-G12 and the most inner base pair A6-U7 seem to be almost equivalent in the two conformations and only one set of peaks is detected (gray background in the scheme).

The tracts G2-A5 and U8-C11 show two sets of peaks, pointing at the presence of two different conformations. Indeed, two different aromatic-anomeric paths are detected for conformation "b" (light blue G2-A5 and dark blue U8-C11 in the scheme) and conformation "a" (pink G2-A5 and yellow U8-C11 in the scheme).

XG: Recorded on R961 containing NHC at natural abundance.

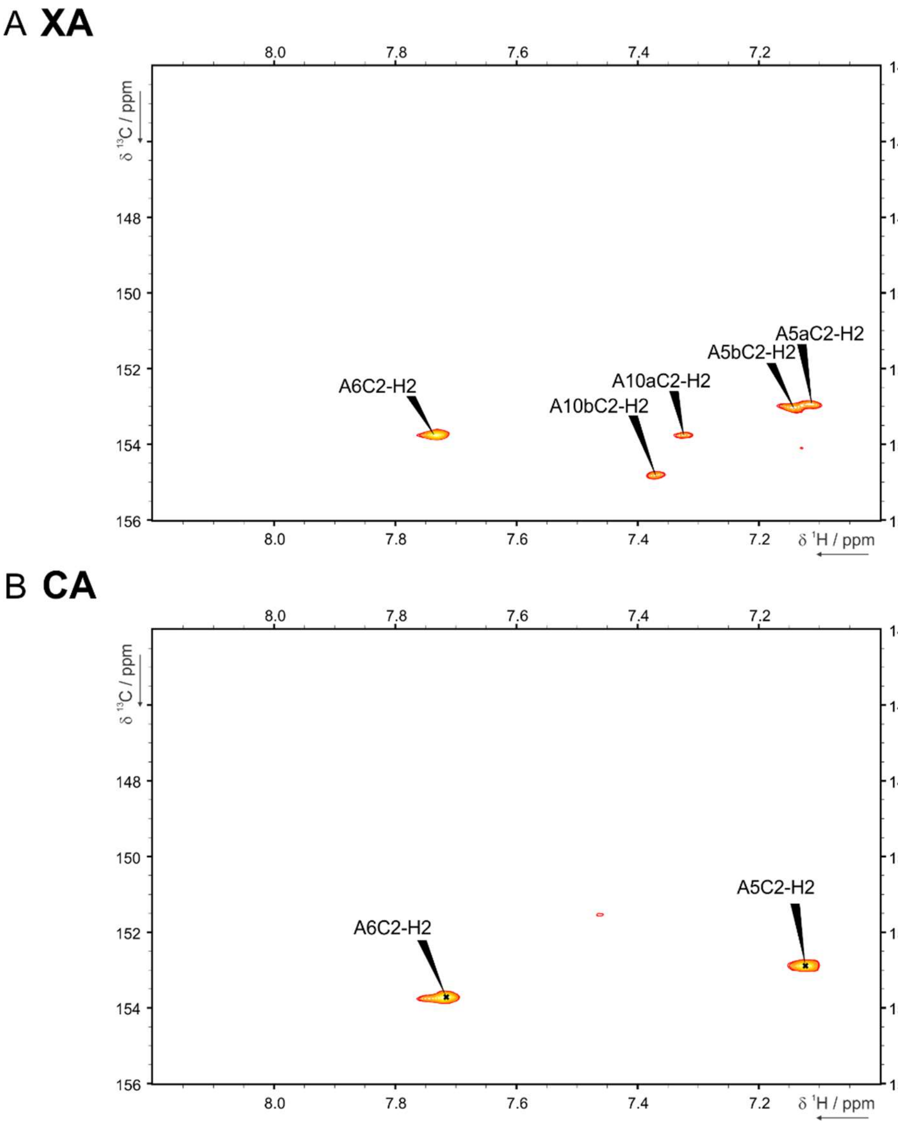


**Figure S6** Adenine C2-H2 region of the $^{1}$H,$^{13}$C-HSQC of Dickerson-Drew duplexes XA (A) and CA (B) with assignment. The $^{13}$C chemical shift of adenine C2 is a reporter signal for adenine N1 protonation and can be upfield-shifted up to 6 ppm when adenine is protonated at N1.[1],[2],[3]

The $^{13}$C HSQC of XA (A) shows that A10H2a and A10H2b are correlated with carbon C2 resonating at 153.7 and 154.8 ppm, respectively. This excludes an NHC:(A10$^{+}$) base pair with A10 protonated at N1.

In the $^{1}$H,$^{13}$C-HSQC of CA (B) the signal of A10 C2-H2 is broadened beyond detection. Furthermore, the signal of imino protons of residues U8 and G4 are also broadened (Figure 3 and discussion in the main article). This might be due to dynamics in the fast/intermediate regime on the NMR time scale. As previously reported,[2] the apparent p$K_a$ of Adenine N1 in a CA base pair can be as high as 6.5, therefore, under our experimental conditions (pH 7.0), a small fraction of N1-protonated A could be in equilibrium with unprotonated A in the fast exchange regime on the NMR time scale. However, our data do not allow us to determine whether the broadening is due to a fast dynamic protonation/deprotonation equilibrium at A10 N1 or a to a fast equilibrium between different conformations.

Experimental conditions: 10% $D_2O$/90% $H_2O$, NMR buffer, 298 K, 600 MHz.

XA: Recorded on R1071 containing $^{15}$N(3)-labeled NHC.

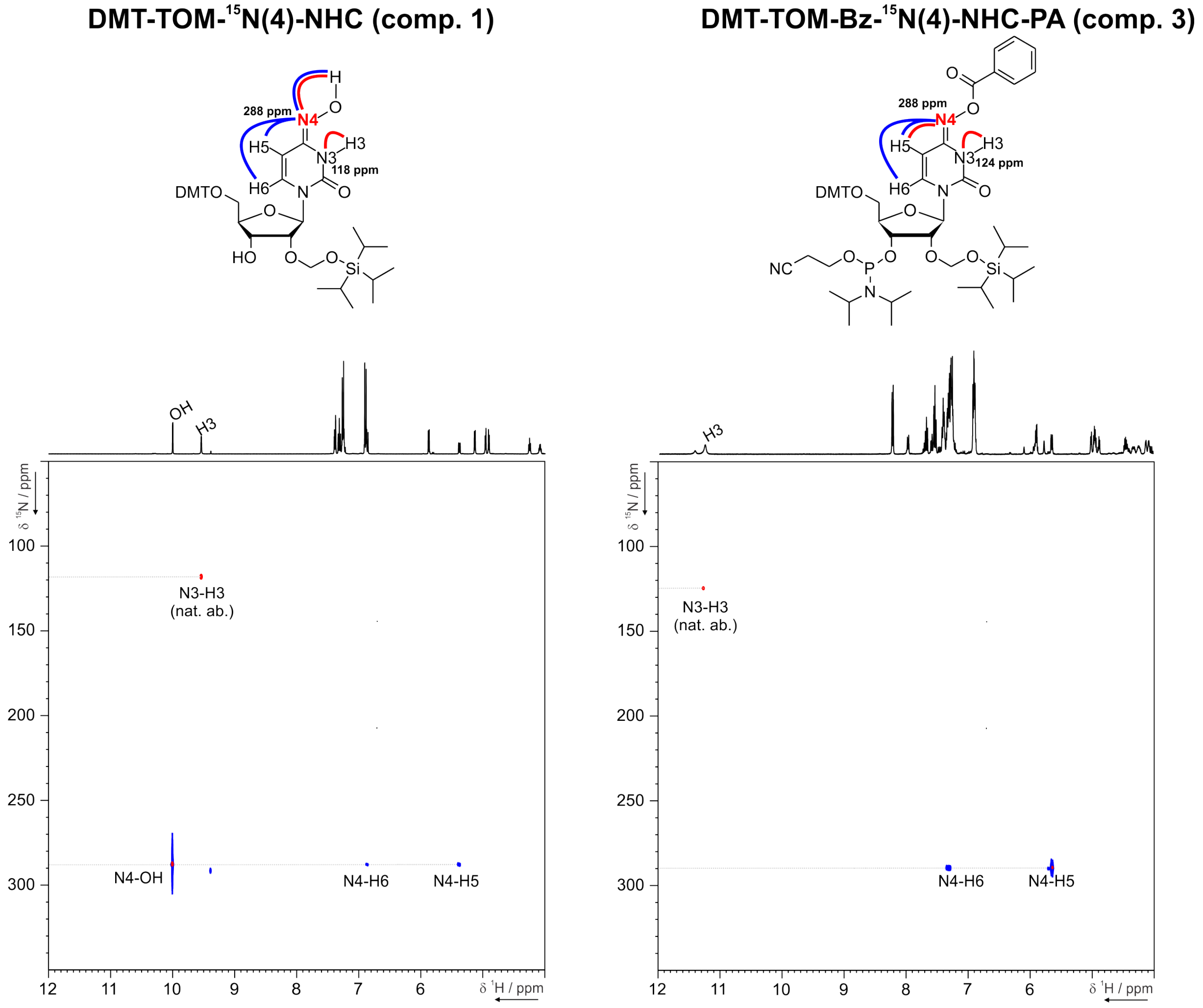


**Figure S7** Overlay of 2D $^{1}H,^{15}N$-HSQC (red) and 2D $^{1}H,^{15}N$-HMBC (blue) of compound **1** (left) and **3** (right) in 100% $d_6$-DMSO, 400 MHz, room temperature. Correlations detected in the spectra are marked on the respective structure with the corresponding color code. Only the imino form is observed. Long-range correlations N4-OH in compound **1** (left) and N4-H5 in compound **3** (right) are detected in the 2D $^{1}H,^{15}N$-HSQC due to the $^{15}N$ isotopic enrichment at N4.

**XA**

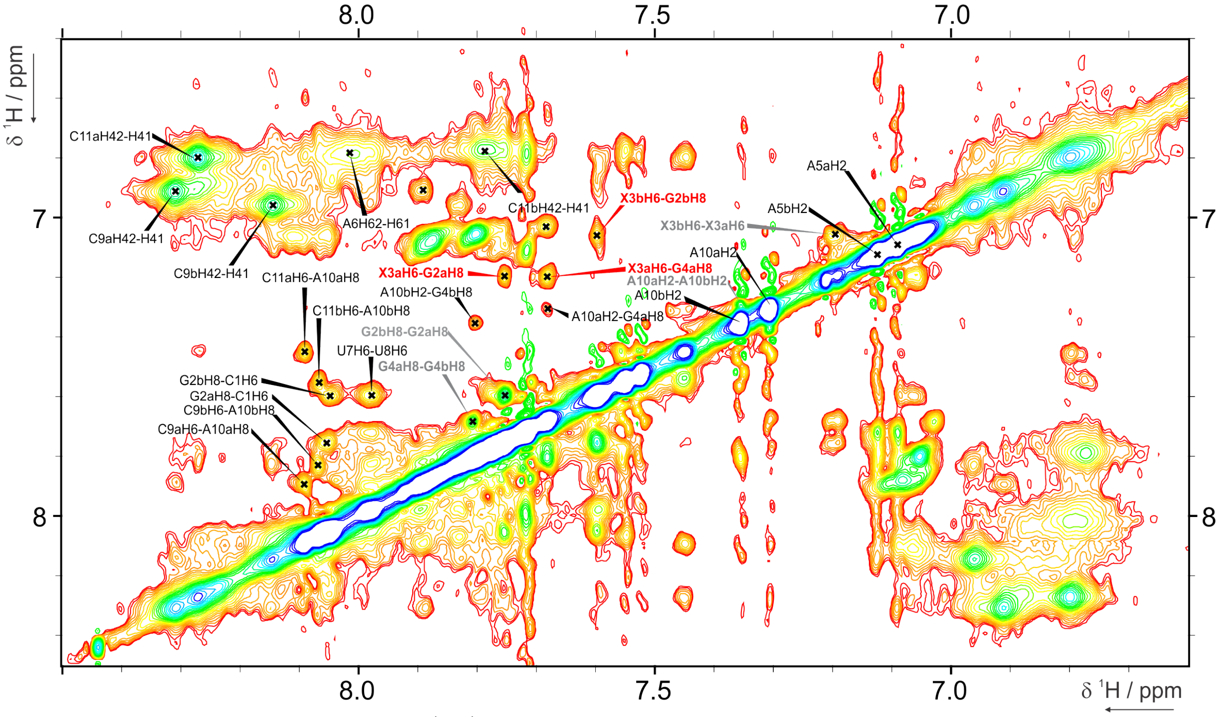


**Figure S8** Aromatic region of the 2D $^1$H,$^1$H-NOESY spectrum of the XA Dickerson-Drew variant (R1007, $^{15}$N(4)-labeled NHC) with assignment. Experimental conditions: 10% $D_2O$/90% $H_2O$, NMR buffer, 283 K, 600 MHz.
Exchange peaks between conformation “a” and conformation “b” are labeled in gray.

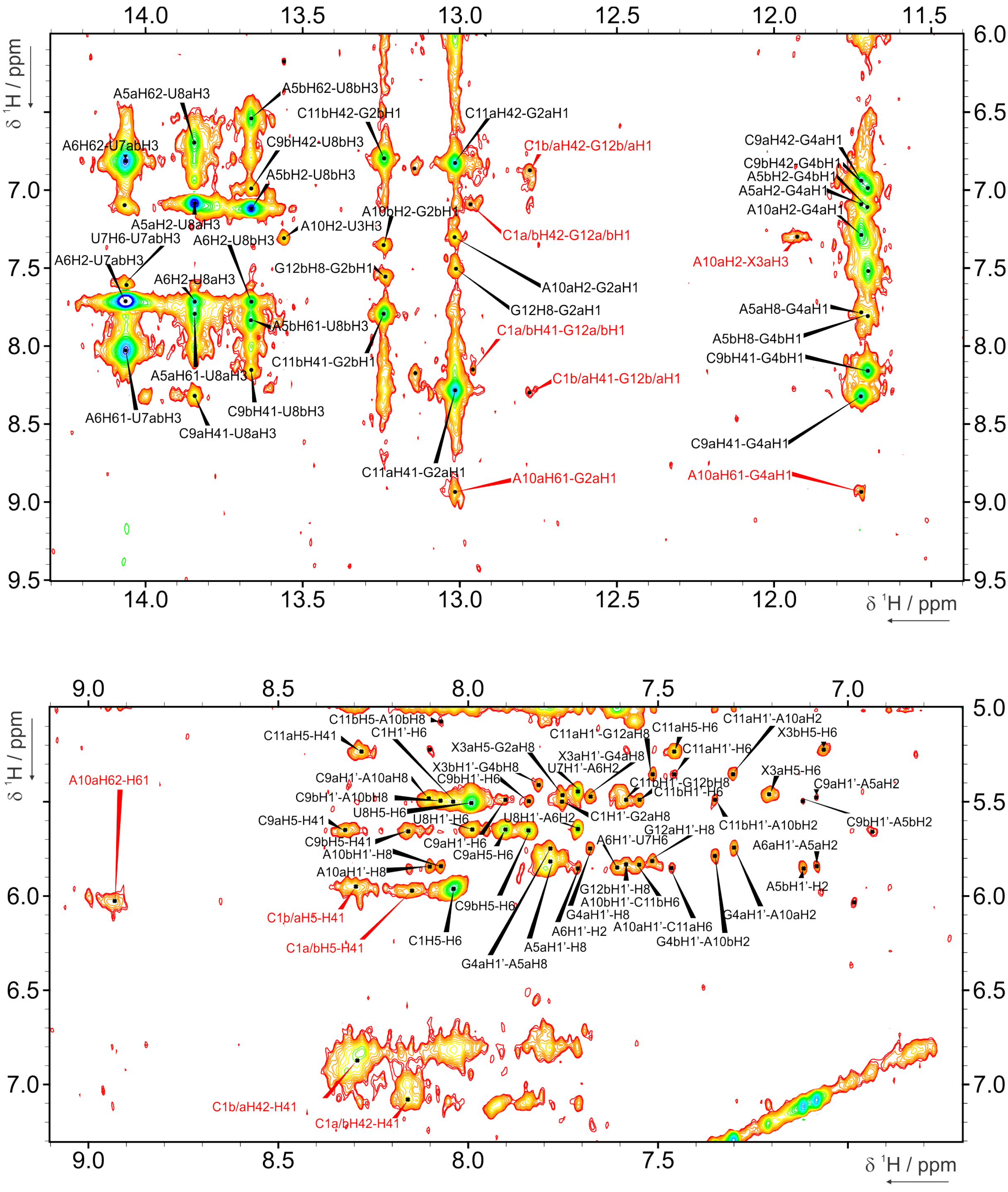


**Figure S9** Imino-amino (top) and aromatic-amino/anomeric (bottom) region of the 2D $^{1}H$,$^{1}H$-NOESY spectrum of the XA Dickerson-Drew variant (R1007, $^{15}N(4)$-labeled NHC) at 277 K.
Experimental conditions: 10% $D_2O$/90% $H_2O$, NMR buffer, 600 MHz.
Red labeled highlight cross peaks crucial for the assignment of G12a/b and G12b/a and X3aH3 imino protons. Assignment of the broad peaks at 12.75 and 12.9 ppm is based on the presence of cross peaks to C1 amino protons (top spectrum). Assignment of X3aH3 is based on the presence of a cross peak to A10aH2.

A **Chemical shift perturbation (CSP) analysis**

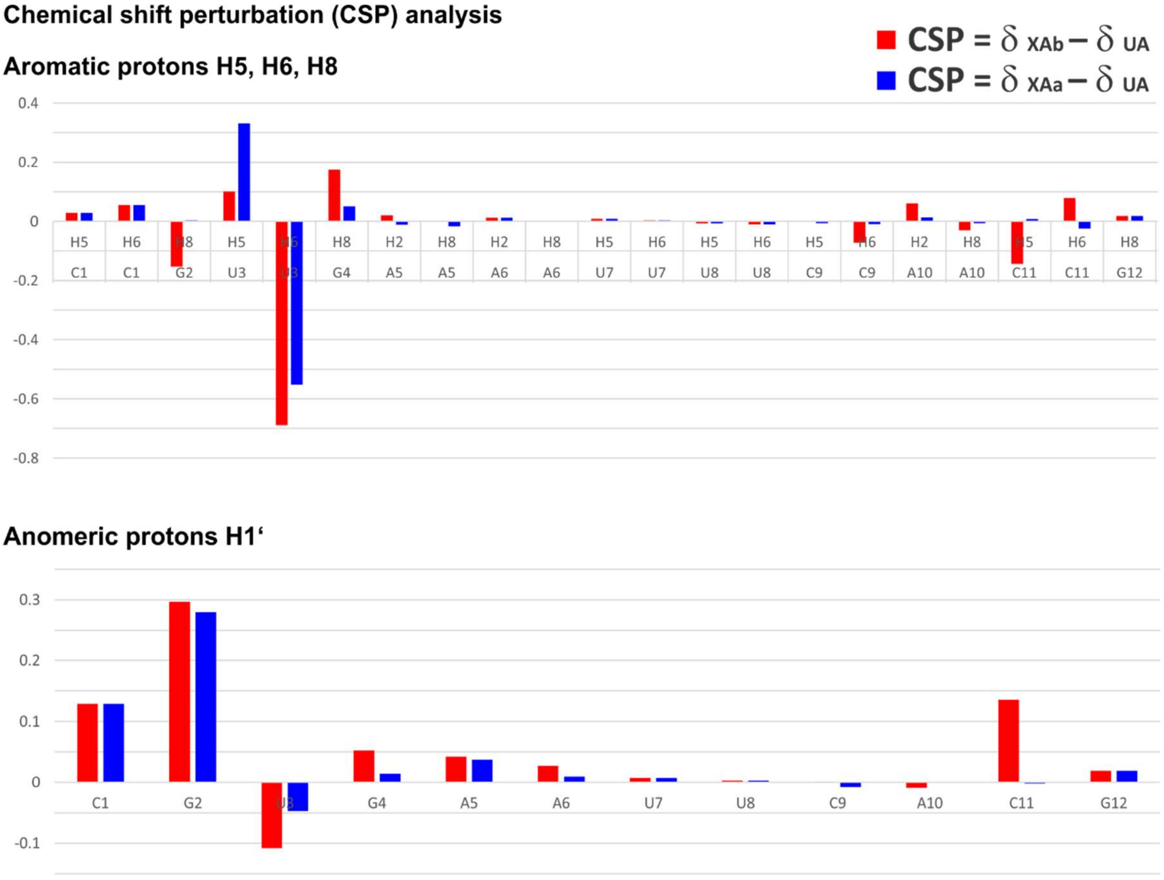


B **TOCSY CSP**

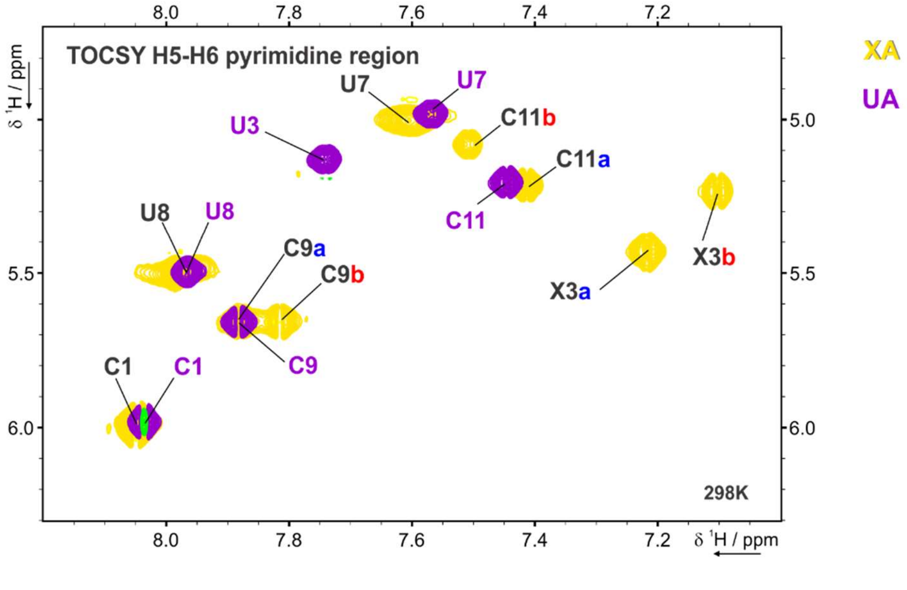


**Figure S10** Chemical shift perturbation (CSP) analysis of the two conformations "a" (blue) and "b" (red) of the XA Dickerson-Drew variant (R1007, $^{15}N(4)$-labeled NHC), respect to the UA Dickerson-Drew variant (R1006). A) Analysis performed using the c.s. of aromatic and anomeric protons extracted from the 2D $^{1}H,^{1}H$-NOESY spectrum recorded at 283 K, 600 MHz, in 10% $D_2O$/90% $H_2O$, NMR buffer (Figure S5). B) Overlay of the H5-H6 region of the 2D $^{1}H,^{1}H$-TOCSY (30 ms mixing time) of UA (purple) and XA (yellow) with assignment (experimental conditions: 10% $D_2O$/90% $H_2O$, NMR buffer, 298 K, 600 MHz). C9a and C11a H5-H6 peaks in XA are only slightly perturbed compared to UA.

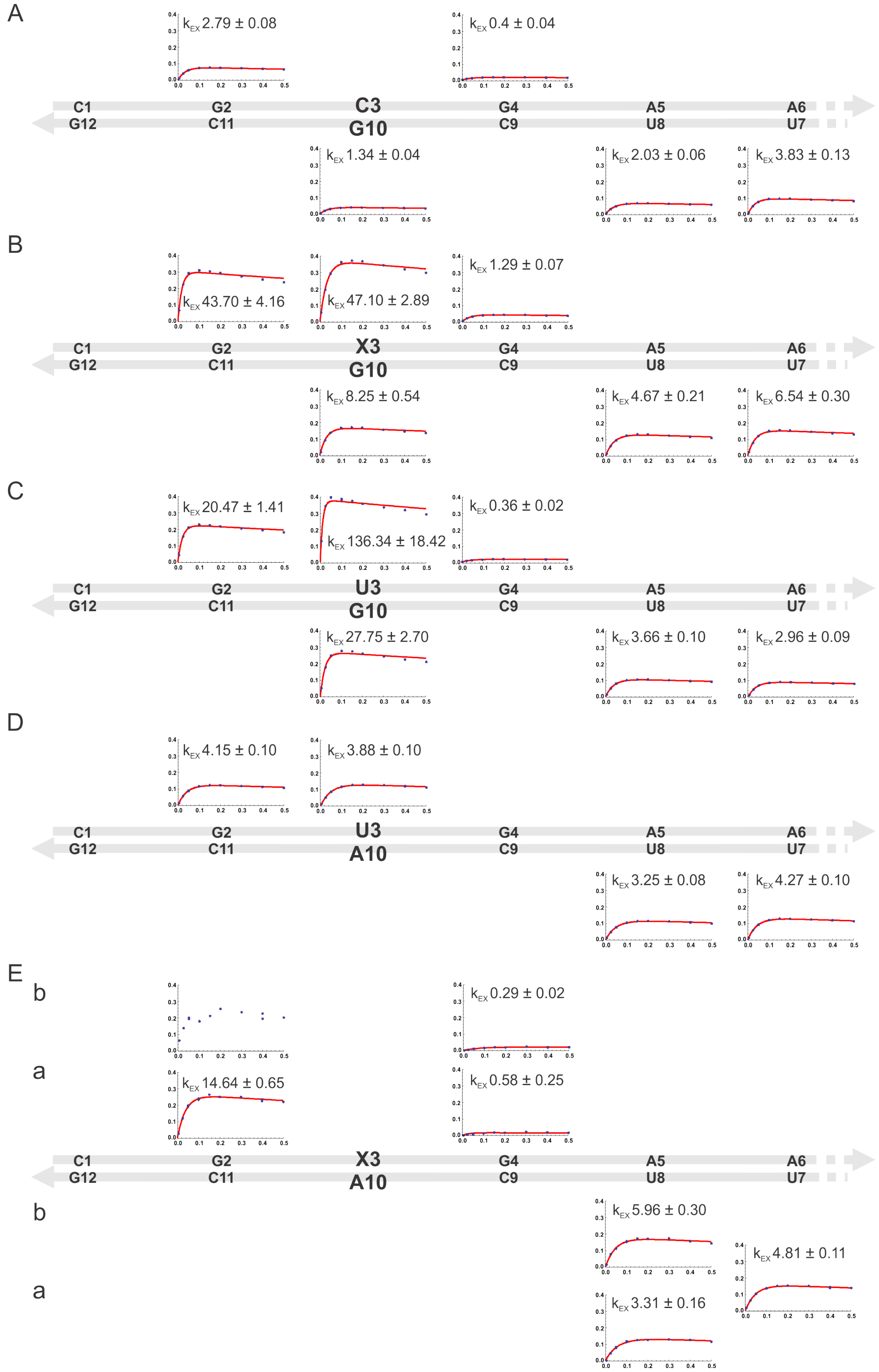


**Figure S11** Imino-water exchange rates ($k_{EX}$) derived from 1D CLEANEX-PM experiments for Dickerson-Drew sequences CG (A), XG (R961, B), UG (C), UA (D) and XA (R1007, E). Each graph shows the relative peak intensity of a specific imino proton NMR signal as a function of the mixing time (s) after water inversion in the CLEANEX-PM experiments. Plots are arranged along the duplex sequence, as a function of the imino-containing residue position along the duplex. $k_{EX}$ of G12 (in all the duplexes) and X3H3 in duplex XA (E) could not be determined due to peak broadening beyond detection at 298 K. $k_{EX}$ of G2b in duplex XA could not be determined due to poor quality of the data. The $k_{EX}$ of X3 in duplex XG (B) refers to the amino proton X3H4. The best fit to Eq. 2 is depicted as a solid red line. Results of the fitting are reported on top of each graph (error from the fitting).

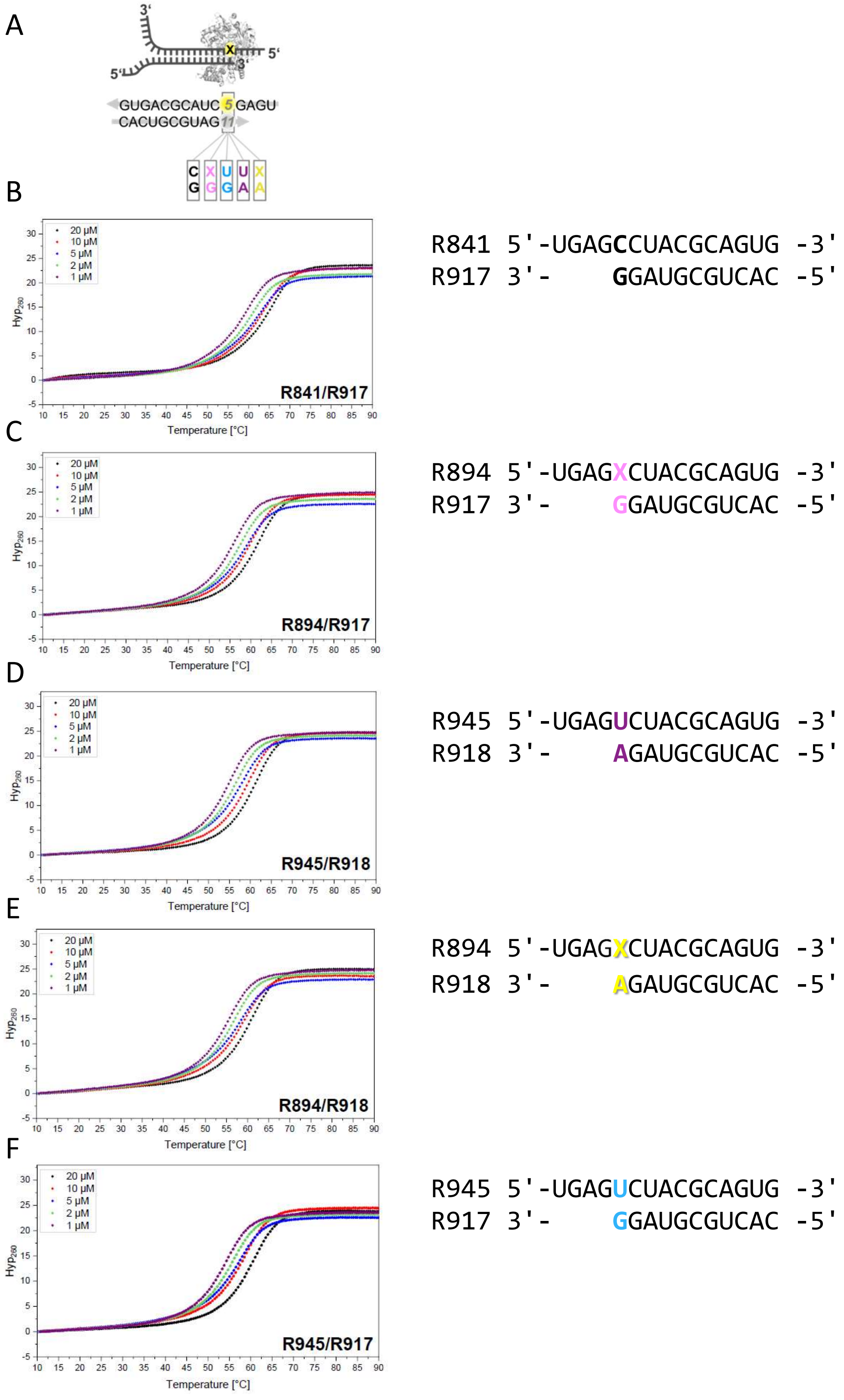


**Figure S12** Concentration dependence of the UV melting curves of the RdRp substrate with 11 base pairs and 4 nt overhang mimicking the stage directly after addition of one nucleotide opposite to template X (A). Hyperchromicity at 260 nm.

A

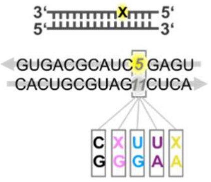


B

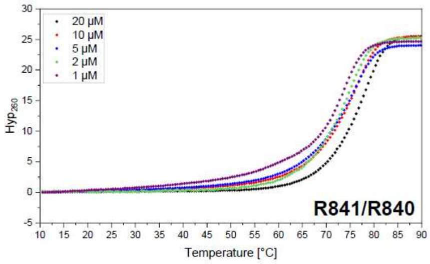


R841 5'-UGAG**C**CUACGCAGUG -3'
R840 3'-ACUC**G**GAUGCGUCAC -5'

C

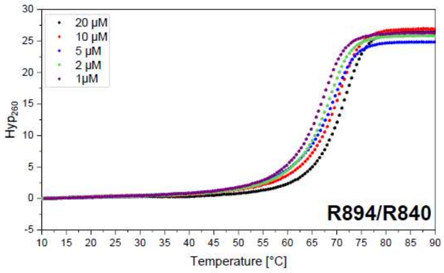


R894 5'-UGAGXCUACGCAGUG -3'
R840 3'-ACUCGGAUGCGUCAC -5'

D

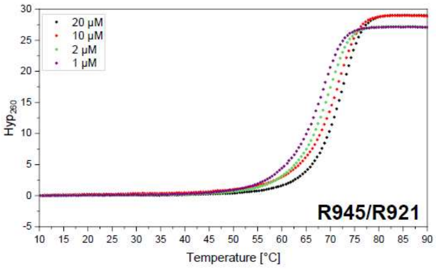


R945 5'-UGAG**U**CUACGCAGUG -3'
R921 3'-ACUC**A**GAUGCGUCAC -5'

E

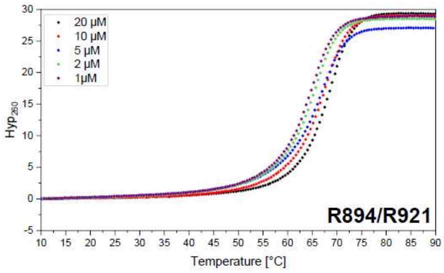


R894 5'-UGAGXCUACGCAGUG -3'
R921 3'-ACUCAGAUGCGUCAC -5'

F

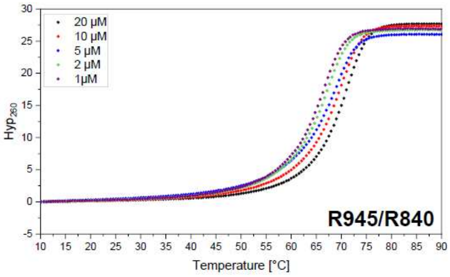


R945 5'-UGAGUCUACGCAGUG -3'
R840 3'-ACUCGGAUGCGUCAC -5'

**Figure S13** Concentration dependence of the UV melting curves of the fully elongated RdRp substrate with 15 base pairs and more internal modification (positions 5/11 as indicated in A). Hyperchromicity at 260 nm.

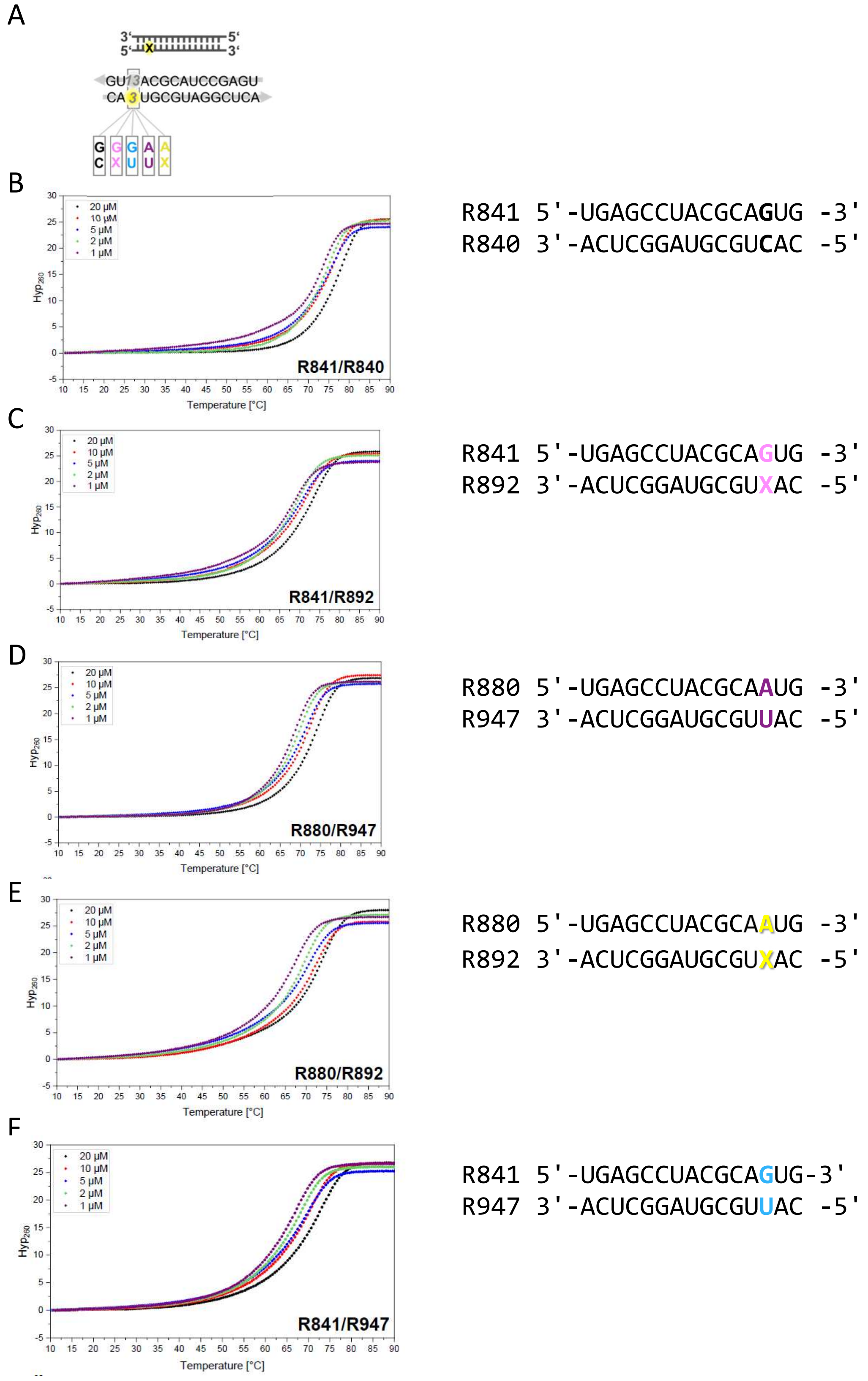


**Figure S14** Concentration dependence of the UV melting curves of the fully elongated RdRp substrate with 15 base pairs and more terminal modification (positions 13/3 as indicated in A). Hyperchromicity at 260 nm.

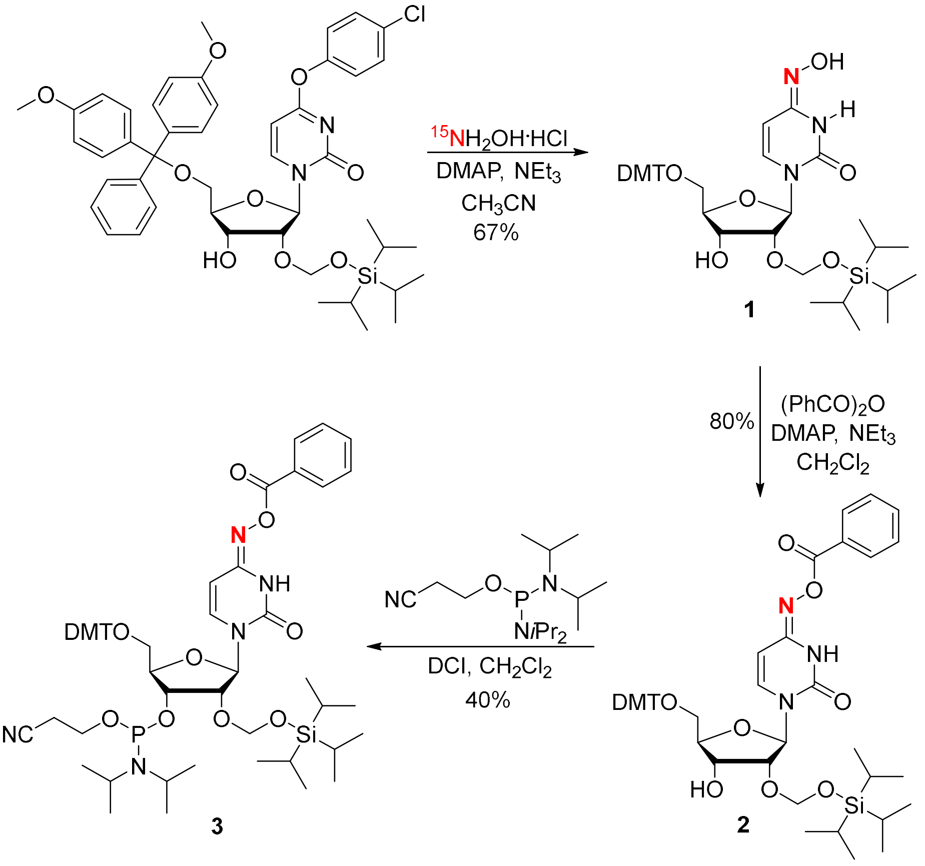


**Scheme S1** Synthesis of $^{15}N(4)$-labeled NHC-phosphoramidite **3** ($^{15}N(4)$ NHC-PA). 5’-*O*-DMT-2’-*O*-TOM-*O*4-chlorophenyluridine (A) was synthesized over three steps starting with uridine, followed by 5'-DMT, 2'-TOM protection and activation with 4-chlorophenol, as previously reported.[4],[5] The $^{15}N$ label was introduced using $^{15}NH_2OH·HCl$ (provided by Cambridge Isotope Laboratories, Inc.) by substitution of the chlorophenol group, yielding compound **1**. Benzoyl protection of the hydroxy group of N4 with benzoic anhydrate was followed by phosphitylation of compound **2** with 2-cyanoethyl-*N*,*N*,*N*’,*N*’-tetraisopropylphosphorodiamidite and 4,5-dicyano-imidazol (DCI) to get $^{15}N(4)$ NHC-PA **3** based on our earlier report. [5,6]

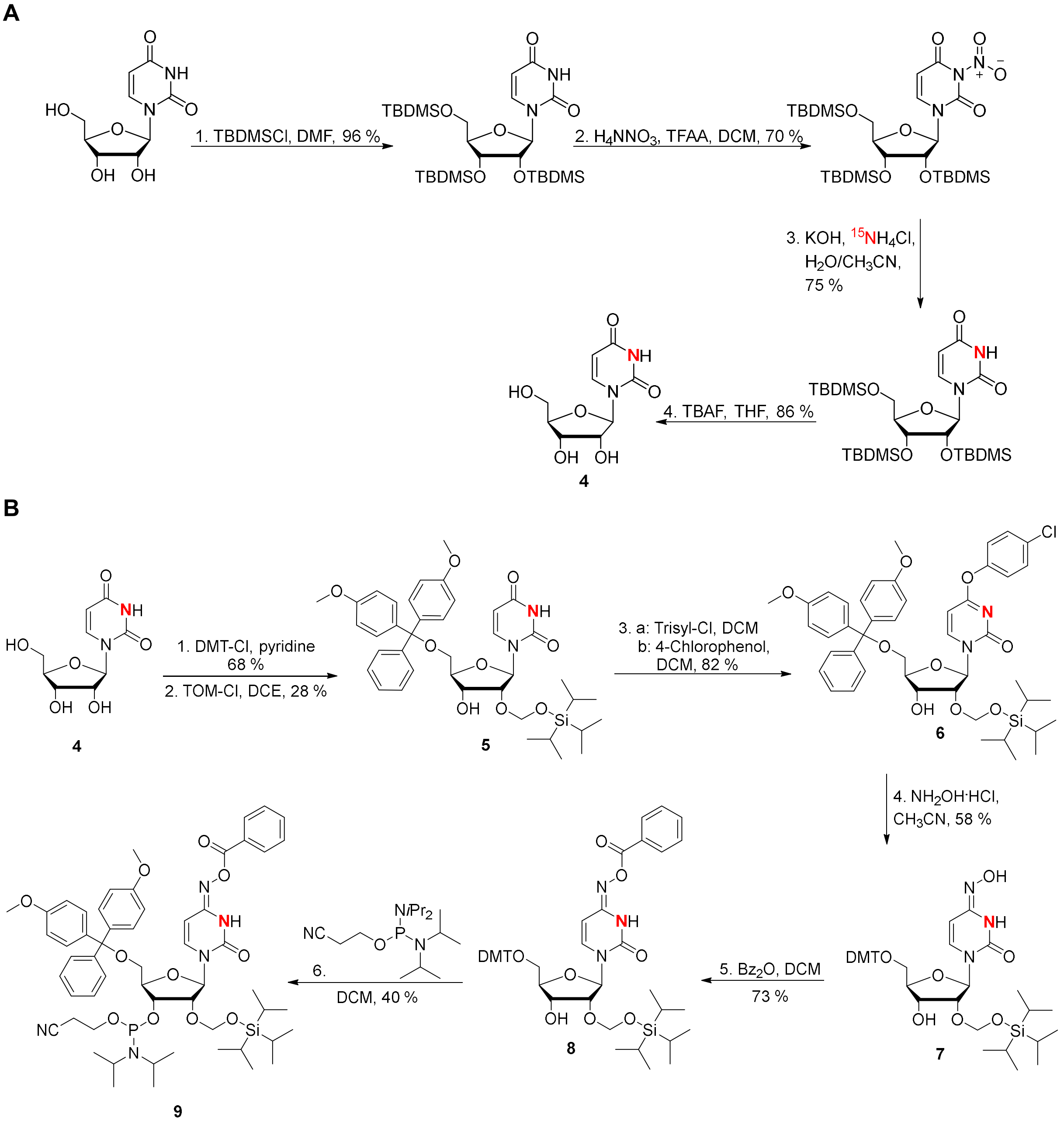


**Scheme S2** A) Synthesis of $^{15}$N(3)-labeled uridine (compound **4**) according to Neuner *et al*.[7] B) Synthesis of $^{15}$N(3)-labeled NHC phosphoramidite ($^{15}$N(3) NHC-PA, compound **9**) according to our previously reported protocol.[5,6]

## Experimental procedures

### General Information

All reactions were performed under inert nitrogen atmosphere. The chemicals were purchased 'Pro analysis'- or 'For synthesis' quality and used without additional purification. Dry solvents ($CH_2Cl_2$, MeCN) were used directly from solvent purification system (SPS), while solvents (technical quality) for workup and purification were distilled prior usage. Acetonitrile (DNA synthesis quality) was dried over molecular sieves for solid-phase synthesis and the phosphoramidite solutions were stored in the fridge with molecular sieves. For column chromatography silica gel (Kieselgel 60, Merck) with a particle size of 0.040-0.063 was used. TLC was performed on Alugram® aluminium sheets (Machery-Nagel, UV visualization, 254 nm). NMR spectra of the compounds 1-9 were recorded on a Bruker Avance III HD 400 spectrometer and of oligonucleotides on Bruker Avance III HD 600 spectrometer. Chemical shifts (δ) of the compounds are given in ppm, referred to the respective solvent signals as internal standards ($D_2O$: $^1H$ = 4.79 ppm; $CDCl_3$: $^1H$ = 7.26 ppm, $^{13}C$ = 77.16 ppm; DMSO-$d_6$: $^1H$ = 2.50 ppm, $^{13}C$ = 39.52 ppm). $^{15}N$ chemical shifts (δ) of NMR spectra of compounds 1, 2, 3 and 7 were indirectly referenced from the residual $^1H$ resonance of the solvent (DMSO-$d_6$: $^1H$ = 2.50 ppm) using the Ξ value from Wishart *et al.*[8] $^{31}P$ chemical shifts (δ) of NMR spectra of compounds 3 and 9 were indirectly referenced from the residual $^1H$ resonance of the solvent ($CDCl_3$: $^1H$ = 7.26 ppm) using the IUPAC recommended Ξ value. $^1H$ chemical shifts (δ) of oligonucleotides are given in ppm, but relative to the internal standard 3-(Trimethylsilyl)-1-propanesulfonic acid (DSS). $^{13}C$ and $^{15}N$ chemical shifts of oligonucleotides were indirectly referenced from the $^1H$ chemical shift of DSS according to Wishart *et al.*[8] ESI-MS of the purified products and the oligonucleotides were measured on a Bruker micrOTOF-Q III spectrometer. They are given as the detected mass-to-charge ratio (m/z), compared to the calculated monoisotopic mass.

**Synthetic procedures and NMR spectra for compounds 1-9**

**Compound 1**

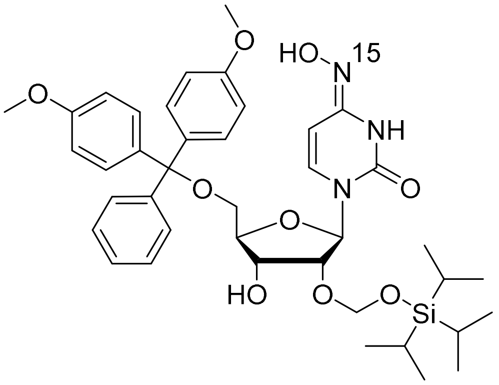


To 5′-*O*-DMT-2′-*O*-TOM-*O*4-chlorophenyluridine (115 mg, 136 µmol, 1.0 eq.) in anhydrous $CH_3CN$, 4-Dimethylaminopyridine (DMAP, 50.0 mg, 409 µmol, 3.0 eq.) and $NEt_3$ (190 µL, 1.36 mmol, 10.0 eq.) were added under nitrogen atmosphere. After addition of $^{15}$N-hyroxylamine hydrochloride ($^{15}NH_2OH$, 38.4 mg, 545 µmol, 4.0 eq.) the reaction mixture was stirred for 24h at ambient temperature, diluted with $CH_2Cl_2$ and washed with saturated aqueous $NaHCO_3$ (2x). The organic phase was dried over $Na_2SO_4$ and the solvent was removed under reduced pressure. The crude product was purified by column chromatography (*n*-hexane:EtOAc + 1% $NEt_3$ 1:2 to 2:1) to yield compound **1** (68.0 mg, 90.8 µmol, 67%) as a colorless foam.

**$^1$H-NMR** ($CDCl_3$, 400 MHz): *δ* (ppm) = 8.55 (s, 1H, N3-H), 7.41 – 7.36 (m, 2H, DMT), 7.32 – 7.26 (m, 6H, DMT), 7.25 – 7.20 (m, 1H, DMT), 7.16 (d, *J* = 8.3 Hz, 1H, H-6), 6.86 – 6.81 (m, 4H, DMT), 6.03 (d, *J* = 4.4 Hz, 1H, H-1'), 5.25 (d, *J* = 8.2 Hz, 1H, H-5), 5.21 (d, *J* = 4.7 Hz, 1H, $OCH_2O$), 4.42 (s, 1H, H-3'), 4.24 (t, *J* = 4.8 Hz, 1H, H-2'), 4.09 (dt, *J* = 5.1, 2.6 Hz, 1H, H-4'), 3.80 (s, 6H, $OCH_3$), 3.49 – 3.39 (m, 2H, H-5'), 3.12 (s, 1H, C2'-O*H*), 1.18 – 1.01 (m, 21H, $Si(CH(CH_3)_2)_3$).

**$^{13}$C-NMR** (101 MHz, $CDCl_3$): *δ* (ppm) = 158.69 ($C_q$-$OCH_3$), 149.58 (C2), 145.33 (d, *J* = 3.0 Hz, C4), 144.42 ($C_q$- DMT), 135.77 ($C_q$-DMT), 135.48 ($C_q$-DMT), 130.68 (C6), 130.31 (DMT), 128.36 (DMT), 128.09 (DMT), 127.19 (DMT), 113.39 (DMT) 98.47 (d, *J* = 10.2 Hz, C5), 90.79 ($OCH_2O$), 87.07, 87.03 (C1', $C_q$-DMT), 83.47 (C4'), 82.33 (C2'), 70.24 (C3'), 62.92 (C5'), 55.44 ($OCH_3$), 55.43 ($OCH_3$), 17.95 ($Si(CH(CH_3)_2)_3$), 11.99 ($Si(CH(CH_3)_2)_3$).

**HR-MS (ESI+)**: Exact mass calculated for $C_{40}H_{53}NaN_2{}^{15}NO_9Si$ $[M+Na]^+$: 771.34136, found: 771.34164

**$^1$H-NMR** (400 MHz, $d_6$-DMSO): *δ* (ppm) = 9.99 (d, *J* = 2.2 Hz, 1H, $^{15}$N-O*H*), 9.53 (d, *J* = 2.2 Hz, 1H, N3-H), 7.40 – 7.36 (m, 2H, DMT), 7.33 – 7.28 (m, 2H, DMT), 7.28 – 7.24 (m, 4H, DMT), 7.23 – 7.20 (m, 1H, DMT), 6.91 – 6.87 (m, 4H, DMT), 6.86 (d, *J* = 8.4 Hz, 1H, H-6), 5.87 (d, *J* = 6.1 Hz, 1H, H-1'), 5.38 (dd, *J* = 8.4, 1.7 Hz, 1H, H-5), 5.13 (d, *J* = 5.7 Hz, 1H, C2'-O*H*), 4.95 (d, *J* = 5.3 Hz, 1H, $OCH_2O$), 4.90 (d, *J* = 5.3 Hz, 1H, $OCH_2O$), 4.24 (dd, *J* = 5.8 Hz, 1H, H-2'), 4.10 – 4.05 (m, 1H, H-3'), 3.95-3.88 (m, 1H, H-4'), 3.73 (s, 6H, $OCH_3$), 3.19 (qd, *J* = 10.6, 3.8 Hz, 2H, H-5'), 1.05 – 0.92 (m, 21H, $Si(CH(CH_3)_2)_3$).

**$^{13}$C-NMR** (101 MHz, $d_6$-DMSO): *δ* (ppm) = 158.12 ($C_q$-$OCH_3$), 149.27 (C2), 144.68 (Cq-DMT), 143.18 (d, *J* = 2.5 Hz, C4), 135.43 ($C_q$-DMT), 135.33 ($C_q$-DMT), 129.77 (DMT, C6), 127.91 (DMT), 127.72 (DMT), 126.79 (DMT), 113.24 (DMT), 98.76 (d, *J* = 11.3 Hz, C5), 88.29 ($OCH_2O$), 85.93, 85.72 (C1', $C_q$-DMT), 83.07 (C4'), 76.18 (C2'), 68.91 (C3'), 63.65 (C5'), 55.05 ($OCH_3$), 17.65 ($Si(CH(CH_3)_2)_3$), 11.38 ($Si(CH(CH_3)_2)_3$).

**$^{15}$N-NMR** (41 MHz, $d_6$-DMSO): *δ* (ppm) = 287.8 (N4), 118.3 (N3).

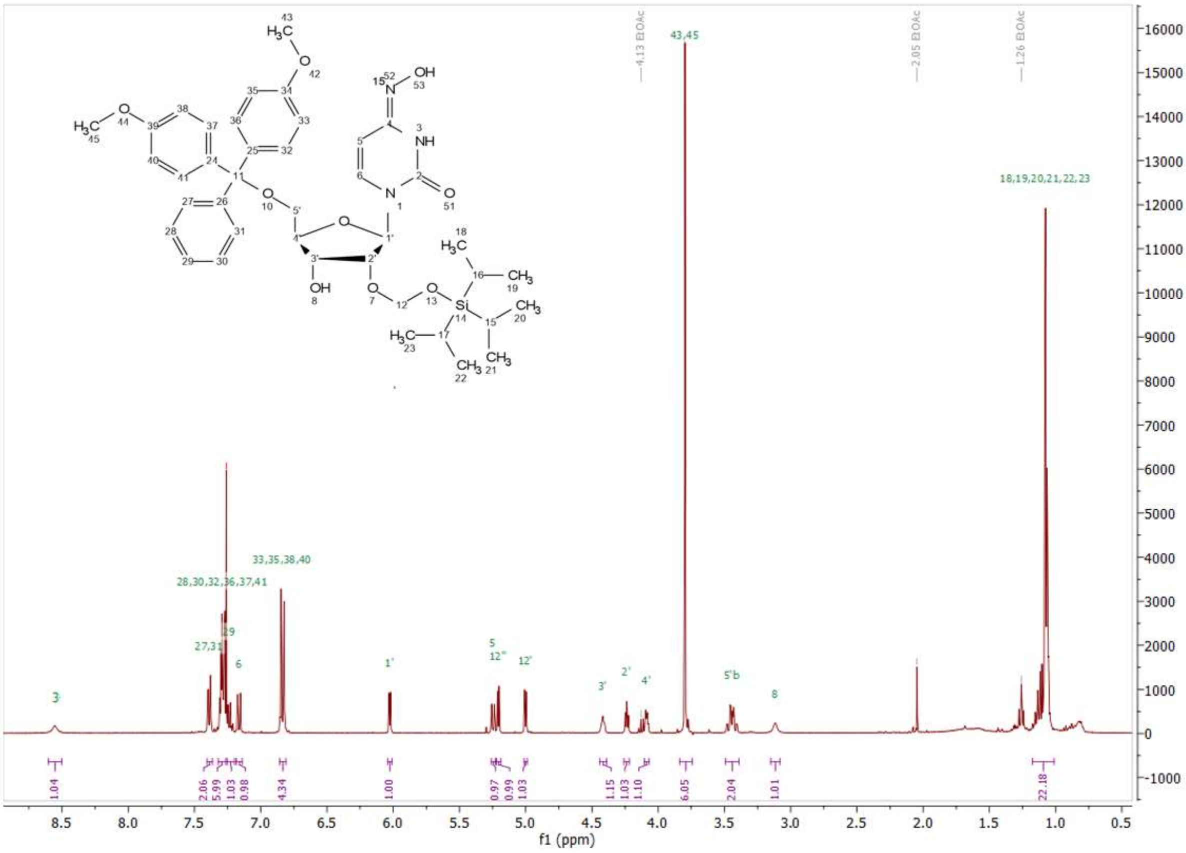


$^{1}$H-NMR (400 MHz, $CDCl_3$) of compound **1**.

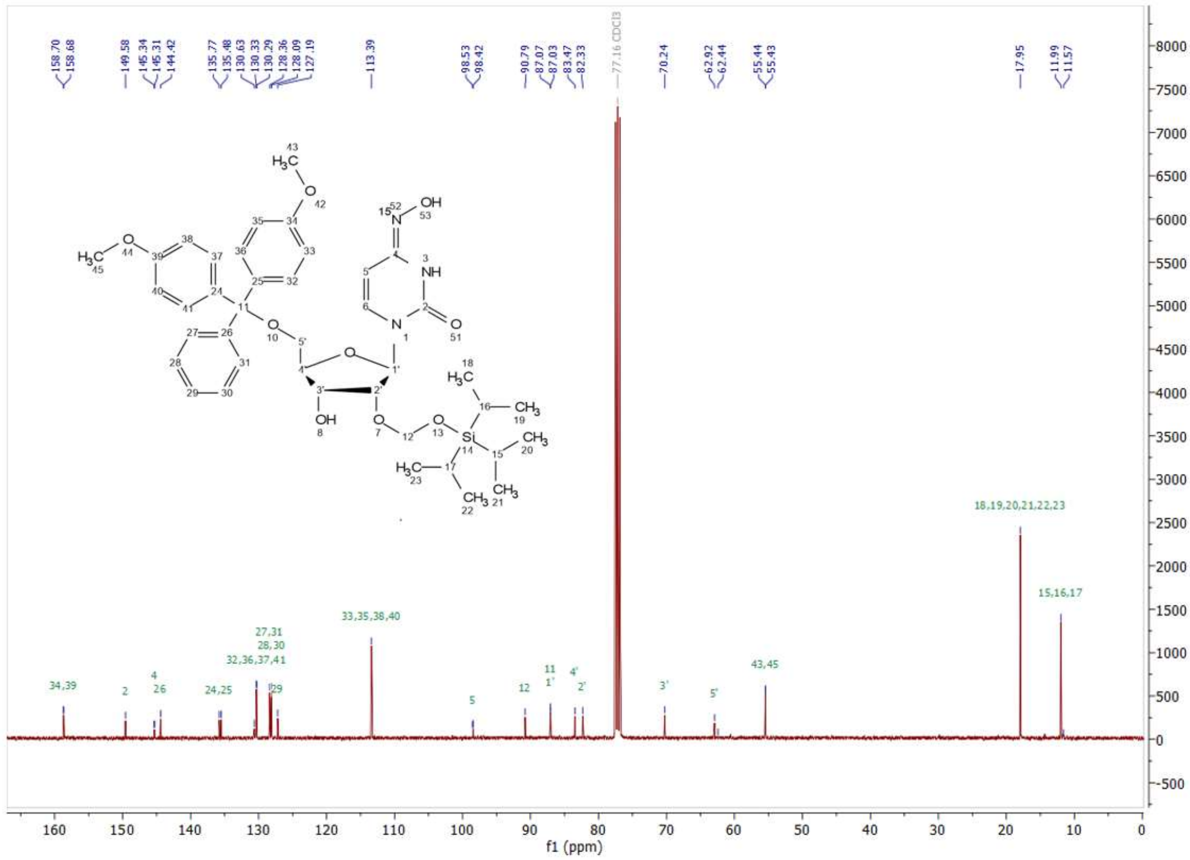


$^{13}$C-NMR (101 MHz, $CDCl_3$) of compound **1**.

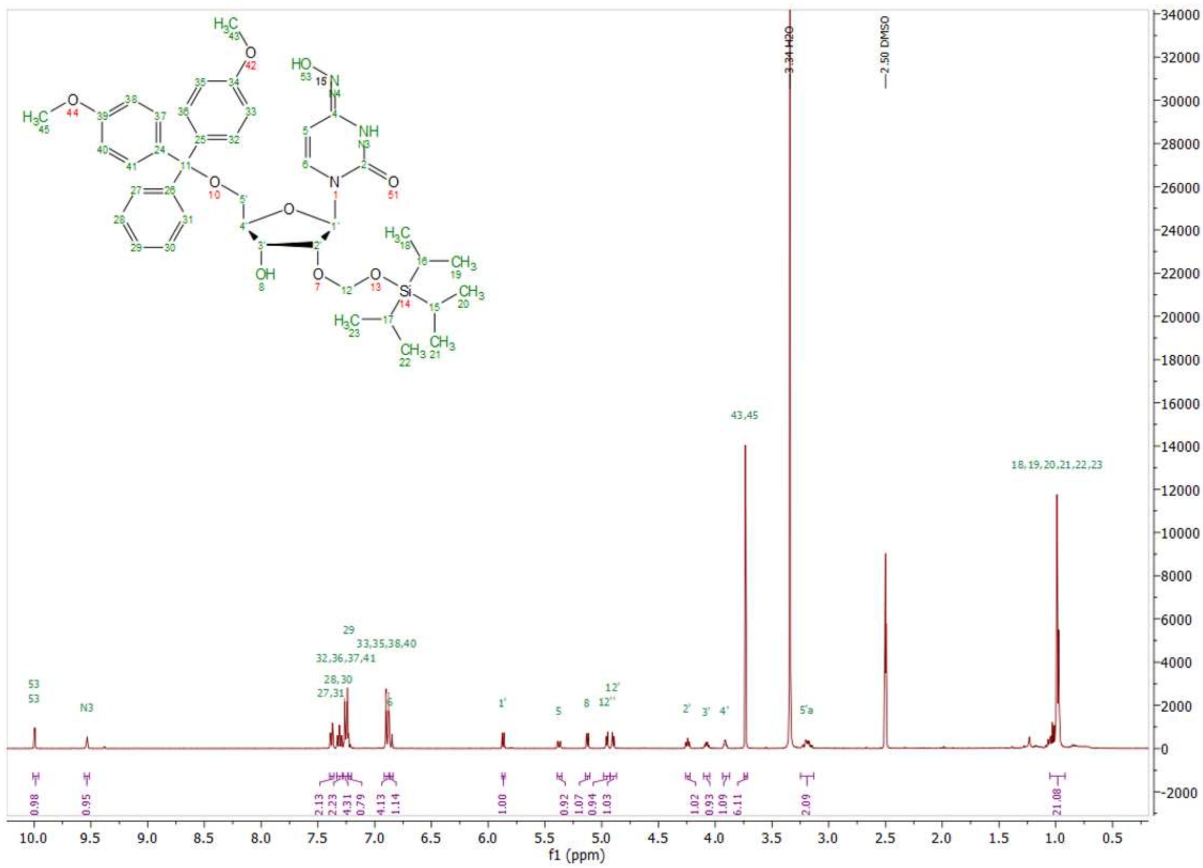

$^1$H-NMR (400 MHz, $d_6$-DMSO) of compound **1**.

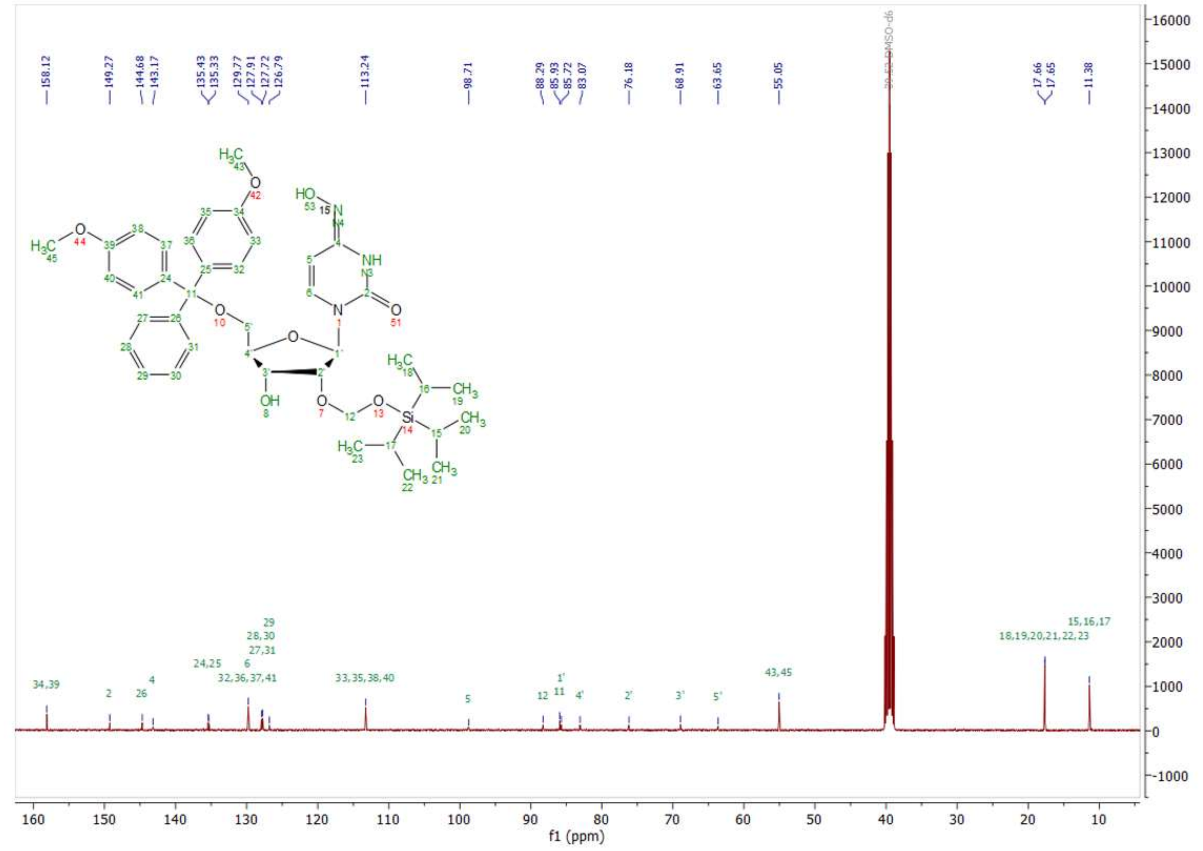

$^{13}$C-NMR (101 MHz, $d_6$-DMSO) of compound **1**.

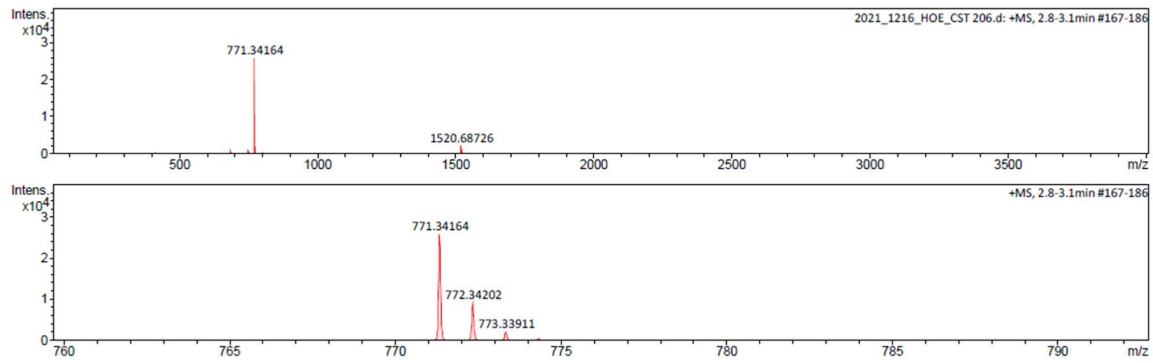


| Meas. m/z | # | Ion Formula | m/z | err [ppm] | mSigma | # mSigma | Score | rdb | e⁻ Conf | N-Rule |
|---|---|---|---|---|---|---|---|---|---|---|
| 771.34164 | 1 | C40H53N2NaO9Si^15N | 771.34136 | -0.36 | 81.3 | 1 | 100.00 | 16.5 | even | - |

ESI-MS of compound **1**.

**Compound 2**

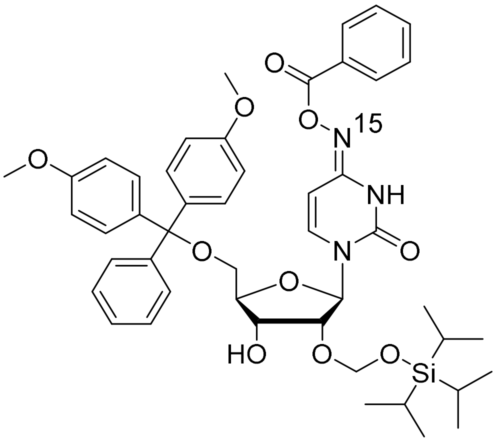


5'-*O*-(4,4'-Dimethoxytrityl)-[15]*N*[4]-hydroxy-2'-*O*-(triisopropylsilyloxy)methylcytidine (62.0 mg, 82.8 µmol, 1.0 eq., compound **1**) was dissolved in anhydrous $CH_2Cl_2$ under nitrogen atmosphere and treated with 4-Dimethylaminopyridine (DMAP, 20.2 mg, 166 µmol, 2.0 eq.) and $NEt_3$ (46.2 µL, 331 µmol, 4.0 eq.). Benzoic anhydride (18.5 mg, 81.95 µmol, 0.99 eq.) was added in three portions within 3 h and stirred for additional 3 h at ambient temperature. The solvent was removed under reduced pressure. The crude product was purified by column chromatography (*n*-hexane:EtOAc + 1% $NEt_3$ 2:1) to yield compound **2** (57.0 mg, 66.8 µmol, 80%) as a colorless foam.

**$^1$H-NMR** ($CDCl_3$, 400 MHz): $\delta$ (ppm) = 8.08 – 8.12 (s, 1H, H-3 imino), 8.02 – 8.06 (m, 2H, bz), 7.59 – 7.64 (m, 1H, bz), 7.47 – 7.52 (m, 2H, bz), 7.43 – 7.46 (dd, *J* = 0.7, 8.3 Hz, 1H, H-6), 7.36 – 7.41 (m, 2H, DMT), 7.26 – 7.33 (m, 6H, DMT), 7.20 – 7.26 (m, 1H, DMT), 6.79 – 6.87 (m, 4H, DMT), 6.05 (d, *J* = 4.7 Hz, 1H, H-1'), 5.49 (dd, *J* = 8.2, 1.0 Hz, 1H, H-5), 5.22 (d, *J* = 4.8 Hz, 1H, $OCH_2O$), 5.00 (d, *J* = 4.8 Hz, 1H, $OCH_2O$), 4.45 – 4.50 (m, 1H, H-3'), 4.27 (dd, *J* = 4.9 Hz, 1H, H-2'), 4.12 – 4.15 (m, 1H, H-4'), 3.78 – 3.82 (m, 6H, $OCH_3$), 3.43 – 3.51 (m, 2H, H-5'), 3.07 – 3.12 (d, *J* = 4.3 Hz, 1H, C2'-O*H*), 1.04 – 1.17 (m, 21H, $Si(CH(CH_3)_2)_3$).

**$^{13}$C-NMR** (101 MHz, $CDCl_3$): $\delta$ (ppm) = 163.51 (C(O)bz), 158.86 ($C_q$-$OCH_3$), 158.82 ($C_q$-$OCH_3$), 149.56 (C4), 148.44 (C2), 144.47 ($C_q$-DMT), 135.38 ($C_q$-DMT), 135.11 ($C_q$-DMT), 133.91 (C6), 133.67 (bz), 130.32 (DMT), 130.24 (DMT), 129.73 (bz), 128.83 (bz), 128.24 (DMT), 128.15 (DMT), 127.34 (DMT), 113.41 (DMT), 97.56 (d, *J* = 11.9 Hz, C5), 90.89 ($OCH_2O$), 87.30 ($C_q$-DMT), 87.09 (C1'), 83.89 (C4'), 82.58 (C2'), 70.48 (C3'), 62.94 (C5'), 55.39 ($OCH_3$), 17.95 ($Si(CH(CH_3)_2)_3$), 11.99 ($Si(CH(CH_3)_2)_3$).

**$^{15}$N-NMR** (41 MHz, $d_6$-DMSO): $\delta$ (ppm) = 289.4 (N4), 124.5 (N3).

**HR-MS (ESI+)**: Exact mass calculated for $C_{47}H_{57}NaN_2{}^{15}NO_{10}Si$ $[M+Na]^+$: 875.36758, found: 875.36985

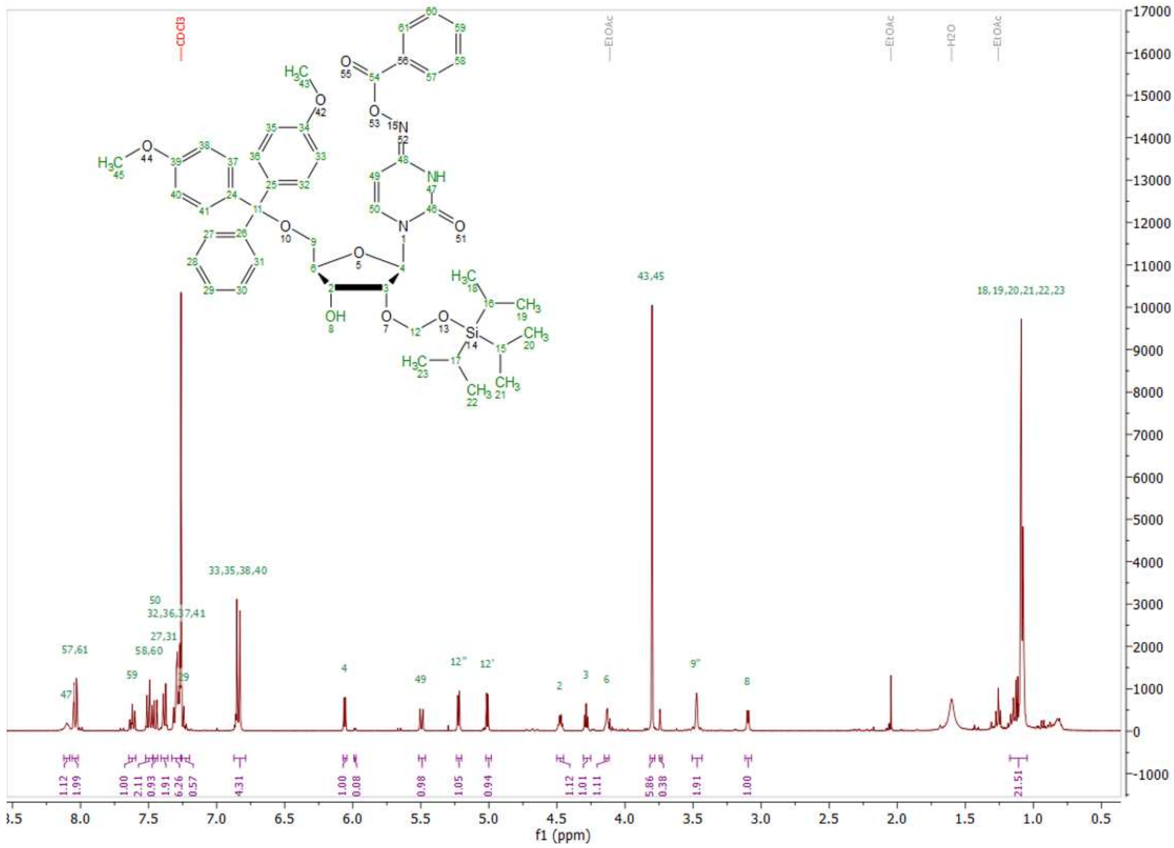

$^1$H-NMR (400 MHz, $CDCl_3$) of compound **2**.

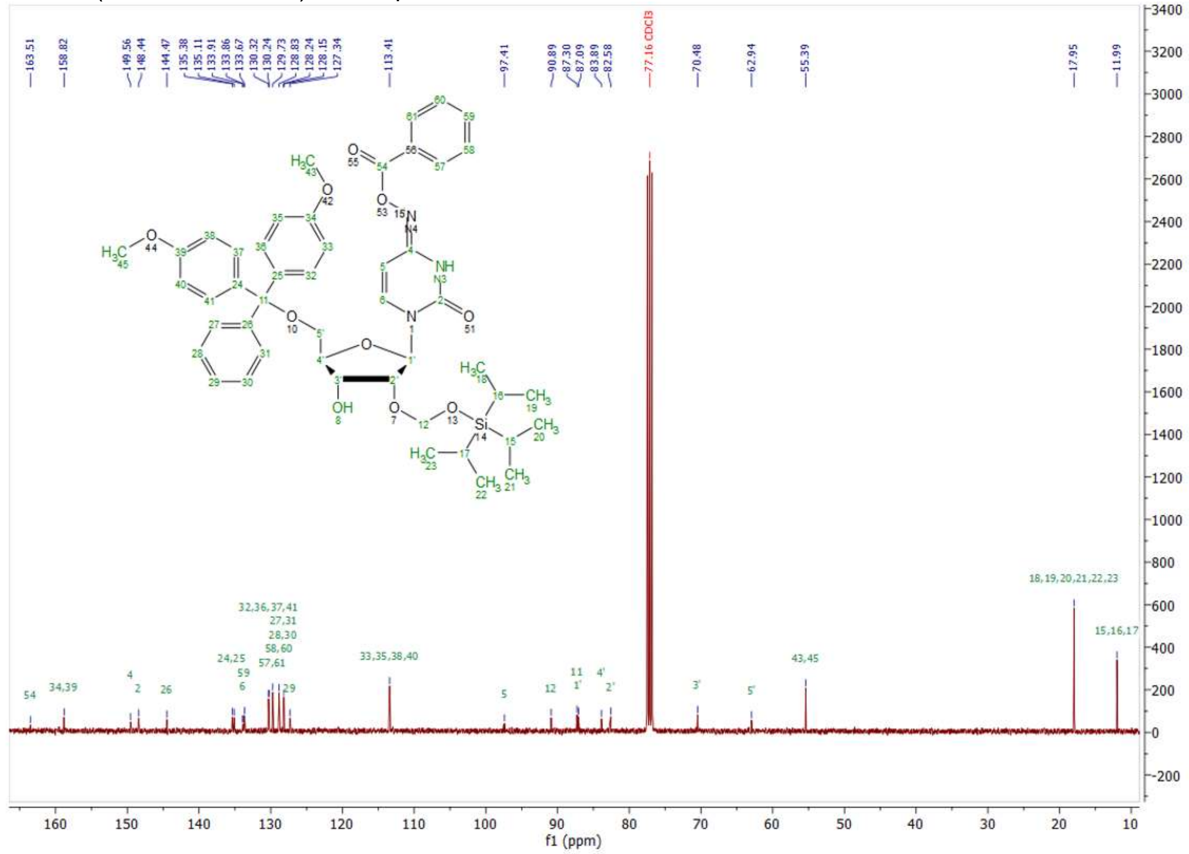

$^{13}$C-NMR (101 MHz, $CDCl_3$) of compound **2**.

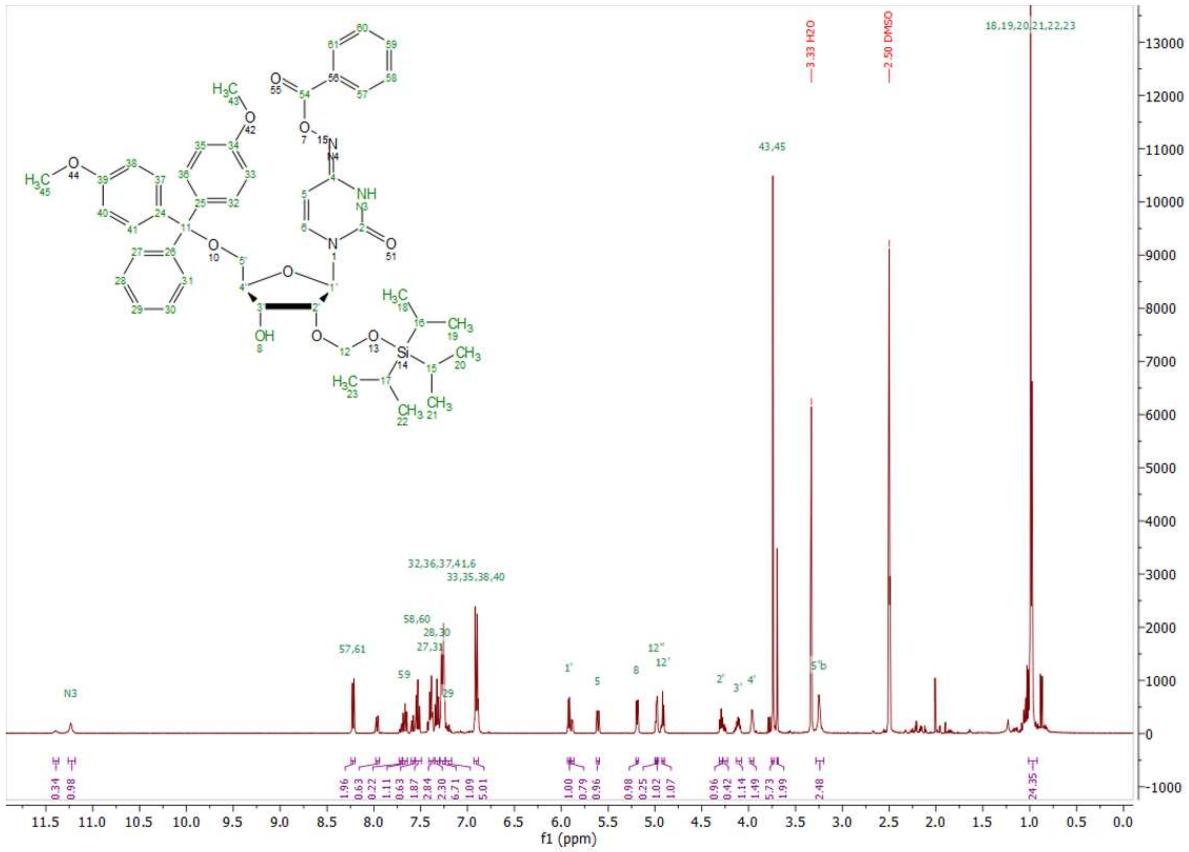

$^{1}$H-NMR (400 MHz, $d_6$-DMSO) of compound **2**.

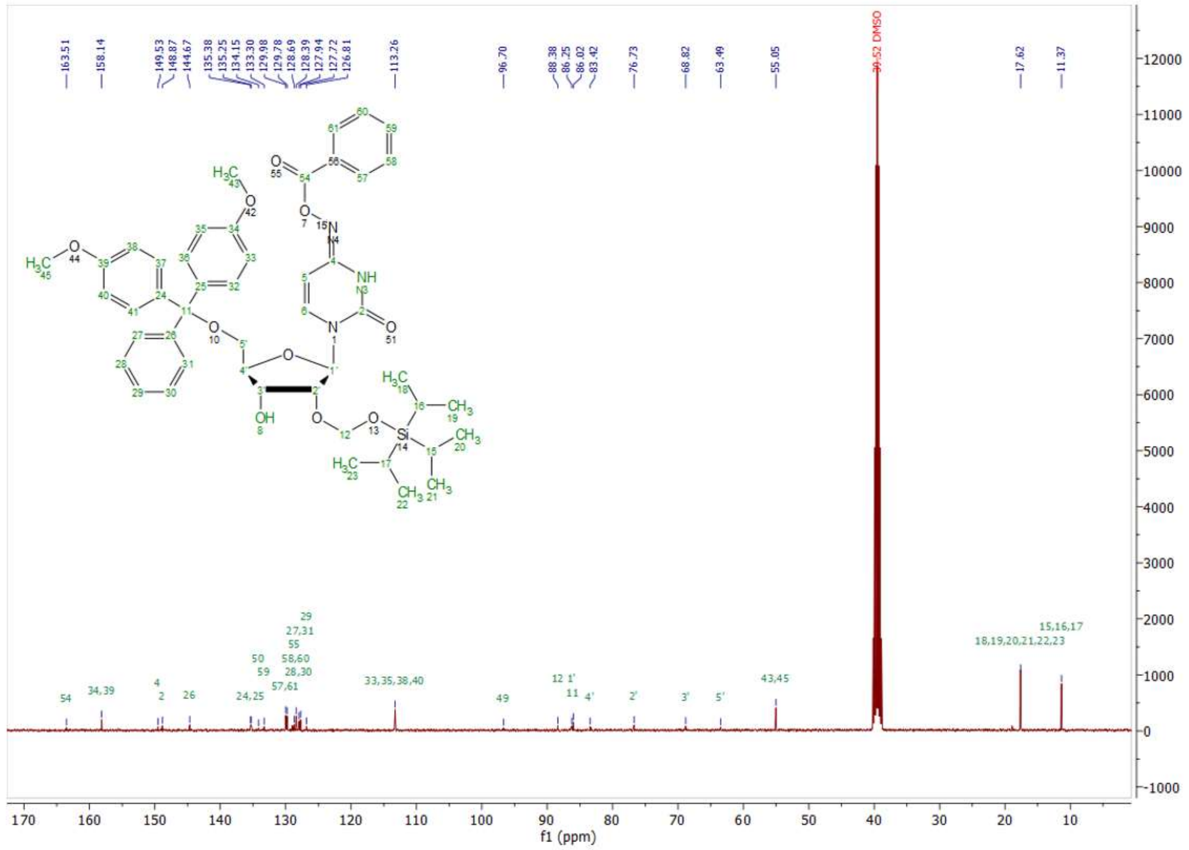

$^{13}$C-NMR (101 MHz, $d_6$-DMSO) of compound **2**.

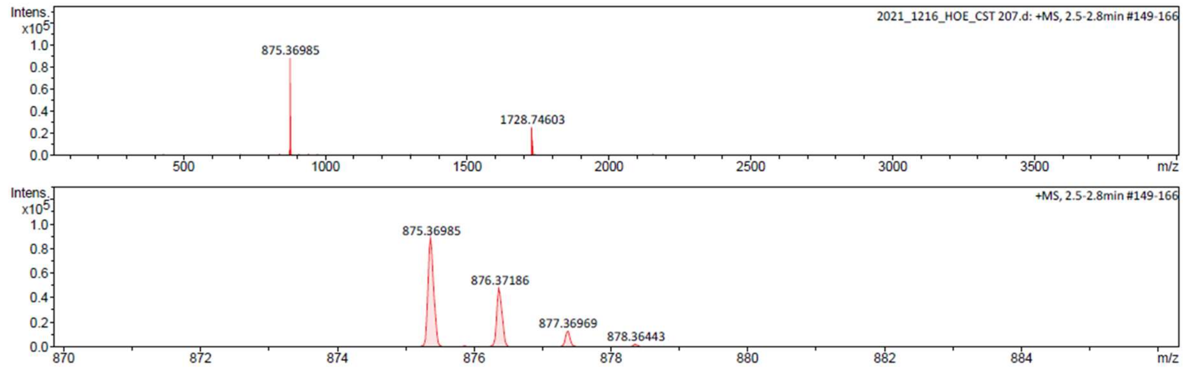


| Meas. m/z | # | Ion Formula | m/z | err [ppm] | mSigma | # mSigma | Score | rdb | e¯ Conf | N-Rule |
|---|---|---|---|---|---|---|---|---|---|---|
| 875.36985 | 1 | C47H57N2NaO10Si^15N | 875.36758 | -2.60 | 41.3 | 1 | 100.00 | 21.5 | even | - |

ESI-MS of compound **2**.

**Compound 3**

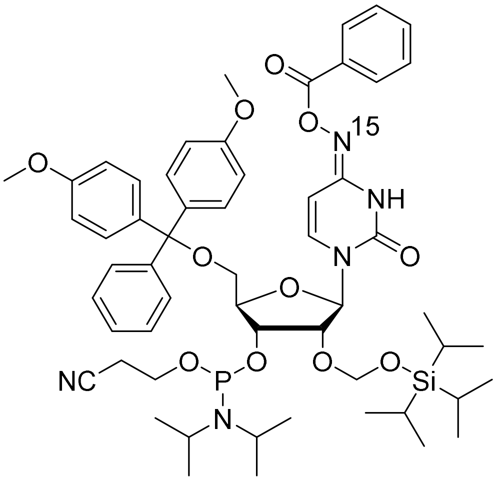

To $N^4$-Benzoyl-5'-*O*-(4,4'-dimethoxytrityl)-$^{15}N^4$-hydroxy-2'-*O*-(triisopropylsilyloxy)methyl cytidine (50.0 mg, 58.6 µmol, 1.0 eq., compound **2**) in anhydrous $CH_2Cl_2$ (2 mL), 4,5-dicyanoimidazol (DCI, 7.62 mg, 64.5 µmol, 1.1 eq.) and 2-Cyanoethyl *N,N,N',N'*-tetraisopropyl phosphoramidite (22.4 µL, 70.6 µmol, 1.2 eq.) were added at 4°C. The reaction mixture was allowed to warm to ambient temperature and stirred for additional 3 h. The solvents was removed under reduced pressure and the crude product was purified by column chromatography (n-hexane:EtOAc + 1% $NEt_3$ 2:1) to yield compound **3** (25.0 mg, 23.7 µmol, 40%) as a colorless foam.

**$^1$H-NMR** ($CDCl_3$, 400 MHz): *δ* (ppm) = 8.02 – 8.06 (m, 6H, bz, diast.), 7.59 – 7.65 (m, 2H, bz, diast.), 7.47 – 7.52 (m, 4H, bz, diast.), 7.41 – 7.43 (d, *J* = 8.2 Hz, 1H, H-6, diast.), 7.39 – 7.42 (p, *J* = 1.2 Hz, 2H, DMT, diast.), 7.35 – 7.38 (d, *J* = 8.1 Hz, 2H, H-6), 7.25 – 7.34 (m, 9H, DMT, diast.), 7.20 – 7.26 (m, 1H, DMT), 6.81 – 6.88 (m, 8H, DMT, diast.), 6.09 – 6.13 (dd, *J* = 5.3, 8.1 Hz, 2H, H-1', diast.), 5.56 – 5.59 (d, *J* = 8.2 Hz, 1H, H-5), 5.52 – 5.55 (d, *J* = 8.2 Hz, 1H, H-5, diast.), 5.00 – 5.05 (m, 3H, $OCH_2O$), 4.95 – 4.97 (d, *J* = 5.1 Hz, 1H, $OCH_2O$, diast.), 4.45 – 4.51 (q, *J* = 5.5 Hz, 1H, H-2'), 4.40 – 4.44 (m, 2H, H-2', diast., H-3'), 4.22 – 4.29 (dd, *J* = 2.9, 5.9 Hz, 2H, H-4'), 4.14 – 4.21 (dd, *J* = 2.9, 5.9 Hz, 1H, H-4', diast.), 3.82 – 4.00 (m, 2H, $CNCH_2$, diast.), 3.78 – 3.81 (d, *J* = 3.0 Hz, 12H, $OCH_3$, diast.), 3.62 – 3.70 (m, 1H, $CNCH_2$, diast.), 3.53 – 3.61 (m, 4H, H-5', $NCH(CH_3)_2$,$CNCH_2$), 3.46 – 3.51 (dd, *J* = 2.6, 10.8 Hz, 1H, H-5', diast.), 3.33 – 3.41 (ddd, *J* = 2.8, 6.8, 10.4 Hz, 2H, H-5', diast.), 2.62 – 2.69 (td, *J* = 2.8, 6.4 Hz, 2H, $POCH_2$, diast.), 2.35 – 2.43 (t, *J* = 6.4 Hz, 2H, $POCH_2$), 1.14 – 1.22 (m, 17H, $NCH(CH_3)_2$, diast.), 0.98 – 1.11 (m, 44H, $Si(CH(CH_3)_2)_3$, diast.).

**$^{31}$P-NMR** (162 MHz, $CDCl_3$): δ (ppm) = 150.60, 150.12.

**$^{15}$N-NMR** (41 MHz, $d_6$-DMSO): *δ* (ppm) = 289.5 (N4), 124.7 (N3).

**HR-MS (ESI+)**: Exact mass calculated for $C_{56}H_{74}NaN_4{}^{15}NO_{11}PSi$ $[M+Na]^+$:1075.47543, found: 1075.47851

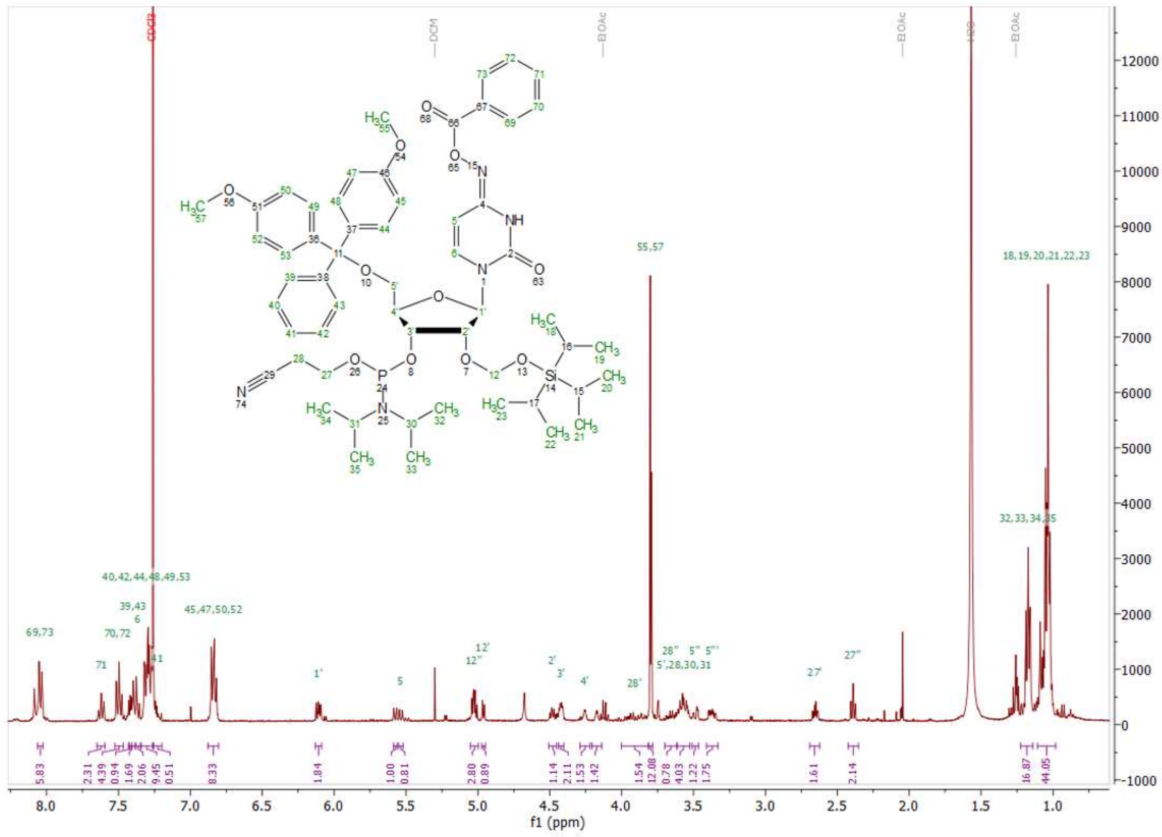

$^{1}$H-NMR (400 MHz, $CDCl_3$) of compound **3**.

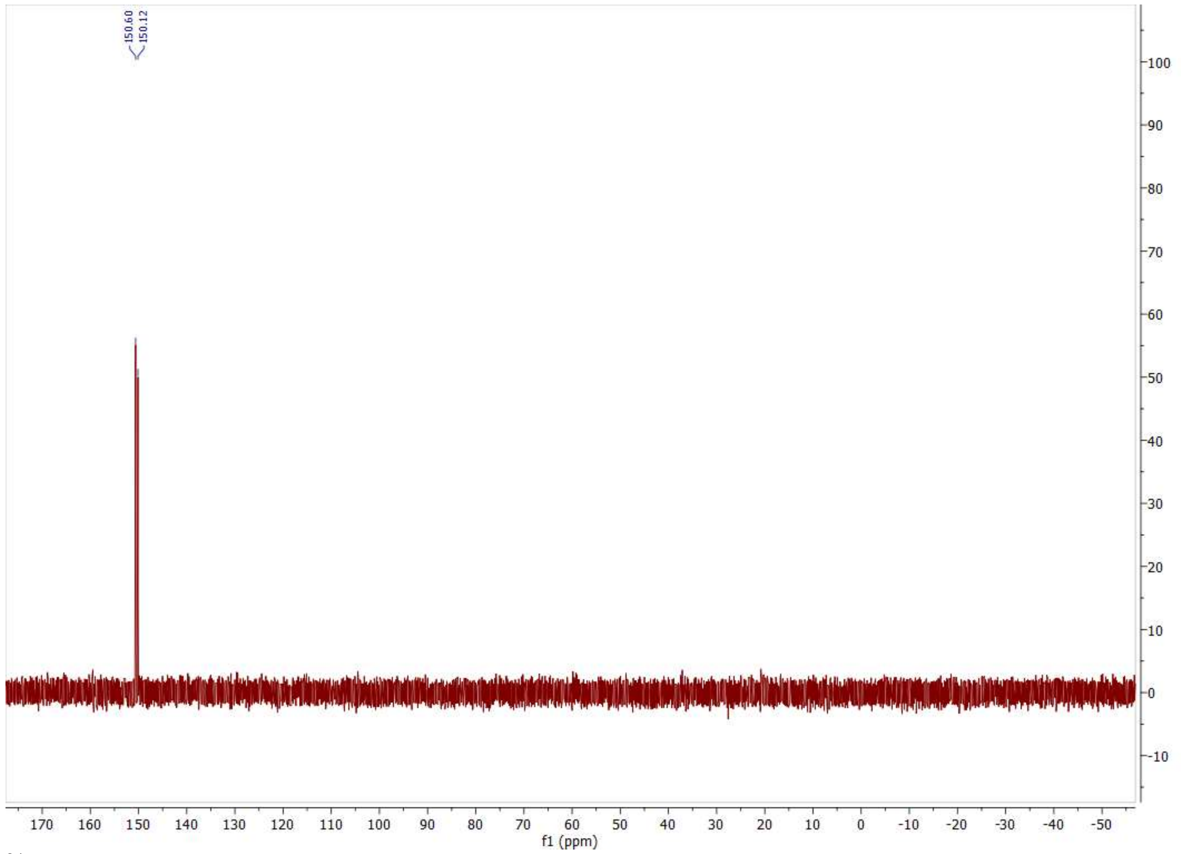

$^{31}$P-NMR (162 MHz, $CDCl_3$) of compound **3**.

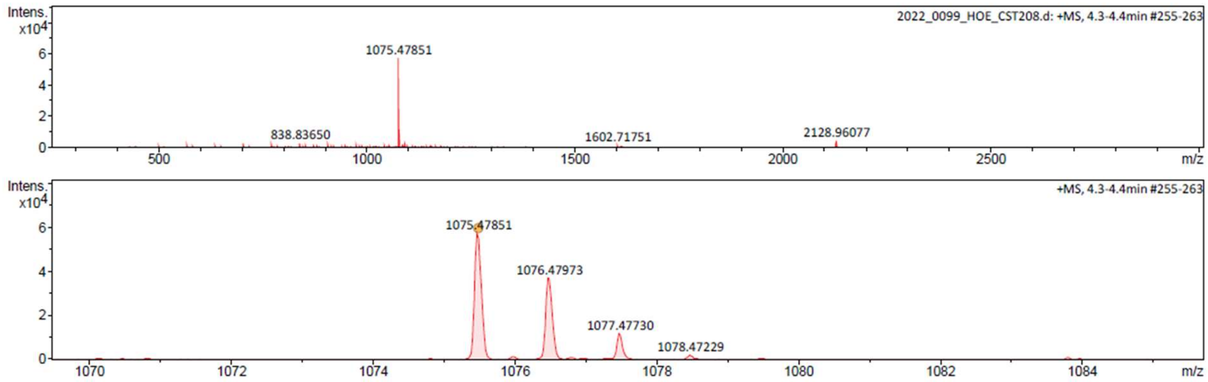


| Meas. m/z | # | Ion Formula | m/z | err [ppm] | mSigma | # mSigma | Score | rdb | e⁻ Conf | N-Rule |
|---|---|---|---|---|---|---|---|---|---|---|
| 1075.47851 | 1 | C56H74N4NaO11PSi^15N | 1075.47543 | -2.87 | 47.1 | 1 | 100.00 | 23.5 | even | - |

ESI-MS of compound **3**.

**Compound 4**

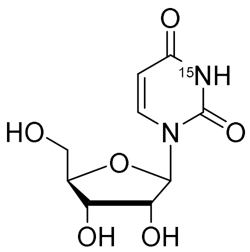

$^{15}$N(3)-labeled uridine (compound **4**) was prepared in 86% yield according to the protocol reported in literature by Neuner *et al.*[7]

**$^1$H-NMR** (400 MHz, $D_2O$): δ (ppm) = 7.85 (d, *J* = 8.1 Hz, 1H, H-6), 5.90 – 5.82 (m, 2H, H-1', H-5), 4.31 (dd, *J* = 5.3 Hz, 4.5 Hz, 1H, H-2'), 4.19 (dd, *J* = 5.4 Hz, 1H, H-3'), 4.13 – 4.06 (m, 1H, H-4'), 3.92 – 3.73 (m, 2H, H-5', H-5'').

**$^{13}$C NMR** (101 MHz, $D_2O$): δ (ppm) = 166.21 (d, *J* = 10.8 Hz, C4), 151.65 (d, *J* = 18.9 Hz, C2), 141.84 (C6), 102.24 (d, *J* = 5.6 Hz, C5), 89.36 (C1'), 84.19 (C4'), 73.70 (C2'), 69.40 (C3'), 60.71 (C5').

**HR-MS (ESI+)**: Exact mass calculated for $C_9H_{12}NaN^{15}NO_6$ $[M+Na]^+$:268.05579, found: 268.05715

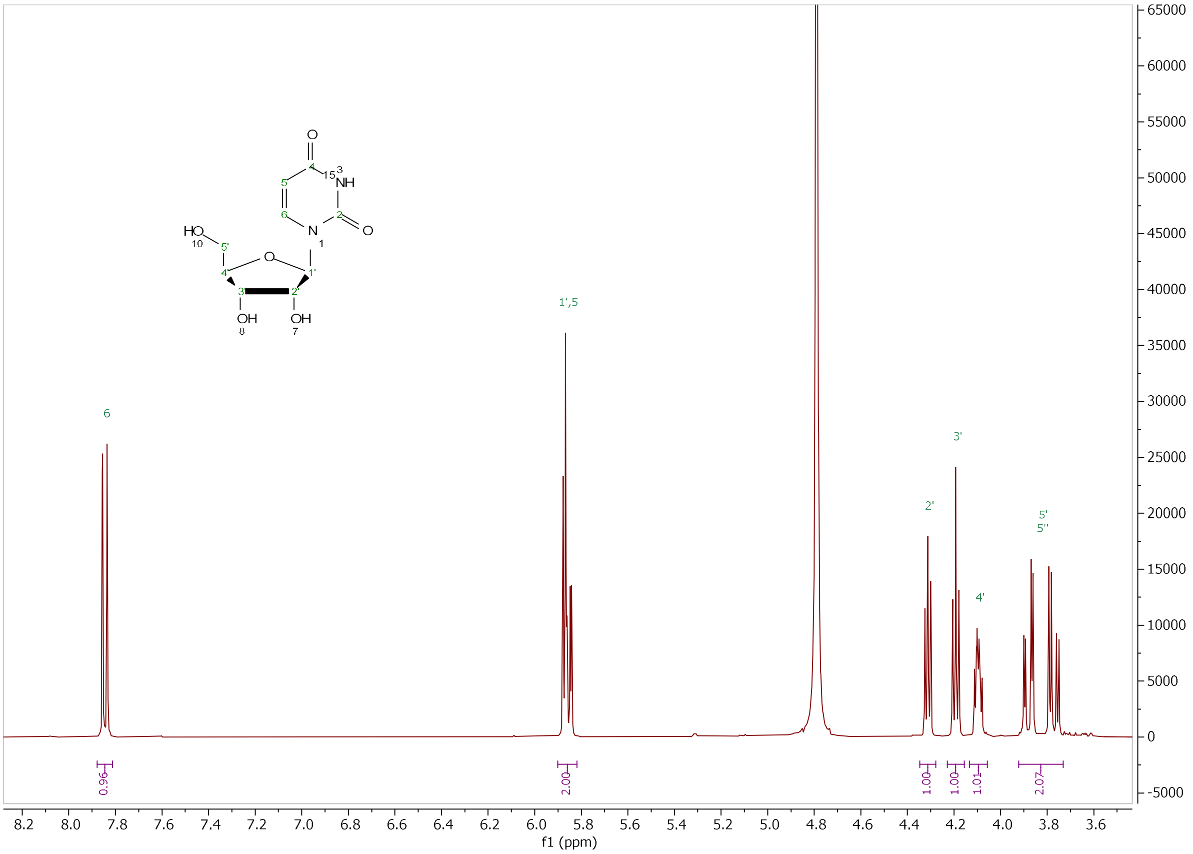


$^1$H-NMR (400 MHz, $D_2O$) of compound **4**.

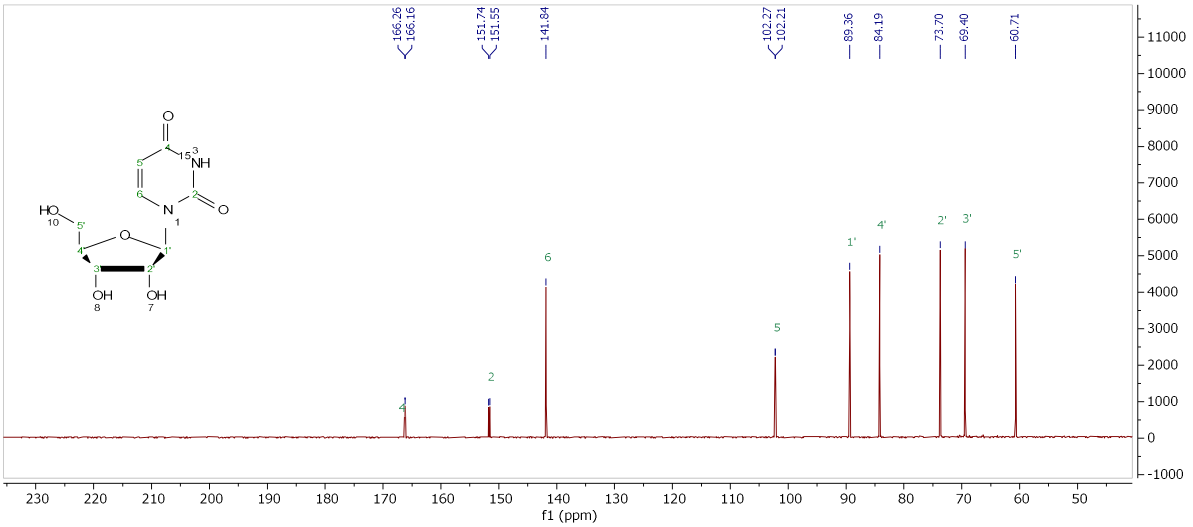


$^{13}$C-NMR (101 MHz, $D_2O$) of compound **4**.

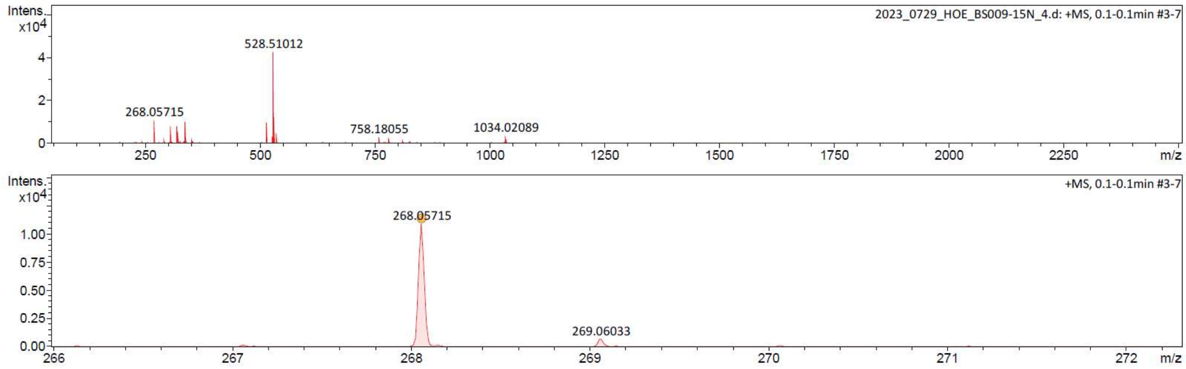


| Meas. m/z | # | Ion Formula | m/z | err [ppm] | mSigma | # mSigma | Score | rdb | e¯ Conf | N-Rule |
|---|---|---|---|---|---|---|---|---|---|---|
| 268.05715 | 1 | C9H12NNaO6^15N | 268.05579 | -5.05 | 28.6 | 1 | 100.00 | 4.5 | even | - |

ESI-MS of compound **4**.

**Compound 5a**

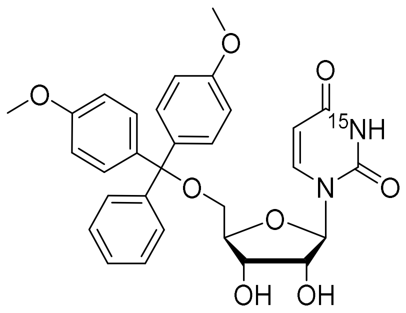


$^{15}N(3)$ uridine (1.15 g, 4.71 mmol, 1.00 eq., compound **4**) was suspended in dry pyridine (20 mL). DMT-Cl (1.66 g, 4.91 mmol, 1.20 eq.) was added in portions over 2 h. After stirring 16 h at room temperature, the solvent (methanol, 5 mL) was evaporated. The residue was dissolved in DCM (100 mL) and washed with 5% citric acid-solution (100 mL), saturated $Na_2CO_3$-solution (200 mL) and brine (100 mL). The organic phase was dried over sodium sulfate and evaporated. The crude product was purified by column chromatography (1% - 5% Methanol in DCM + 1% NEt3) to give 5'-O-DMT-$^{15}N(3)$ uridine (compound **5a**) in 68% yield (1.75 g, 3.2 mmol).

**$^1$H-NMR** (400 MHz, $CDCl_3$): δ (ppm) = 10.32 (d, *J* = 79.8 Hz, 1H, H-3), 8.02 (d, *J* = 8.1 Hz, 1H, H-6), 7.41 – 7.34 (m, 2H, DMT), 7.33 – 7.26 (m, 6H, DMT), 7.24 – 7.15 (m, 1H, DMT), 6.87 – 6.79 (m, 4H, DMT), 5.90 (d, *J* = 2.5 Hz, 1H, H-1'), 5.34 (dd, *J* = 8.1, 2.6 Hz, 1H, H-5), 4.47 – 4.40 (m, 1H, H-3'), 4.34 (dd, *J* = 5.2, 2.6 Hz, 1H, H-2'), 4.21 – 4.13 (m, 1H, H-4'), 3.78 – 3.72 (m, 6H, $OCH_3$ DMT), 3.56 – 3.44 (m, 2H, H-5', H-5'').

**$^{13}$C NMR** (101 MHz, $CDCl_3$): δ (ppm) = 164.07 (d, *J* = 9.6 Hz, C4), 158.79 ($C_q$-DMT), 158.76 ($C_q$-DMT), 151.30 (d, *J* = 18.2 Hz, C2), 144.48 ($C_q$-DMT), 140.62 (C6), 135.43 ($C_q$-DMT), 135.24 ($C_q$-DMT), 130.30 (DMT), 130.21 (DMT), 128.24 (DMT), 128.14 (DMT), 127.25 (DMT), 113.43 (DMT), 113.41 (DMT), 102.39 (d, *J* = 6.4 Hz, C5), 90.60 (C1'), 87.14 ($C_q$-DMT), 83.78 (C4'), 75.57 (C2'), 69.75 (C3'), 61.90 (C5'), 55.37 ($OCH_3$).

**HR-MS (ESI+)**: Exact mass calculated for $C_{30}H_{30}NaN^{15}NO_8$ $[M+Na]^+$:570.18647, found: 570.18521

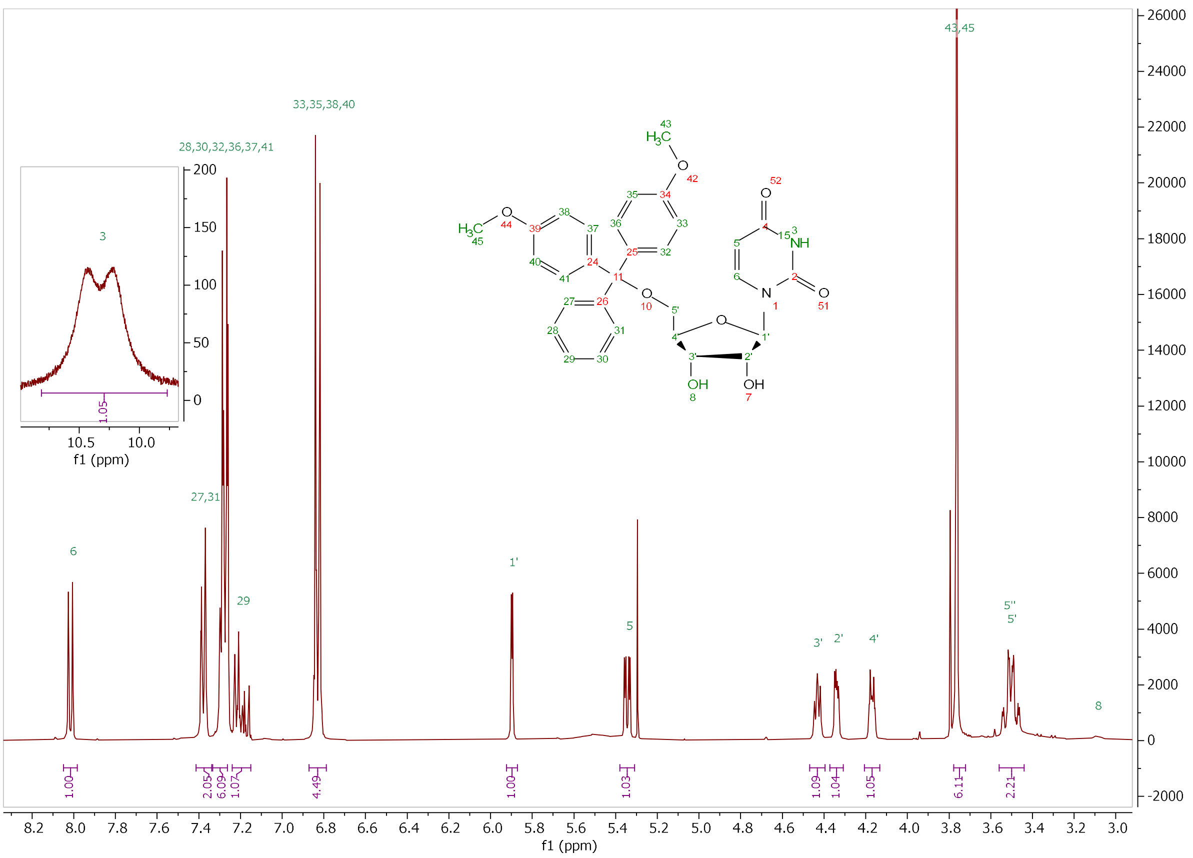

$^{1}$H-NMR (400 MHz, CDCl3) of compound **5a**.

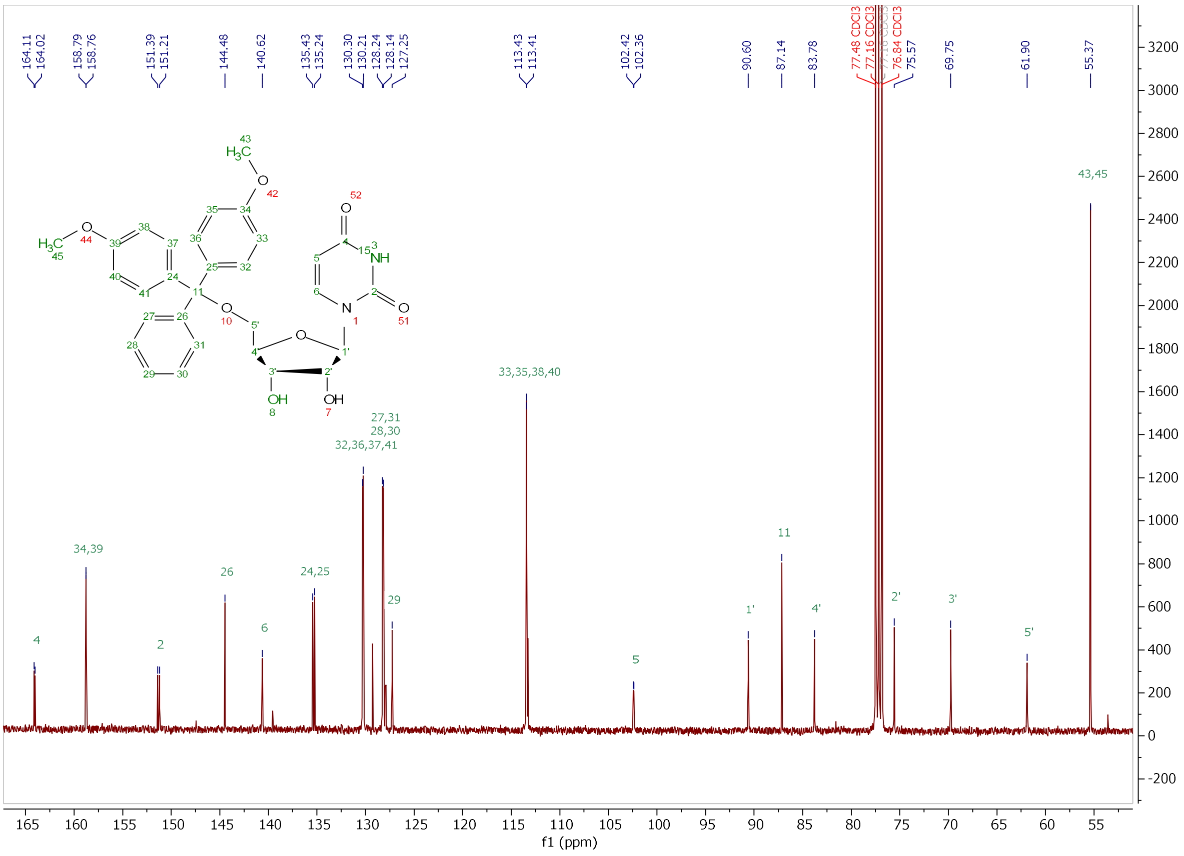

$^{13}$C-NMR (101 MHz, CDCl3) of compound **5a**.

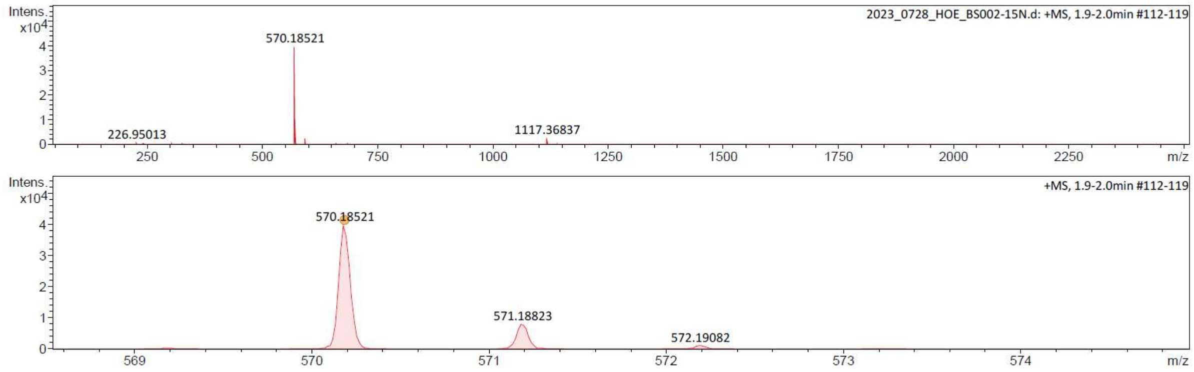


| Meas. m/z | # | Ion Formula | m/z | err [ppm] | mSigma | # mSigma | Score | rdb | e⁻ Conf | N-Rule |
|---|---|---|---|---|---|---|---|---|---|---|
| 570.18521 | 1 | C30H30NNaO8^15N | 570.18647 | 2.22 | 70.3 | 1 | 100.00 | 16.5 | even | - |

ESI-MS of compound **5a**.

**Compound 5**

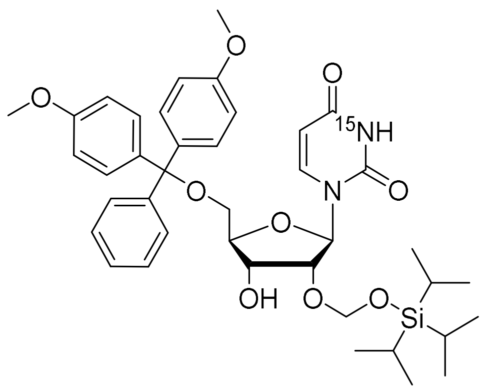


5'-*O*-DMT-$^{15}N(3)$ uridine (916 mg, 1.68 mmol, 1.00 eq., compound **5a**) was dissolved in dry DCE (15 mL). DIPEA (1.04 mL, 5.98 mmol, 3.57 eq.) and $(nBu)_2SnCl_2$ (669 mg, 2.01 mmol, 1.31 eq.) were added and the mixture stirred for 1 h at ambient temperature. TOM-Cl (510 µL, 2.20 mmol, 1.31 eq.) was added and the reaction mixture was stirred for 25 min at 80 °C. The mixture was diluted with DCM (30 mL) and washed with $NaHCO_3$-solution and dried over $Na_2SO_4$. The solvent was evaporated under reduced pressure. The crude product was purified by column chromatography (cyclohexane/EA 2.5:1 to 2:1 + 1% $NEt_3$) to yield 5'-*O*-DMT-2'-*O*-TOM-$^{15}N(3)$ uridine (compound **5**) as a colorless foam in 28 % yield (376 mg, 513 µmol).

**$^{1}$H-NMR** (400 MHz, $CDCl_3$): δ (ppm) = 7.94 (d, *J* = 8.2 Hz, 1H, H-6), 7.42 – 7.35 (m, 2H, DMT), 7.34 – 7.27 (m, 6H, DMT), 7.26 – 7.20 (m, 1H, DMT), 6.87 – 6.81 (m, 4H, DMT), 6.03 (d, *J* = 3.4 Hz, 1H, H-1'), 5.29 (dd, *J* = 8.2, 3.0 Hz, 1H, H-5), 5.22 (d, *J* = 4.8 Hz, 1H, $OCH_2O$), 5.03 (d, *J* = 4.8 Hz, 1H, $OCH_2O$), 4.50 - 4.43 (m, 1H, H-3'), 4.26 (dd, *J* = 5.0, 3.4 Hz, 1H, H-2'), 4.14 – 4.06 (m, 1H, H-4'), 3.80 (s, 6H, $OCH_3$ DMT), 3.54 - 3.47 (m, 2H, H-5', H-5''), 3.19 (s, 1H, C3'-OH), 1.10 – 1.04 (m, 24H, $Si(CH(CH_3)_2)_3$).

**$^{13}$C NMR** (101 MHz, $CDCl_3$): δ (ppm) = 163.13 (d, *J* = 9.2 Hz, C4), 158.82 ($C_q$-DMT), 158.80 ($C_q$-DMT), 150.21 (d, *J* = 18.0 Hz, C2), 144.44 ($C_q$-DMT), 140.29 (C6), 135.43($C_q$-DMT), 135.16 ($C_q$-DMT), 130.31 (DMT), 130.25 (DMT), 128.27 (DMT), 128.16 (DMT), 127.29 (DMT), 113.42 (DMT), 113.40 (DMT), 102.34 (d, *J* = 6.9 Hz, C5), 90.80 ($OCH_2O$), 87.94 (C1'), 87.24 ($C_q$-DMT), 83.79 (C4'), 83.07 (C2'), 69.51 (C3'), 62.23 (C5'), 55.38 ($OCH_3$), 17.92 ($Si(CH(CH_3)_2)_3$), 17.89 ($Si(CH(CH_3)_2)_3$), 12.02 ($Si(CH(CH_3)_2)_3$), 11.97 ($Si(CH(CH_3)_2)_3$).

**HR-MS (ESI+)**: Exact mass calculated for $C_{40}H_{52}NaN^{15}NO_9Si$ $[M+Na]^+$:756.33046, found: 756.33160

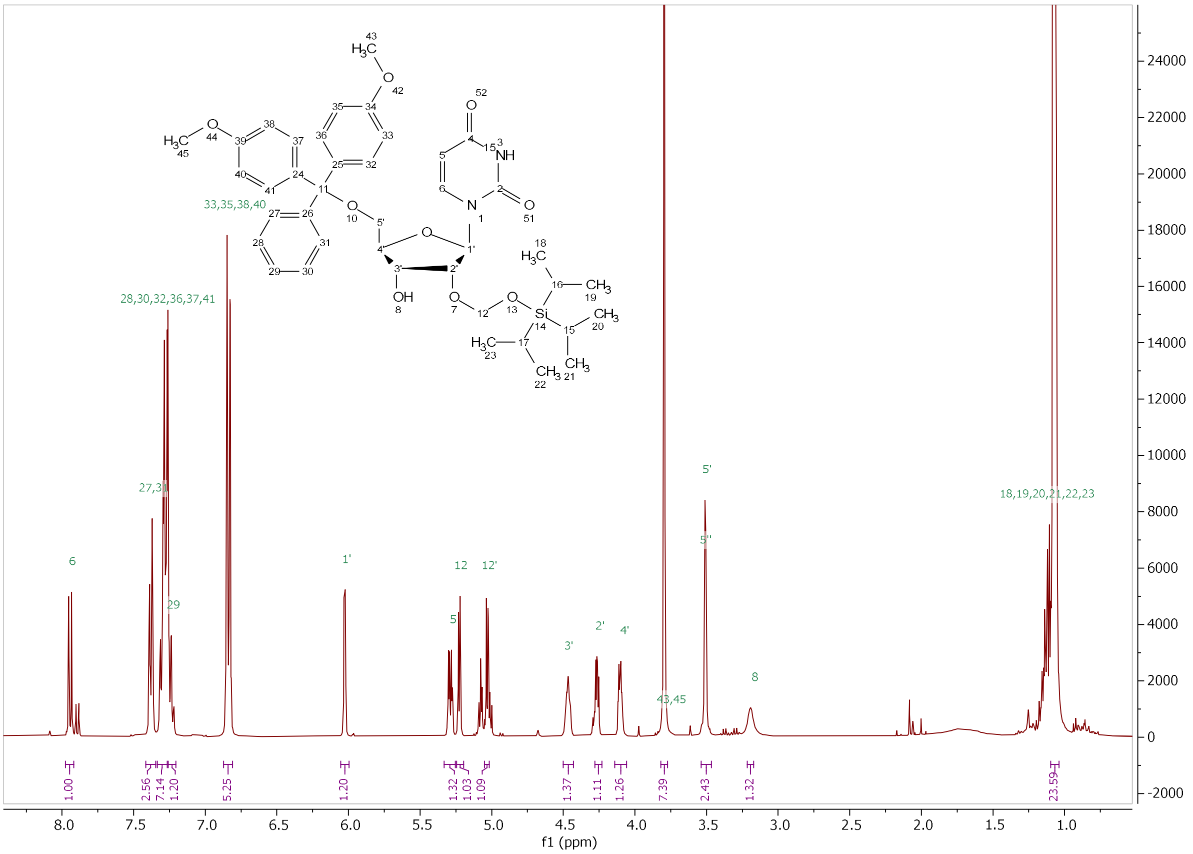

$^{1}$H-NMR (400 MHz, CDCl$_3$) of compound **5**.

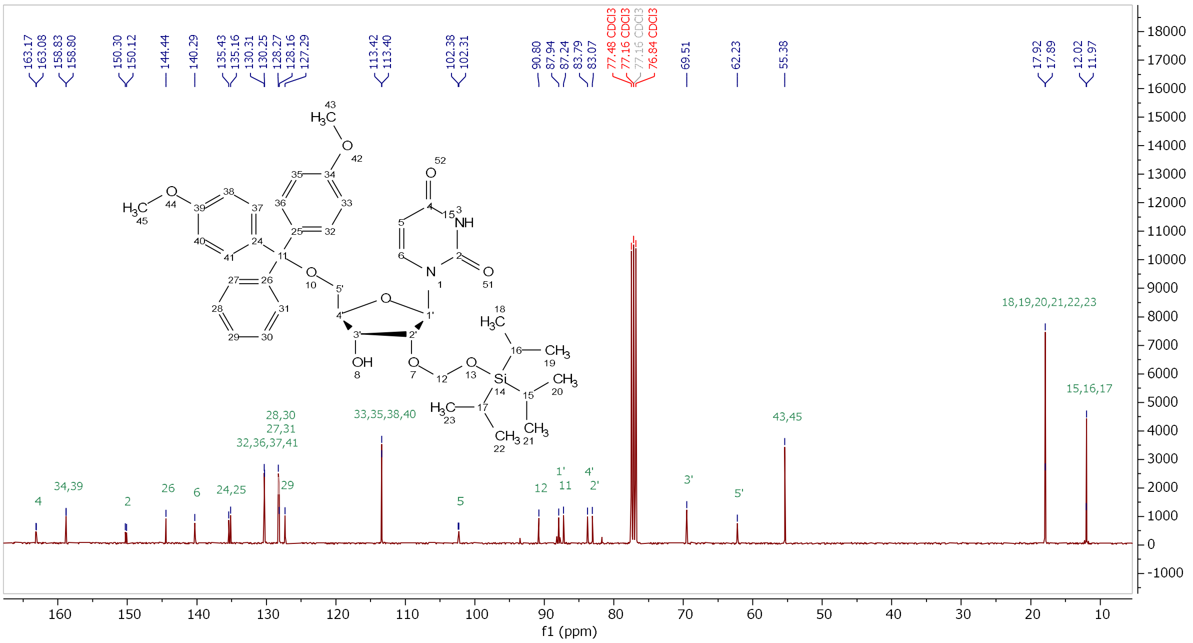

$^{13}$C-NMR (101 MHz, CDCl$_3$) of compound **5**.

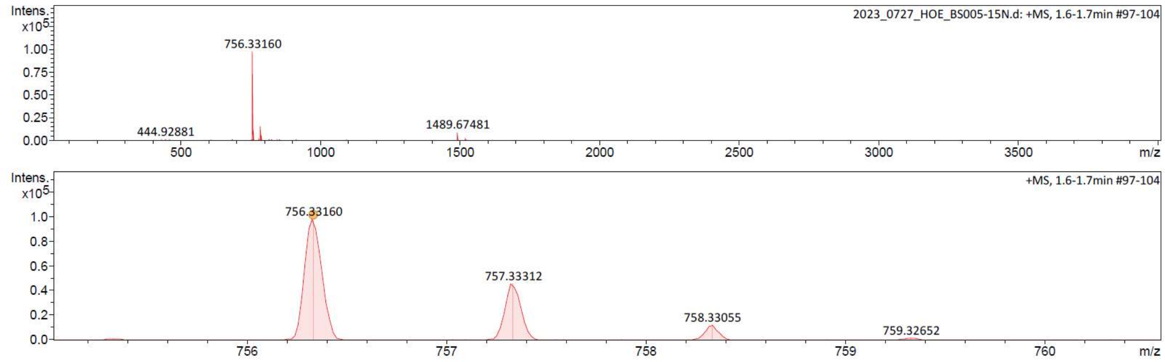


| Meas. m/z | # | Ion Formula | m/z | err [ppm] | mSigma | # mSigma | Score | rdb | e¯ Conf | N-Rule |
|---|---|---|---|---|---|---|---|---|---|---|
| 756.33160 | 1 | C40H52NNaO9Si^15N | 756.33046 | -1.51 | 28.7 | 1 | 100.00 | 16.5 | even | - |

ESI-MS of compound **5**.

**Compound 6**

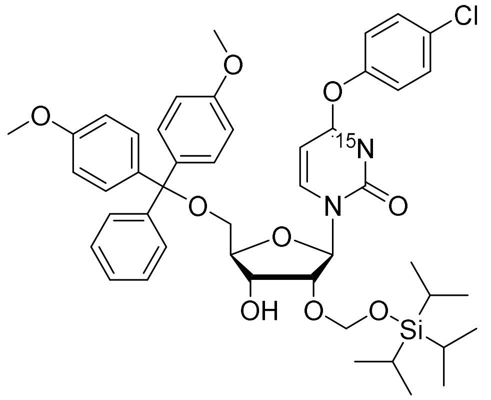


5’-*O*-DMT-2’-*O*-TOM-$^{15}$N(3) uridine (compound **5**, 377 mg, 514 µmol, 1.00 eq.) was dissolved in dry DCM (3 mL) and cooled to 0°C. DMAP (9.4 mg, 77.1 µmol, 0.15 eq.), $NEt_3$ (644 µL, 462 µmol, 9.00 eq.) and 2,4,6-triisopropylbenzesulfonyl chloride (262 mg, 866 µmol, 1.69 eq.) were added. The solution was stirred at 0 °C for 10 min, the mixture was allowed to warm up to room temperature and stirred for 2 h. The reaction mixture was diluted with DCM (30 mL), washed with $NaHCO_3$ (1 x 30 mL), dried over $Na_2SO_4$ and evaporated under reduced pressure. The crude product was directly used for the next step. Crude 5’-*O*-DMT-2’-*O*-TOM-O4-trisyl-$^{15}$N N3 uridine was dissolved in DCM (2 mL) and 4-Chlorophenol (422 mg, 3.28 µmol, 6.38 eq.) and N,N-Dimethylethylamine (835 µL, 7.71 mmol, 15.0 eq.) were added. DBU (76.7 µL, 514 mmol, 1.00 eq.) was added in three portions over 5 min and the resulting mixture was stirred for 90 min. The mixture was diluted with DCM (30 mL) and washed with saturated aqueous $NaHCO_3$ (30 mL), dried over $Na_2SO_4$ and evaporated under reduced pressure. The crude product was purified by column chromatography (n-Hex/EA + 1% $NEt_3$) to give the product 5′-*O*-DMT-2′-*O*-TOM-*O*4-chlorophenyl-$^{15}$N(3) uridine (352 mg, 417 µmol, compound **6**) as a colorless foam in 82 % yield.

**$^1$H-NMR** (400 MHz, $CDCl_3$): δ (ppm) = 8.51 (d, *J* = 7.4 Hz, 1H, H-6), 7.45 – 7.38 (m, 2H, DMT), 7.38 – 7.27 (m, 8H, DMT, chlorophenyl), 7.25 – 7.22 (m, 1H, DMT), 7.11 – 7.05 (m, 2H, chlorophenyl), 6.90 – 6.82 (m, 4H, DMT), 5.94 (s, 1H, H-1'), 5.64 (d, *J* = 7.4 Hz, 1H, H-5), 5.27 (d, *J* = 4.7 Hz, 1H, $OCH_2O$), 5.11 (d, *J* = 4.7 Hz, 1H, $OCH_2O$), 4.47-4.37 (m, 1H, H-3'), 4.21 (d, *J* = 4.9 Hz, 1H, H-2'), 4.11 – 4.03 (m, 1H, H-4'), 3.83 - 3.78 (m, 6H, $OCH_3$ DMT), 3.65 - 3.51 (m, 2H, H-5’, H-5''), 3.35 (d, *J* = 8.5 Hz, 1H, C3’-OH), 1.08 – 1.00 (m, 19H, $Si(CH(CH_3)_2)_3$).

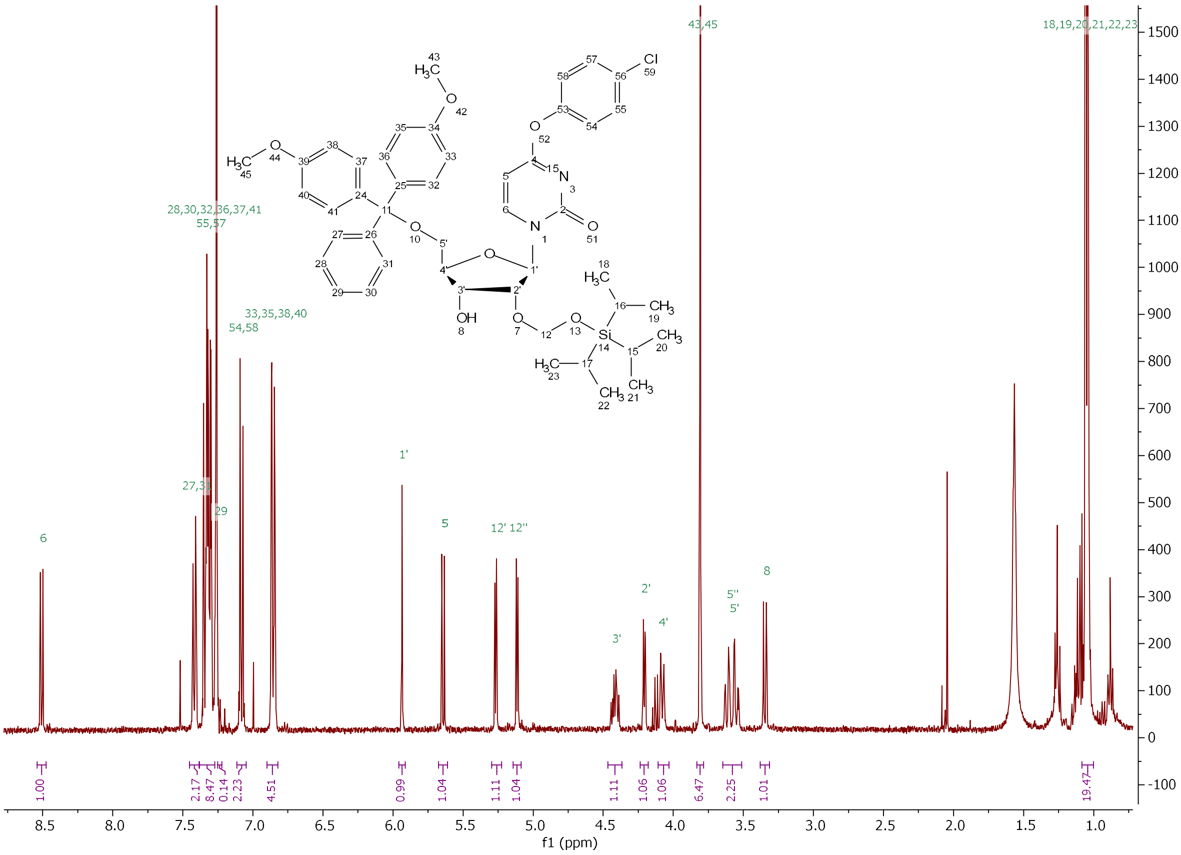


$^1$H-NMR (400 MHz, $CDCl_3$) of compound **6**.

**Compound 7**

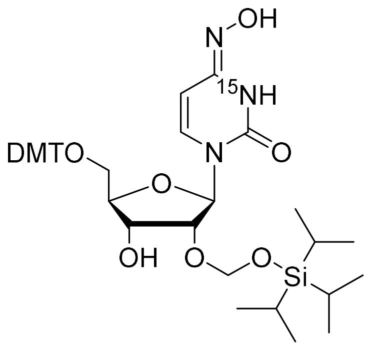


5′-*O*-DMT-2′-*O*-TOM-*O*4-chlorophenyl-$^{15}N(3)$ uridine (compound **6**, 332 mg, 393 µmol, 1.00 eq.) was dissolved in dry $CH_3CN$ (5 mL) under a nitrogen atmosphere. DMAP (146 mg, 1.20 µmol, 3.04 eq.) and $NEt_3$ (274 µL, 1.97 µmol, 5.00 eq.) were added, followed by hydroxylamine hydrochloride (136 mg, 1.93 mmol, 4.90 eq.). After stirring for 21 h at ambient temperature, 35 mg of hydroxylamine hydrochloride (0.50 mmol, 1.27 eq.) and 60 µL of $NEt_3$ (0.43 µmol, 1.09 eq.) were added. After stirring 24 h, the reaction mixture was diluted with DCM (20 mL) and washed with saturated aqueous $NaHCO_3$ (2 x 20 mL). The organic phase was dried over $Na_2SO_4$ and evaporated under reduced pressure. The crude product was purified by column chromatography (n-Hex:EA to 1:1 to 1:2 + 1% $NEt_3$) to yield the product 5'-*O*-DMT-$N^4$-hydroxy-2'-*O*-TOM- $^{15}N(3)$ cytidine (compound **7**, 172 mg, 230 µmol, 58 %) as colorless foam.

**$^1$H-NMR** (400 MHz, $CDCl_3$): δ (ppm) = 8.66 (d, *J* = 94.8 Hz, 1H, H-3), 7.42 – 7.35 (m, 2H, DMT), 7.32 – 7.26 (m, 6H, DMT), 7.25 – 7.20 (m, 1H, DMT), 7.15 (d, *J* = 8.2 Hz, 1H, H-6), 6.88 – 6.79 (m, 4H, DMT), 6.02 (d, *J* = 4.4 Hz, 1H, H-1'), 5.25 (dd, *J* = 8.2, 3.0 Hz, 1H, H-5), 5.21(d, *J* = 4.7 Hz, 1H, $OCH_2O$), 5.00 (d, *J* = 4.7 Hz, 1H, $OCH_2O$), 4.45 – 4.38 (m, 1H, H-3'), 4.23 (dd, *J* = 4.8 Hz, 1H, H-2'), 4.12 – 4.05 (m, 1H, H-4'), 3.80 (s, 6H, $OCH_3$ DMT), 3.50 – 3.38 (m, 2H, H-5'', H-5'), 3.13 (s, 1H, C3'-OH), 1.13 – 1.01 (m, 18H, $Si(CH(CH_3)_2)_3$).

**$^{13}$C NMR** (101 MHz, $CDCl_3$): δ (ppm) = 158.68 ($C_q$-DMT), 158.66 ($C_q$-DMT), 149.70 (d, *J* = 21.5 Hz, C2), 145.21 (d, *J* = 11.4 Hz, C4), 144.40 ($C_q$-DMT), 135.80 ($C_q$-DMT), 135.50 ($C_q$-DMT), 130.53 (C6), 130.33 (DMT), 130.28 (DMT), 128.37 (DMT), 128.09 (DMT), 127.18 (DMT), 113.39 (DMT), 98.58 (d, broad, C5), 90.78 ($OCH_2O$), 87.05 (C1', $C_q$-DMT), 83.45 (C4'), 82.32 (C2'), 70.22 (C3'), 62.92 (C5'), 55.44 ($OCH_3$), 55.43 ($OCH_3$), 17.94 ($Si(CH(CH_3)_2)_3$), 11.98 ($Si(CH(CH_3)_2)_3$).

**$^1$H-NMR** (400 MHz, $d_6$-DMSO): *δ* (ppm) = 10.03 (s, N4-OH), 9.54 (dd, *J* = 94.6, 2.6 Hz, H-3), 7.41 – 7.34 (m, 2H, DMT), 7.34 – 7.17 (m, 7H, DMT), 6.93 – 6.87 (m, 4H, DMT), 6.86 (d, *J* = 8.2 Hz, 1H, H-6), 5.87 (d, *J* = 6.2 Hz, 1H, H-1'), 5.37 (dd, *J* = 8.2, 2.8 Hz, 1H, H-5), 5.15 (d, *J* = 5.6 Hz, C3'-OH), 4.95 (d, *J* = 5.3 Hz, 1H, $OCH_2O$), 4.89 (d, *J* = 5.3 Hz, 1H, $OCH_2O$), 4.24 (dd, *J* = 5.8 Hz, 1H, H-2'), 4.07 (dd, *J* = 5.5, 3.9 Hz, 1H, H-3'), 3.95 – 3.88 (m, 1H, H-4'), 3.73 (s, 6H, $OCH_3$ DMT), 3.25 – 3.12 (m, 2H, H-5'', H-5'''), 1.01 – 0.94 (m, 18H, $Si(CH(CH_3)_2)_3$).

**$^{13}$C-NMR** (101 MHz, $d_6$-DMSO): *δ* (ppm) = 158.18 ($C_q$-DMT), 149.22 (broad, C2), 144.72 ($C_q$-DMT), 143.04 (broad, C4), 135.48 ($C_q$-DMT), 135.39 ($C_q$-DMT), 129.83 (DMT, C6), 127.98 (DMT), 127.78 (DMT), 126.86 (DMT), 113.30 (DMT), 98.81 (broad, C5), 88.34 ($OCH_2O$), 86.02 ($C_q$-DMT), 85.73 (C1'), 83.11 (C4'), 76.23 (C2'), 68.86 (C3'), 63.71 (C5'), 55.11 ($OCH_3$), 17.71 ($Si(CH(CH_3)_2)_3$), 17.70 ($Si(CH(CH_3)_2)_3$), 11.44 ($Si(CH(CH_3)_2)_3$).

**$^{15}$N-NMR** (41 MHz, $d_6$-DMSO): *δ* (ppm) = 118.3 (N3).

**HR-MS (ESI+)**: Exact mass calculated for $C_{40}H_{53}NaN_2{}^{15}NO_9Si$ $[M+Na]^+$:771.34136, found: 771.34207

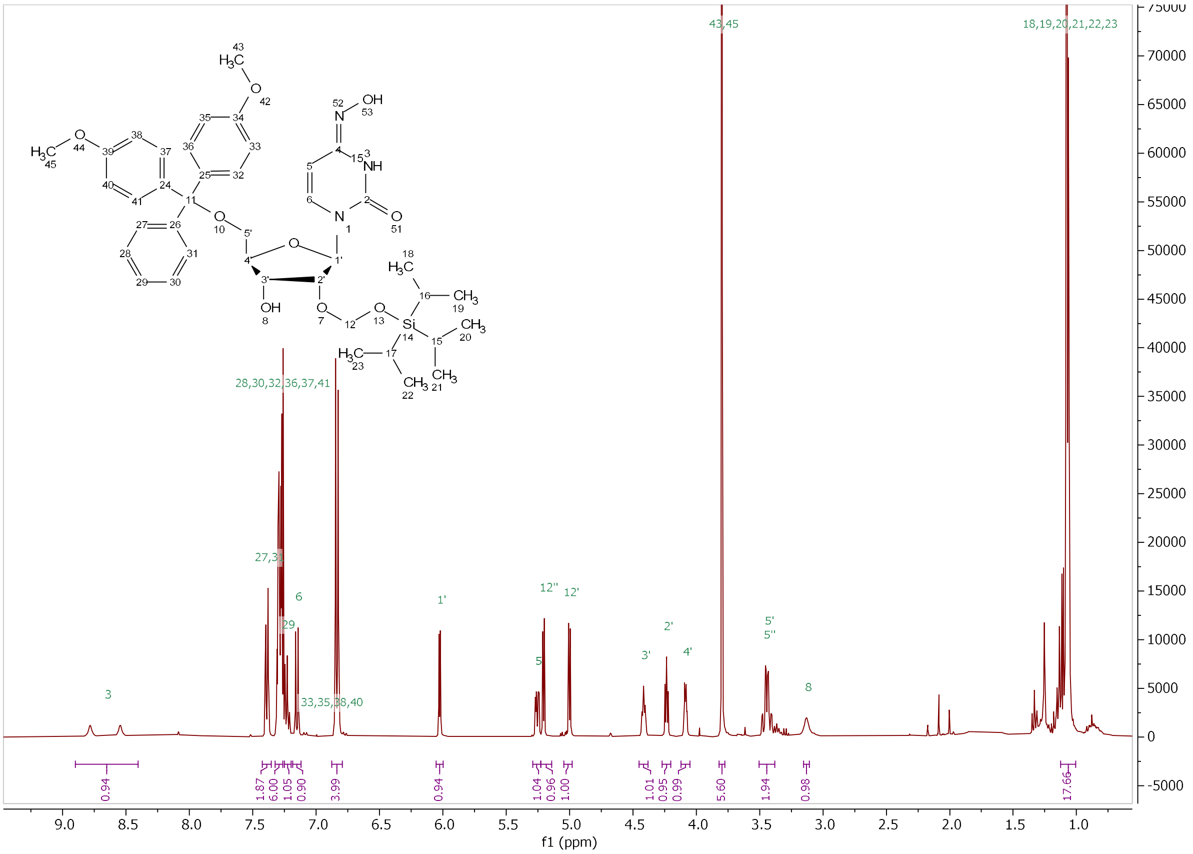

$^{1}$H-NMR (400 MHz, $CDCl_3$) of compound **7**.

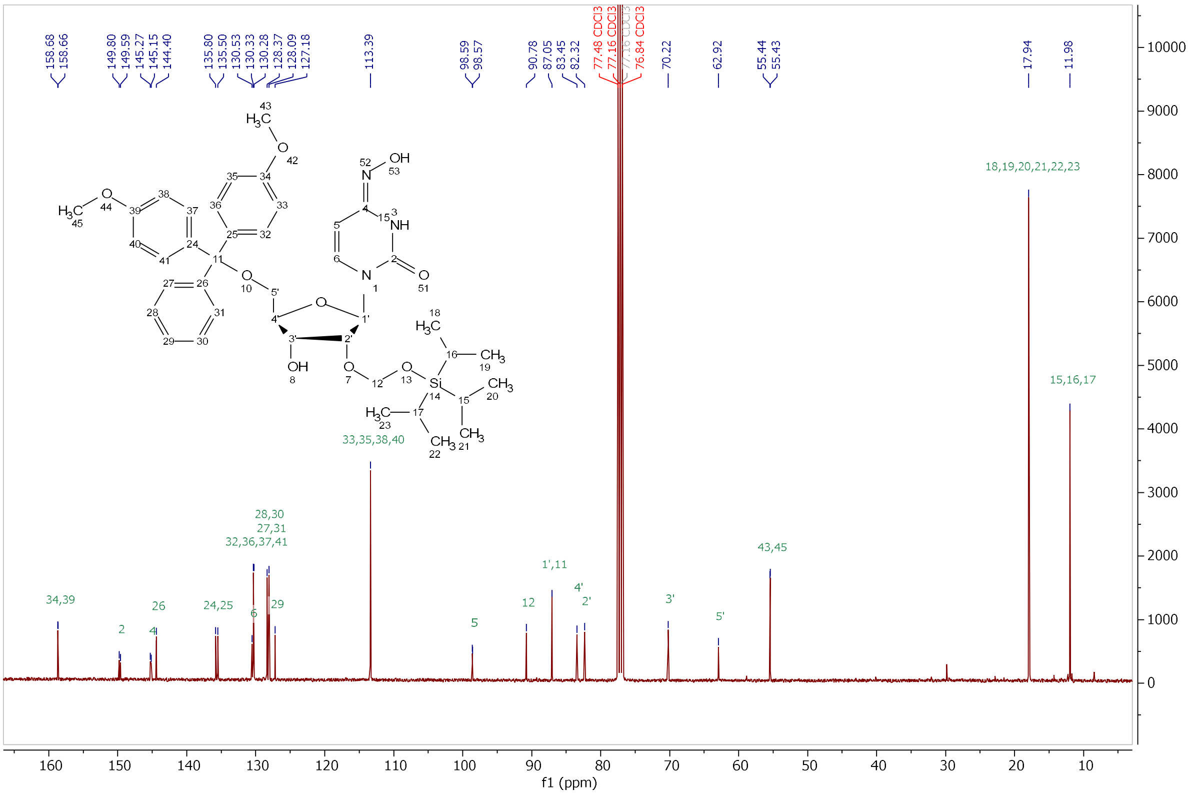

$^{13}$C-NMR (101 MHz, $CDCl_3$) of compound **7**.

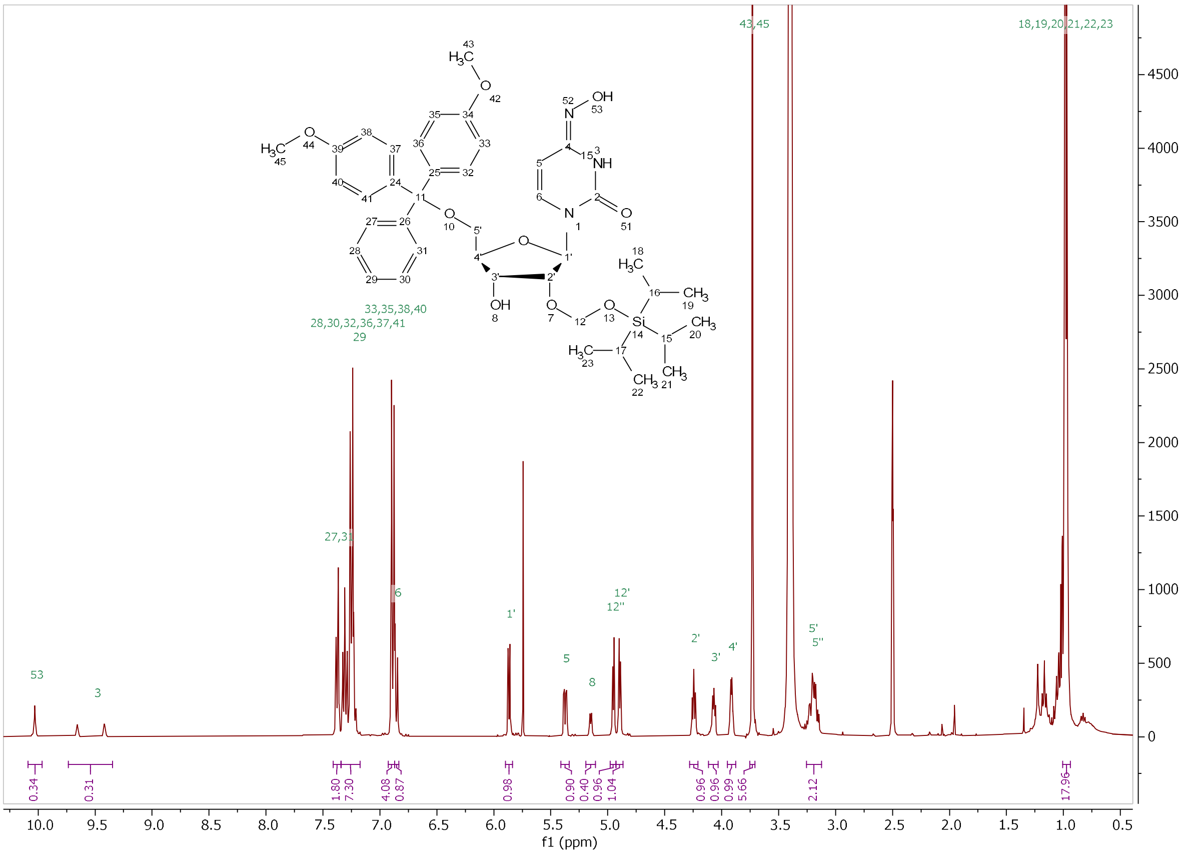

$^{1}$H-NMR (400 MHz, d6-DMSO) of compound **7**.

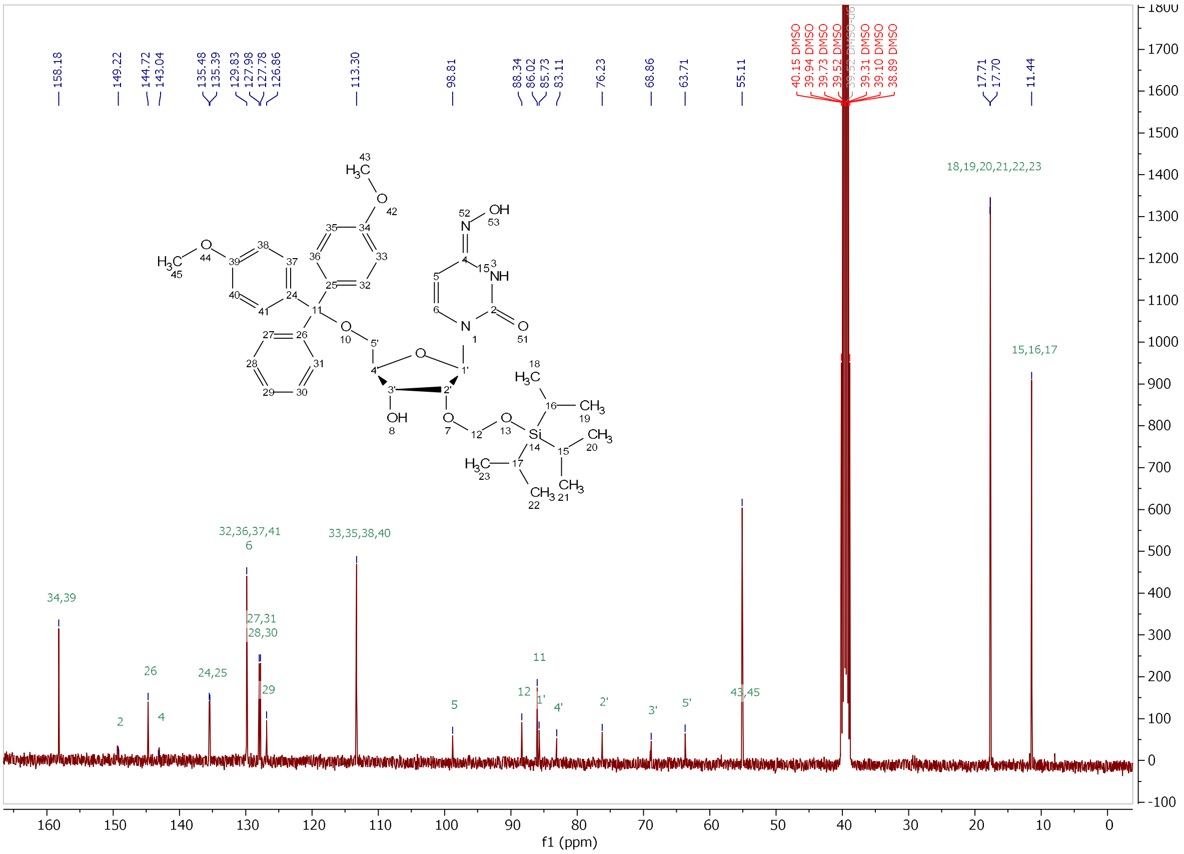

$^{13}$C-NMR (101 MHz, d6-DMSO) of compound **7**.

# DMT-TOM-$^{15}$N(3)-NHC (comp. 7)

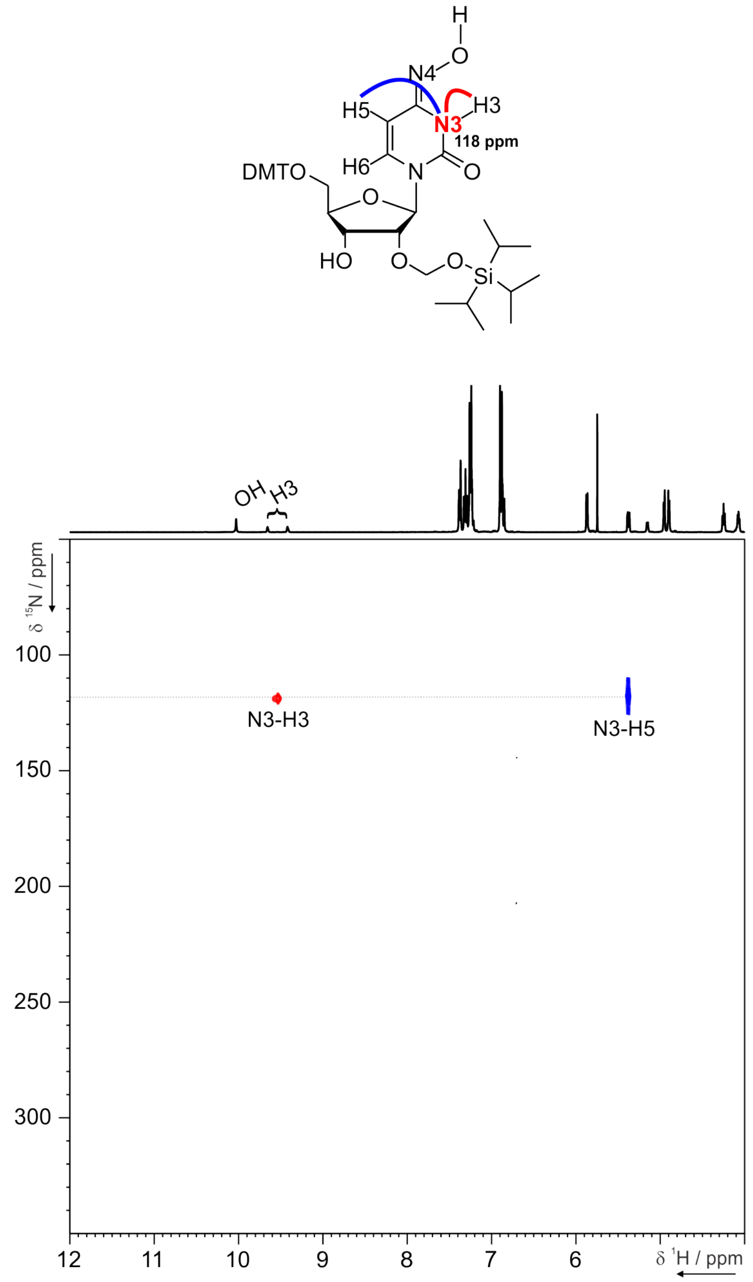


Overlay of 2D $^{1}$H,$^{15}$N-HSQC (red) and 2D $^{1}$H,$^{15}$N-HMBC (blue) of compound **7** in 100% $d_6$-DMSO, 400 MHz, room temperature. Correlations detected in the spectra are marked on the structure with the corresponding color code. Only the imino form is observed.

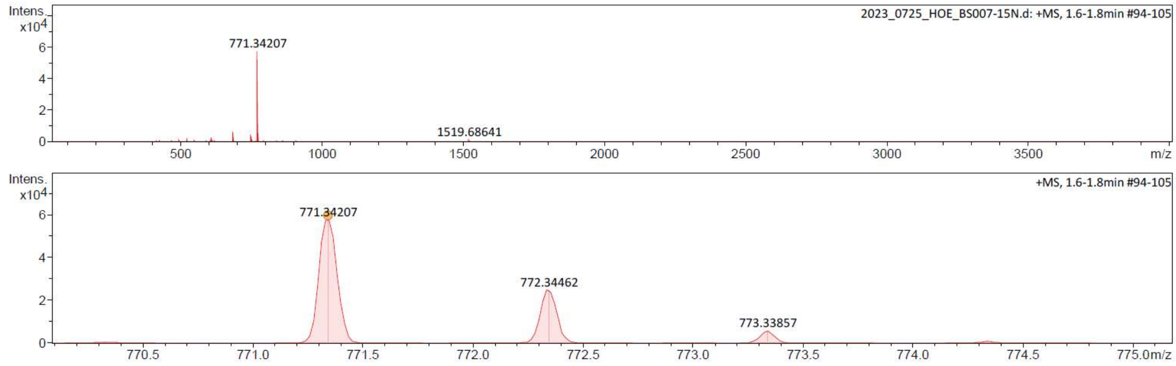


| Meas. m/z | # | Ion Formula | m/z | err [ppm] | mSigma | # mSigma | Score | rdb | e⁻ Conf | N-Rule |
|---|---|---|---|---|---|---|---|---|---|---|
| 771.34207 | 1 | C40H53N2NaO9Si^15N | 771.34136 | -0.92 | 46.3 | 1 | 100.00 | 16.5 | even | - |

ESI-MS of compound **7**.

**Compound 8**

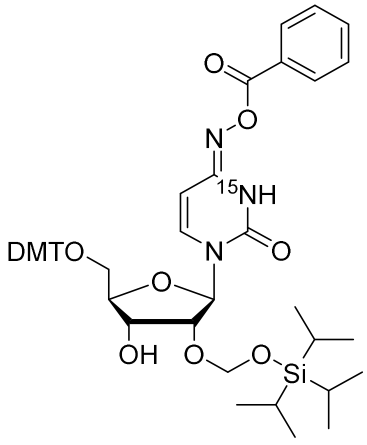

5'-*O*-DMT-*N*[4]-hydroxy-2'-*O*-TOM- $^{15}$N(3) cytidine (compound **7**, 163 mg, 218 µmol, 1.00 eq.) was dissolved in dry DCM (4 mL). DMAP (54.1 mg, 443 µmol, 2.03 eq.) and $Net_3$ (122 µL, 873 µmol, 4.00 eq.) were added. Benzoic anhydride (49.1 mg, 217 µmol, 0.99 eq.) was added in 4 portions within 2 h and the resulting mixture was stirred for 2 h at room temperature. Benzoic anhydride was added (2 mg) and the reaction mixture was stirred for 30 min. All volatiles were removed under reduced pressure. The crude residue was purified by column chromatography (n-Hex:EA + 1% $NEt_3$ (4:1 to 3:1 to 2:1 to 1:1 to 1:2) to yield the product *N*[4]-*O*-benzoyl-5'-*O*-DMT-*N*[4]-hydroxy-2'-*O*-TOM- $^{15}$N(3) cytidine (compound **8**, 120 mg, 141 µmol, 73%) as a colorless foam.

**$^1$H-NMR** (400 MHz, $CDCl_3$): δ (ppm) = 8.20 (dd, *J* = 94.7, 2.5 Hz, 1H), 8.06 – 8.01 (m, 2H, DMT), 7.65 – 7.56 (m, 1H, bz), 7.52 – 7.46 (m, 2H, bz), 7.44 (d, *J* = 8.3 Hz, H-6), 7.41 – 7.35 (m, 2H, DMT), 7.33 – 7.26 (m, 6H, DMT), 7.25 – 7.20 (m, 1H, DMT), 6.89 – 6.79 (m, 4H, DMT), 6.04 (d, *J* = 4.7 Hz, 1H, H-1'), 5.49 (dt, *J* = 8.3, 2.6 Hz, 1H, H-5), 5.21 (d, *J* = 4.8 Hz, 1H, $OCH_2O$), 5.00 (d, *J* = 4.8 Hz, 1H, $OCH_2O$), 4.51 – 4.43 (m, 1H, H-3'), 4.28 (dd, *J* = 4.9 Hz, 1H, H-2'), 4.15 – 4.09 (m, 1H, H-4'), 3.80 (s, 6H, $OCH_3$ DMT), 3.49 – 3.44 (m, 2H, H-5', H-5''), 3.10 (d, *J* = 4.4 Hz, 1H, C3'-OH), 1.10 – 1.03 (m, 19H, $Si(CH(CH_3)_2)_3$).

**$^{13}$C-NMR** (101 MHz, $CDCl_3$): δ (ppm) = 163.51 (C(O)bz), 158.83 ($C_q$-DMT), 158.80 ($C_q$-DMT), 149.54 (d, *J* = 11.3 Hz, C4), 148.48 (d, *J* = 20.9 Hz, C2), 144.45 ($C_q$-DMT), 135.36 ($C_q$-DMT), 135.09 ($C_q$-DMT), 133.83 (C6), 133.63 (bz), 130.29 (DMT), 130.22 (DMT), 129.72 (bz), 128.79 (bz), 128.21 (DMT), 128.13 (DMT), 127.32 (DMT), 113.40 (DMT), 113.39 (DMT), 97.48 (broad, C5), 90.84 ($OCH_2O$), 87.27 ($C_q$-DMT), 87.04 (C1'), 83.85 (C4'), 82.53 (C2'), 70.44 (C3'), 62.92 (C5'), 55.37 ($OCH_3$), 17.93 ($Si(CH(CH_3)_2)_3$), 11.97 ($Si(CH(CH_3)_2)_3$).

**HR-MS (ESI+)**: Exact mass calculated for $C_{47}H_{57}NaN_2{}^{15}NO_{10}Si$ $[M+Na]^+$:875.36759, found: 875.36831

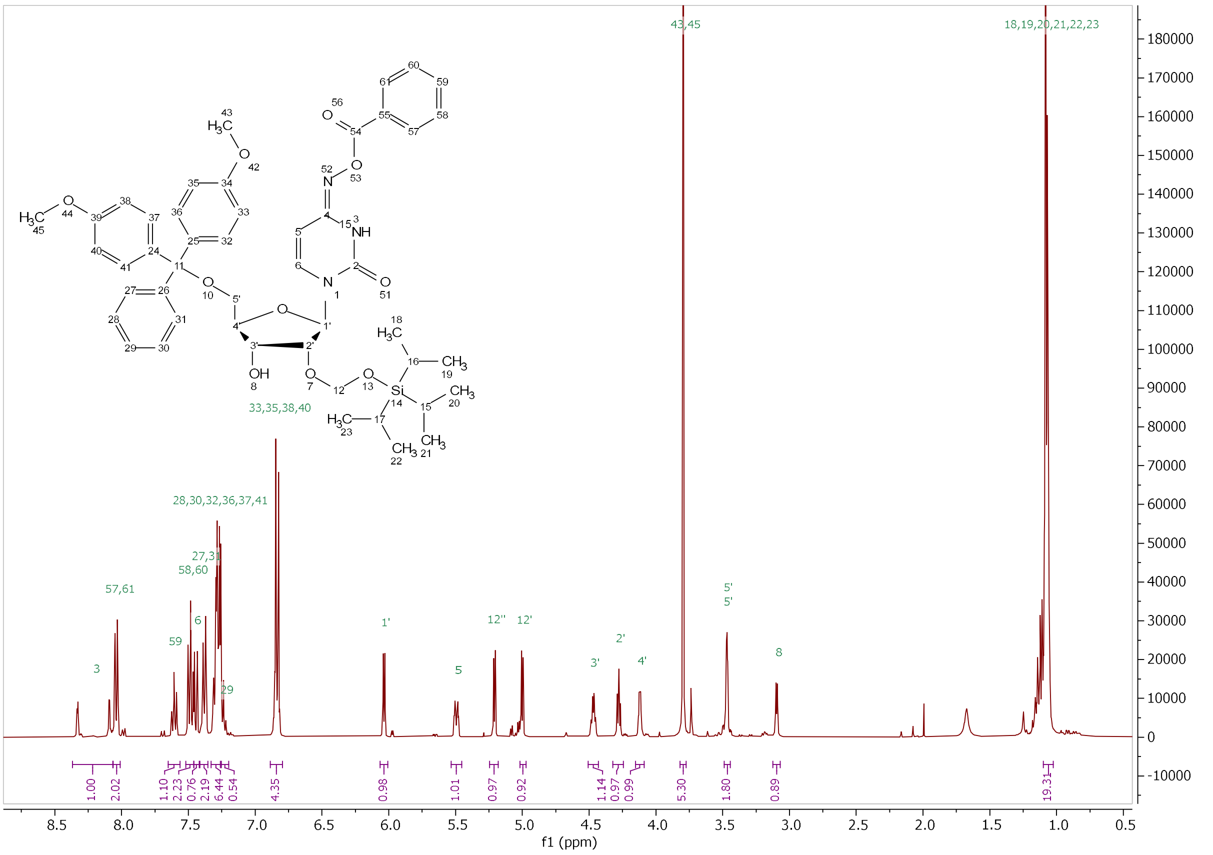

$^1$H-NMR (400 MHz, $CDCl_3$) of compound **8**.

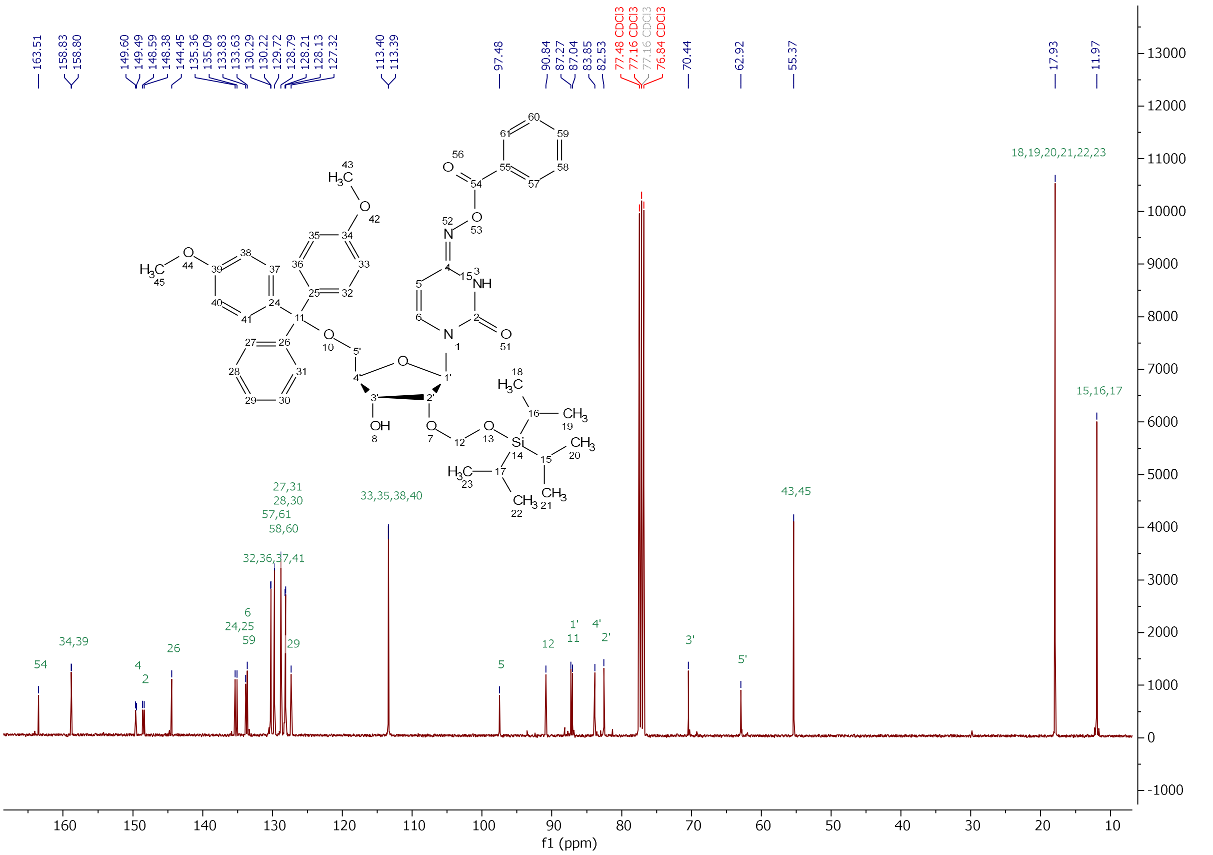

$^{13}$C-NMR (101 MHz, $CDCl_3$) of compound **8**.

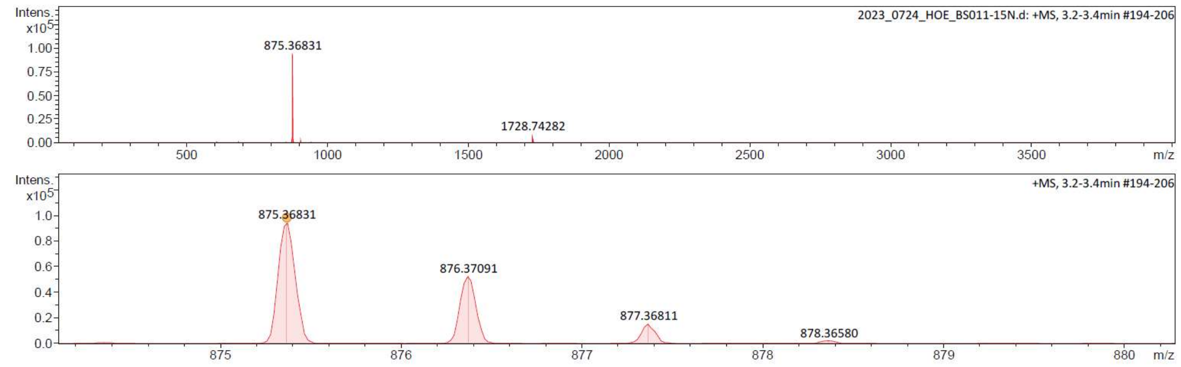


| Meas. m/z | # | Ion Formula | m/z | err [ppm] | mSigma | # mSigma | Score | rdb | e⁻ Conf | N-Rule |
|---|---|---|---|---|---|---|---|---|---|---|
| 875.36831 | 1 | C47H57N2NaO10Si^15N | 875.36758 | -0.84 | 30.4 | 1 | 100.00 | 21.5 | even | - |

ESI-MS of coumpound **8**.

**Compound 9**

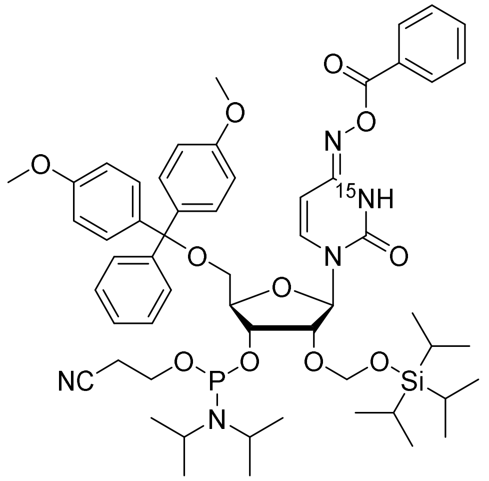

$N^4$-Benzoyl-5'-$O$-DMT-$N^4$-hydroxy-2'-$O$-TOM-$^{15}$N(3) cytidine (compound **8**, 31.9 mg, 37.4 µmol, 1.00 eq.) was dissolved in dry DCM (1 mL) and cooled to 0 °C. 4,5-dicyanoimidazol (DCI, 3.10 mg, 26.3 µmol, 0.70 eq.) was added followed by 2-Cyanoethyl *N*,*N*,*N'*,*N'*-tetraisopropyl phosphoramidite (7.00 µL, 7.00 µmol, 0.59 eq.). After 1 h, 4,5-dicyanoimidazol (DCI, 3.10 mg, 26.3 µmol, 0.70 eq.) was added followed by 2-Cyanoethyl *N*,*N*,*N'*,*N'*-tetraisopropyl phosphoramidite (7.00 µL, 7.00 µmol, 0.59 eq.) were added. The mixture was stirred for 16 h while the reaction mixture was allowed to warm to room temperature, before all the solvents were removed under reduced pressure. The crude product was purified by column chromatography (cy/EA 4:1 to 3:1 to 2:1 + 1% $NEt_3$) to yield $N^4$-$O$-Benzoyl-5'-$O$-DMT-$N^4$-hydroxy-2'-TOM-$^{15}$N(3) cytidine 3'-cyanoethyl-N,N-diisopropylphosphoramidite (compound **9**, 15.5 mg, 14.7 µmol, 40%) as a colorless foam.

**$^1$H-NMR** (400 MHz, $CDCl_3$): δ (ppm) = 8.07 – 8.01 (m, 4H, bz, 2 diast.), 7.66 – 7.56 (m, 2H, bz, 2 diast.), 7.54 – 7.45 (m, 4H, bz, 2 diast.), 7.45 – 7.34 (m, 5H, DMT, 2 diast.), 7.34 – 7.21 (m, 14H, DMT, 2 diast.), 6.88 – 6.80 (m, 8H, DMT, 2 diast.), 6.12 (d, $J$ = 5.8 Hz, H-1', 1 diast.), 6.10 (d, $J$ = 4.4 Hz, H-1', 1 diast.), 5.59 (dd, $J$ = 8.4, 3.0 Hz, 1H, H-5, 1 diast.), 5.54 (dd, $J$ = 8.2, 3.0 Hz, 1H, H-5, 1 diast.), 5.08 – 4.90 (m, 4H, $OCH_2O$, 2 diast.), 4.54 – 4.44 (m, 1H, H-2'), 4.43 – 4.34 (m, 2H, H-3', 2 diast.), 4.30 - 4.21 (m, 1H, H-4', 1 diast.), 4.21 - 4.10 (m, 1H, H-4', 1 diast.), 4.00 – 3.80 (m, 1H, $CNCH_2$), 3.80 – 3.78 (m, 11H, $OCH_3$, 2 diast.), 3.77 – 3.72 (m, 4H, $CNCH_2$, 2 diast.), 3.70 – 3.30 (m, 7H, H-5'', H-5''', $NCH(CH_3)_2$, 2 diast.), 2.72 – 2.57 (m, 1H, $POCH_2$), 2.48 – 2.31 (m, 2H, $POCH_2$, 2 diast.), 1.21 – 1.14 (m, 15H, $NCH(CH_3)_2$, 2 diast.), 1.07 – 1.00 (m, 45H, $Si(CH(CH_3)_2)_3$, 2 diast.).

**$^{31}$P-NMR** (162 MHz, $CDCl_3$): δ (ppm) = 150.60, 150.12.

**MS (ESI+)**: Exact mass calculated for $C_{56}H_{74}NaN_4{}^{15}NO_{11}PSi$ $[M+Na]^+$:1075.47543, found: 1075.47517

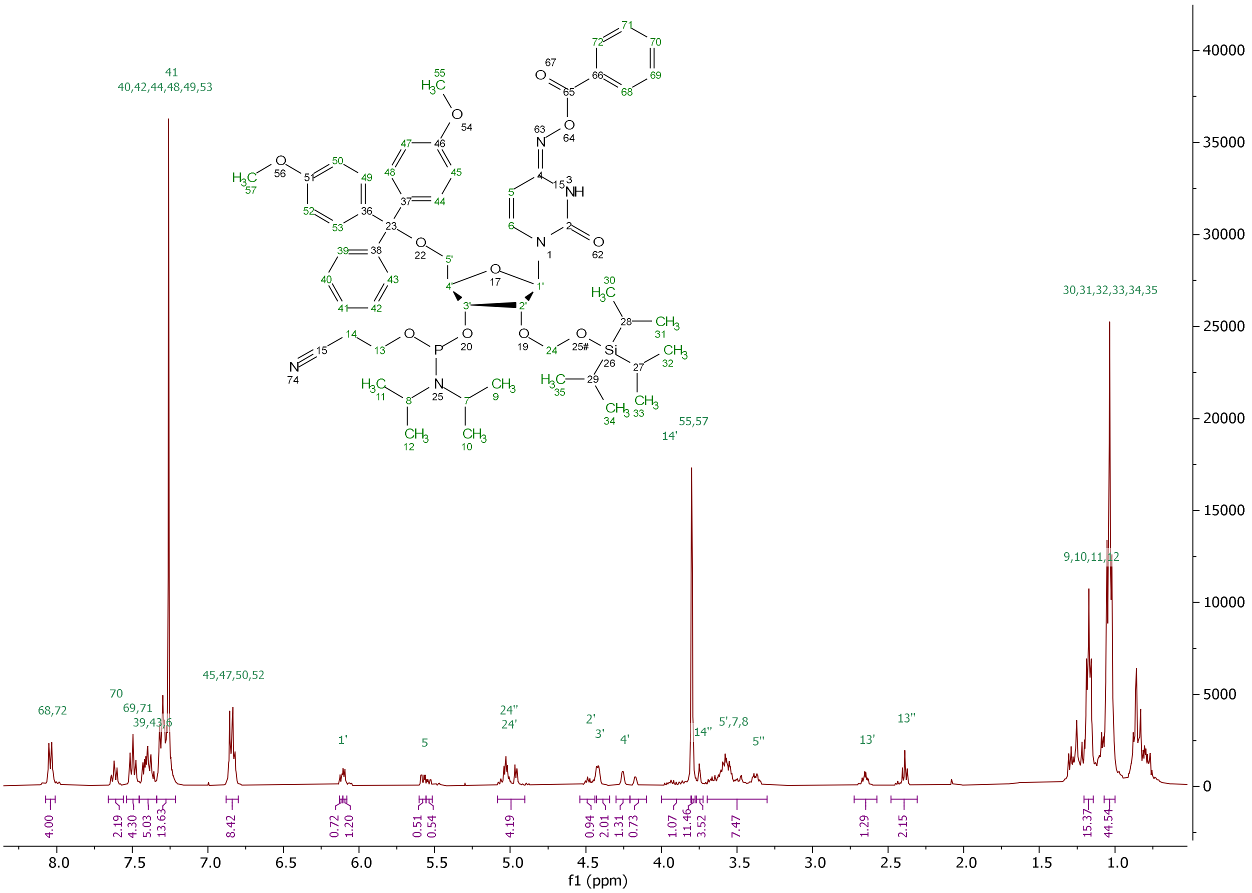

$^1H$-NMR (400 MHz, $CDCl_3$) of compound **9**.

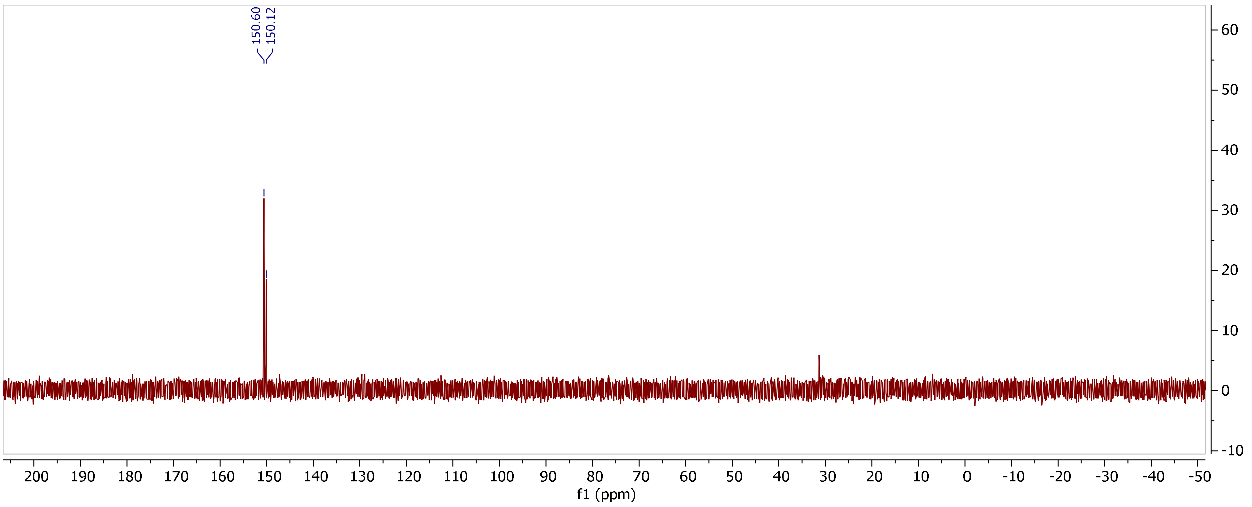

$^{31}P$-NMR (162 MHz, $CDCl_3$) of compound **9**.

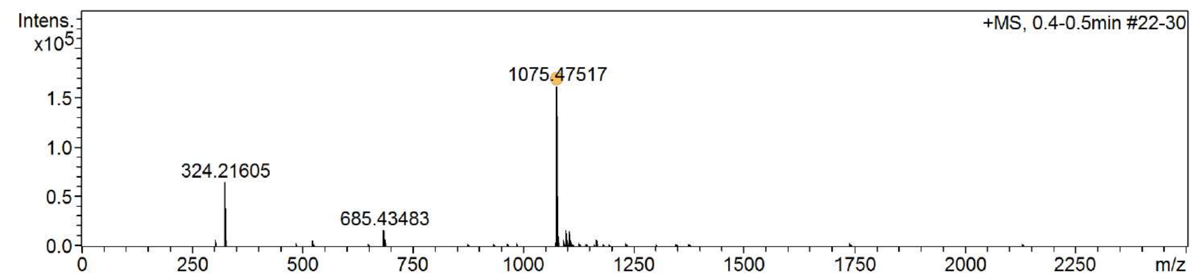


| Meas. m/z | # | Ion Formula | m/z | err [ppm] | mSigma | # mSigma | Score | rdb | e⁻ Conf | N-Rule |
|---|---|---|---|---|---|---|---|---|---|---|
| 1075.47517 | 1 | C56H74N4NaO11PSi^15N | 1075.47543 | 0.24 | 17.1 | 1 | 100.00 | 23.5 | even | - |

ESI-MS of compound **9**.

**Native gel electrophoresis**

Native gel electrophoresis was performed on a native 20% polyacrylamide (PAA) gel (1 x 80 x 100 mm) in TBE buffer. The 6 μM RNA samples were annealed either with slow cooling (2 min at 95 °C followed by slow cooling to room temperature) or with snap cooling (2 min at 95 °C followed by rapid cooling on ice).

3 μL of the annealed samples were mixed with 3 μL of loading buffer (50% v/v glycerol) and loaded on the gel. The running buffer contained TBE buffer. The gel was run 1 h at a constant voltage of 100 V and room temperature. Finally, the gel was stained with 1x SYBR Gold in TBE buffer and imaged on a ChemiDoc system.

**UV melting curves fitting and Van't Hoff analysis**

Thermal melting curves were recorded on a Varian Cary 100 spectrometer equipped with a Peltier temperature controller.

Samples were prepared in 10 mM sodium phosphate buffer (pH 7.0) containing 100 mM NaCl and supplied in quartz cuvettes. 300 µL of samples with 20 µM and 10 µM duplex concentration were filled in macro cuvettes (Hellma®) with 0.1 cm path length, whereas the samples with of 5 µM, 2 µM and 1 µM duplex concentration were filled in semi-micro cuvettes with 1 cm path length. To prevent evaporation of the solvent, a layer of silicon oil was applied to cover the aqueous solution. Furthermore, the chamber which contained the cuvettes was purged with a constant nitrogen flow, to circumvent the condensation of the air moisture on the glass surface arising from the temperature changes. Two heating and two cooling ramps with a heating rate of 0.5 °C/min were measured in a temperature interval of 10 to 90 °C and monitored at 250, 260 and 280 nm.

The melting temperatures were obtained from the inflection point of the sigmoidal melting curves (Hyperchromicity vs Temperature) and are given as the mean values of the two heating and two cooling ramps. They were determined by fitting a two-state transition model with lower and upper baseline, assuming an accuracy of ± 0.5 °C.

For the Dickerson-Drew sequences forming duplex structures, enthalpy change ($\Delta H^0$), entropy change ($\Delta S^0$), and free energy change ($\Delta G^0$) were calculated from the analysis of the concentrantion dependence of the melting temperature $T_m$ of bimolecular association of two self-complementary strands according to Breslauer *et al.*[9]

$$\frac{1}{T_\mathrm{m}} = \frac{R}{\Delta H^0}\ln c_\mathrm{tot} + \frac{\Delta S^0}{\Delta H^0}$$

Eq. (1)

$c_{tot}$ is the total concentration of the RNA strand recalculated according to the UV absorption of the melted duplexes and $R$ is the universal gas constant. Using the van't Hoff analysis and plotting the $1/T_m$ against $\ln(c_{tot})$, $\Delta H^0$ could be determined from the slope ($m$) of the linear fit and $\Delta S^0$ from the intercept ($b$) of the linear fit:

$\Delta H^0 = R / m$

$\Delta S^0 = b \cdot \Delta H^0$

The the free energy $\Delta G^{298}$ was then calculated using the Gibbs-Helmholtz equation with $T$ = 298 K:
$\Delta G^{298}=\Delta H^0 - T\Delta S^0$

**NMR samples details**

The NMR samples used for Figure 3 (Main article), 2D and CLEANEX-PM experiments had the following duplex concentrations:
CG (R922) = 750 µM (Figures 3, S4-S5; used for CLEANEX-PM)
XG (R961) = 850 µM (Figures 3, S4-S5; used for CLEANEX-PM)
XG (R993) = 230 µM ($^{15}$N(4)-labeled NHC) (Figure 4)
UG (R976) = 700 µM (Figures 3, S4-S5; used for CLEANEX-PM)
UA (R1006) = 700 µM (Figures 3, S4-S5, S10; used for CLEANEX-PM)
XA (R1007) = 340 µM ($^{15}$N(4)-labeled NHC) (Figures 3, 5A, S3-S5, S7-S10; used for CLEANEX-PM)
XA (R1071) = 450 µM ($^{15}$N(3)-labeled NHC) (Figure 5B-C, S6)
CA (R1130) = 950 µM (Figures 3, S3-S4, S6)

**NMR CLEANEX-PM**

Water hydrogen exchange rates of imino protons ($k_{EX}$) were measured using a 1D version of the CLEANEX-PM pulse sequence,[10,11] according to the protocol described before.[12,13,14] A series of exchange experiments with different mixing times $\tau_m$ (5, 25, 50, 50, 100, 100, 150, 200, 300, 400, 400, 500 ms) were performed using the standard Bruker pulse program zgcxesgp, employing excitation sculpting with gradients as water suppression scheme.[15] The Bruker standard program zgesgp, employing as well excitation sculpting with gradients as water suppression scheme,[15] was used as reference experiment, without CLEANEX-PM element and water saturation pulse. 1024 scans were recorded for each experiment using an interscan delay of 2.0 s and 16 dummy scans. The spectra were processed using a line broadening factor of 10 Hz and the peak intensities were used for the data analysis.
The water $^1$H longitudinal relaxation rate $R_{1w}$ (s$^{-1}$) was determined for each sample using a saturation recovery experiment as described by Szulik et al.[16]
For each imino signal, the ratio between the intensity of peak in the CLEANEX-PM experiment ($I$) and the intensity of the peak in the reference experiment ($I_0$) was plotted as a function of the mixing time ($\tau_m$). The imino proton water exchange rate $k_{EX}$ (s$^{-1}$) and the apparent imino proton relaxation rate $R_{1A}$ were obtained by fitting the resulting curve to the equation (2):

$$\frac{I}{I_0} = \frac{k_{EX}}{(R_{1A} + k_{EX} - R_{1w})} * (e^{-R_{1w} * \tau_m} - e^{(-R_{1A} + k_{EX})\tau_m})$$

Eq. (2)

with:
$I$ = intensity of the imino peak at the mixing time $\tau_m$
$I_0$ = intensity of the imino peak in the reference experiment

$k_{EX}$ = imino proton to water exchange rate ($s^{-1}$)
$\tau_m$ = mixing time (s)
$R_{1w}$ = water $^1H$ longitudinal relaxation rate ($s^{-1}$)
$R_{1A}$ = imino $^1H$ apparent relaxation rate (combination of longitudinal and transverse relaxation rate, $s^{-1}$)

The fitting was performed with Mathematica 8.0. The error for the fitted parameter results from the fitting.

## REFERENCES


(1) Vogtherr, M.; Schubel, H.; Limmer, S. Structural and dynamic helix geometry alterations induced by mismatch base pairs in double-helical RNA. *FEBS Lett* **1998**, *429* (1), 21-26. DOI: 10.1016/s0014-5793(98)00542-0

(2) Huppler, A.; Nikstad, L. J.; Allmann, A. M.; Brow, D. A.; Butcher, S. E. Metal binding and base ionization in the U6 RNA intramolecular stem-loop structure. *Nat Struct Biol* **2002**, *9* (6), 431-435. DOI: 10.1038/nsb800

(3) Keller, H.; Weickhmann, A. K.; Bock, T.; Wohnert, J. Adenine protonation enables cyclic-di-GMP binding to cyclic-GAMP sensing riboswitches. *Rna* **2018**, *24* (10), 1390-1402. DOI: 10.1261/rna.067470.118.

(4) Buttner, L.; Seikowski, J.; Wawrzyniak, K.; Ochmann, A.; Hobartner, C. Synthesis of spin-labeled riboswitch RNAs using convertible nucleosides and DNA-catalyzed RNA ligation. *Bioorg Med Chem* **2013**, *21* (20), 6171-6180. DOI: 10.1016/j.bmc.2013.04.007.

(5) Kabinger, F.; Stiller, C.; Schmitzova, J.; Dienemann, C.; Kokic, G.; Hillen, H. S.; Hobartner, C.; Cramer, P. Mechanism of molnupiravir-induced SARS-CoV-2 mutagenesis. *Nat Struct Mol Biol* **2021**, *28* (9), 740-746. DOI: 10.1038/s41594-021-00651-0.

(6) Lu, J.; Li, N.-S.; Koo, S. C.; Piccirilli, J. A. Efficient synthesis of N4-methyl-and N4-hydroxycytidine phosphoramidites. *Synthesis* **2010**, *2010* (16), 2708-2712.

(7) Neuner, S.; Santner, T.; Kreutz, C.; Micura, R. The "Speedy" Synthesis of Atom-Specific (15)N Imino/Amido-Labeled RNA. *Chemistry* **2015**, *21* (33), 11634-11643. DOI: 10.1002/chem.201501275.

(8) Wishart, D. S.; Bigam, C. G.; Yao, J.; Abildgaard, F.; Dyson, H. J.; Oldfield, E.; Markley, J. L.; Sykes, B. D. 1H, 13C and 15N chemical shift referencing in biomolecular NMR. *J Biomol NMR* **1995**, *6* (2), 135-140. DOI: 10.1007/BF00211777.

(9) Marky, L. A.; Breslauer, K. J. Calculating thermodynamic data for transitions of any molecularity from equilibrium melting curves. *Biopolymers* **1987**, *26* (9), 1601-1620. DOI: 10.1002/bip.360260911.

(10) Hwang, T. L.; Mori, S.; Shaka, A. J.; vanZijl, P. C. M. Application of phase-modulated CLEAN chemical EXchange spectroscopy (CLEANEX-PM) to detect water-protein proton exchange and intermolecular NOEs. *Journal of the American Chemical Society* **1997**, *119* (26), 6203-6204. DOI: DOI 10.1021/ja970160j.

(11) Hwang, T. L.; van Zijl, P. C. M.; Mori, S. Accurate quantitation of water-amide proton exchange rates using the Phase-Modulated CLEAN chemical EXchange (CLEANEX-PM) approach with a Fast-HSQC (FHSQC) detection scheme. *Journal of Biomolecular Nmr* **1998**, *11* (2), 221-226. DOI: Doi 10.1023/A:1008276004875.

(12) Lee, Y. M.; Lee, E. H.; Seo, Y. J.; Kang, Y. M.; Ha, J. H.; Kim, H. E.; Lee, J. H. Measurement of Hydrogen Exchange Times of the RNA Imino Protons Using by Phase-modulated CLEAN Chemical Exchange Spectroscopy. *Bulletin of the Korean Chemical Society* **2009**, *30* (10), 2197-2198.

(13) Strebitzer, E.; Rangadurai, A.; Plangger, R.; Kremser, J.; Juen, M. A.; Tollinger, M.; Al-Hashimi, H. M.; Kreutz, C. 5-Oxyacetic Acid Modification Destabilizes Double Helical Stem Structures and Favors Anionic Watson-Crick like cmo(5)U-G Base Pairs. *Chemistry-a European Journal* **2018**, *24* (71), 18903-18906. DOI: 10.1002/chem.201805077.

(14) Bereiter, R.; Himmelstoss, M.; Renard, E.; Mairhofer, E.; Egger, M.; Breuker, K.; Kreutz, C.; Ennifar, E.; Micura, R. Impact of 3-deazapurine nucleobases on RNA properties. *Nucleic Acids Research* **2021**, *49* (8), 4281-4293. DOI: 10.1093/nar/gkab256.

(15) Hwang, T. L.; Shaka, A. J. Water Suppression That Works - Excitation Sculpting Using Arbitrary Wave-Forms and Pulsed-Field Gradients. *Journal of Magnetic Resonance Series A* **1995**, *112* (2), 275-279. DOI: DOI 10.1006/jmra.1995.1047.

(16) Szulik, M. W.; Voehler, M.; Stone, M. P. NMR analysis of base-pair opening kinetics in DNA. *Curr Protoc Nucleic Acid Chem* **2014**, *59*, 7 20 21-18. DOI: 10.1002/0471142700.nc0720s59.
